# Peer support intervention (ABA-feed) to improve breastfeeding: UK based, multicentre, parallel group, randomised controlled trial

**DOI:** 10.1136/bmj-2025-086558

**Published:** 2026-03-31

**Authors:** Kate Jolly, Joanne Clarke, Nicola Crossland, Stephan U Dombrowski, Eleni Gkini, Pat Hoddinott, Jenny Ingram, Debbie Johnson, Christine MacArthur, Mia Mann, Jennifer McKell, Ngawai Moss, Tracy Roberts, Julia Sanders, Nicola Savory, Alice Sitch, Beck Taylor, Sarah Tearne, Gill Thomson, Eleanor Williams, Rebecca Woolley, ABA-feed study group, Lucy Doos, Raquel Fernandez del Rio, Aisha Khan, Hardeep Sandhar

**Affiliations:** 1 Department of Applied Health Sciences, School of Health Sciences, College of Medicine and Health, University of Birmingham, Birmingham B15 2TT, UK; 2 Maternal, Parental, and Infant Nutrition and Nurture Unit, University of Lancashire, Preston, UK; 3 Faculty of Kinesiology, University of New Brunswick, Fredericton, NB, Canada; 4 Birmingham Clinical Trials Unit, School of Health Sciences, College of Medicine and Health, University of Birmingham, Birmingham, UK; 5 Centre for Healthcare and Community Research, University of Stirling, Stirling, UK; 6 Centre for Academic Child Health, University of Bristol, Bristol, UK; 7 NIHR Birmingham Biomedical Research Centre, University Hospitals Birmingham NHS Foundation Trust and University of Birmingham, Birmingham, UK; 8 Institute for Social Marketing and Health, University of Stirling, Stirling, UK; 9 Patient author, Southend-on-Sea, UK; 10 Health Economics Unit, School of Health Sciences, College of Medicine and Health, University of Birmingham, Birmingham, UK; 11 School of Healthcare Sciences, Cardiff University, Cardiff, UK; 12 Warwick Applied Health, Warwick Medical School, University of Warwick, Coventry, UK

## Abstract

**Objective:**

To assess the effect of a proactive, assets based, peer support infant feeding intervention in addition to usual care on breastfeeding rates, formula feeding practices, and other outcomes, compared with usual breastfeeding support alone.

**Design:**

UK based, multicentre, parallel group, unblinded, randomised controlled trial.

**Setting:**

17 localities in the UK that offered breastfeeding peer support as part of usual care between January 2022 and 30 April 2024.

**Participants:**

2475 nulliparous women between 20 and 35 weeks of gestation were randomised 1.43:1, to account for potential clustering by peer supporter: 1458 to the ABA-feed (Assets based feeding help Before and After birth-feed) peer support intervention and 1017 to usual care.

**Interventions:**

The ABA-feed intervention comprised person centred proactive peer support for infant feeding underpinned by an assets based approach (focusing on the capabilities of, and resources available to, participants) and behaviour change theory delivered in person and remotely by text and telephone call. Usual care included universal care from midwives and health visitors and could also include services that provided reactive support such as peer supporters in breastfeeding groups, counselling, helplines, and social media support groups.

**Main outcome measures:**

The primary outcome was any breastfeeding at eight weeks after birth. Secondary outcomes at eight, 16, and 24 weeks after birth included breastfeeding initiation, any and exclusive breastfeeding, formula feeding practices, anxiety, social support, and healthcare utilisation. Analyses were based on the intention-to-treat principle.

**Results:**

Rates of any breastfeeding at eight weeks did not differ between the intervention group (1013/1452; 69.8%) and usual care group (698/1015; 68.8%); adjusted risk difference 0.01, 95% confidence interval −0.03 to 0.04. Preplanned subgroup analyses showed no interactions between the intervention and age, prespecified feeding intentions, mother’s education, index of multiple deprivation fifth, or relationship status. Breastfeeding initiation rates were high (intervention 94.2%; usual care 92.5%). At eight weeks the intervention group reported higher social support, but this was not sustained at 16 weeks. No differences were observed in other secondary outcomes.

**Conclusion:**

The ABA-feed peer support intervention did not improve breastfeeding rates compared with usual breastfeeding support in a UK context.

**Trial registration:**

ISRCTN Registry ISRCTN17395671.

## Introduction

Both any and exclusive breastfeeding has health benefits for infants, children, and mothers,^1^ whereas suboptimal formula feeding practices increase the risk of infection and overfeeding in babies.^2^ ^3^ The World Health Organization (WHO) recommends exclusive breastfeeding for six months after birth and set a target for this to be achieved for 50% of babies by 2025; worldwide 48% of babies aged 0-5 months were being exclusively breastfed in 2022.^4^ Breastfeeding initiation and continuation rates vary considerably between and within countries,^5^ with low rates of any breastfeeding in high income countries associated with socioeconomic disadvantage, younger age, low educational attainment, and ethnicity.^6^ ^7^ ^8^ In the four UK countries, between 62.5% and 71.7% of babies were reported to have received breast milk at their first feed (2020/21), but by 6-8 weeks any breastfeeding rates had declined noticeably (ranging from 31.6% to 52.7%).^9^ ^10^ ^11^ ^12^

A 2022 Cochrane review reported moderate certainty evidence that interventions providing breastfeeding support (delivered by professionals or non-professionals) probably reduce the risk of women stopping any and exclusive breastfeeding at 4-6 weeks, 3-4 months, and six months after birth.^13^ The synthesised effects showed substantial heterogeneity, largely unexplained by subgroup analyses. No differences were observed by type of person providing the support.^13^ The WHO,^14^ Unicef UK Baby Friendly Initiative,^15^ and UK National Institute for Health and Care Excellence (NICE) recommend peer support for breastfeeding.^16^ ^17^

Despite international evidence for the effectiveness of peer support on any and exclusive breastfeeding rates, several large randomised controlled trials of peer support in the UK did not show a beneficial effect.^18^ ^19^ ^20^ ^21^ Possible reasons include low intensity interventions, lack of timely support after the birth, and the need for a new parent to initiate contact. The ABA-feed (Assets based feeding help Before and After birth-feed) intervention of infant feeding peer support was developed to deal with these issues, drawing on best evidence including a moderate to high number of proactive woman centred antenatal and postnatal contacts,^13^ ^22^ ^23^ underpinned by behaviour change theory and an assets based approach (assets based approaches focus on the positive capabilities of individuals, and personal and community based resources available to them).^24^ ^25^ Novel aspects were its peer delivered contact post-birth that was proactive and daily, and inclusion of all women regardless of feeding intention to improve feeding outcomes for all babies. A feasibility trial showed the acceptability and practicality of delivering the ABA-feed intervention.^26^ We evaluated whether this novel, intensive peer support intervention for infant feeding could improve breastfeeding rates in a UK context.

## Methods

### Design and setting

We conducted a multicentre, parallel group, unblinded, randomised controlled trial with concurrent process and economic evaluations in the UK. The cost effectiveness analysis will be published elsewhere. The methods have been described in detail elsewhere, with no changes to the protocol after publication.^27^

Sites were geographical areas in the UK (local authorities or health boards) with low breastfeeding rates and some provision of breastfeeding peer support (eg, in breastfeeding groups) but no delivery of universal proactive peer support for infant feeding both antenatally and postnatally as part of usual care. Sites delivered peer support services through a range of different providers: National Health Service (NHS) organisations, local authorities, or third sector organisations. The existing peer supporters who were trained to deliver the ABA-feed intervention were termed infant feeding helpers.

### Inclusivity

We did not collect participant information about gender identity, but acknowledge that breastfeeding, woman, and other gendered terms are not the preferred terminology for all individuals who give birth or who feed their infant with their own milk. For brevity, we have used these terms as they are widely used and understood.

### Recruitment and participants

Regardless of their feeding intention, women were eligible for recruitment if they were nulliparous, between 20 weeks and 35 weeks and six days gestation with a singleton pregnancy, aged 16 years or older, and lived in the study area. We excluded non-English speaking women if their locality lacked infant feeding helpers able to speak their language. No other exclusion criteria applied.

The trial commenced during covid-19 pandemic restrictions, so a range of recruitment methods was necessary to maximise participation. These approaches included a summary leaflet handed out by care staff with details on how to register interest in the trial and the option of completing an agreement to contact form; direct invitations in antenatal and 20 week scan clinics, remote invitations included as posters in antenatal clinics and other places frequented by pregnant women, email invitations sent from maternity or health visiting services, and use of social media. Information sources included a QR code linked to a study website that provided information about the trial, and a secure link for women to provide contact details for a member of the study team to discuss the trial in more detail. Before recruitment all women received a detailed information leaflet and had the opportunity to ask questions.

Informed consent was obtained from each participant in person or by telephone or video call. When consent was required remotely, women with computer or smartphone access were given the option to complete an e-consent form. When in-person consent or e-consent was not possible, the researcher initialled, signed, and dated the consent form during a telephone discussion, with a copy sent to the woman.

### Randomisation and masking

After eligibility had been checked, informed consent received, and the baseline questionnaire completed, researchers randomised participants using unique login usernames and passwords. To ensure concealment of treatment allocation, the Birmingham Clinical Trials Unit used a secure, central, web based randomisation system to allocate women in a 1.43:1 ratio to receive intervention or usual care. A minimisation algorithm (with random element) within the online randomisation system ensured balance in treatment allocation for study site and the woman’s age (<25, ≥25 years), given the association between maternal age and breastfeeding.^28^

The trial number and initials of women allocated to the ABA-feed intervention were securely sent to the peer support lead at the relevant site. The peer support lead then allocated the participant to a local infant feeding helper, who was able to check a secure database for the woman’s name and contact details.

### Usual care

NHS hospital and community services aim to provide usual care for infant feeding in line with NICE^17^ and Unicef Baby Friendly guidance.^15^ In the UK this should include universal care from midwives and health visitors. Information and discussion on infant feeding should be provided antenatally by the midwife and at a third trimester contact with the health visitor; antenatal parentcraft sessions may also be offered, with feeding discussed at one session. Postnatally, after support for infant feeding in hospital, a community midwife should provide home (sometimes clinic) visits on days 1, 3, and 5. The community midwife hands over care to the health visitor at around 10-14 days. Usual care could also include services that provide reactive support, such as peer supporters in breastfeeding groups, counselling, helplines, and social media support groups; these were characterised within the process evaluation.

### ABA-feed intervention

The ABA-feed intervention was peer delivered using in-person and remote methods (SMS text, WhatsApp, and telephone calls), with the postnatal contact predominantly remote. ABA-feed was delivered by whichever organisation provided breastfeeding peer support in the locality. Most peers were volunteers; about 10% were employed in this role. The infant feeding helpers had already received training in breastfeeding peer support through local training programmes. A train-the-trainer model was used for ABA-feed, with the infant feeding or peer support leads attending eight hours of remote training with an experienced trainer. Sessions included two hours on study procedures delivered by the research team.^29^ The local infant feeding or peer support leads then trained the infant feeding helpers (eight hours’ duration), including study procedures delivered by the research team.

In additional to usual care, participants allocated to the ABA-feed intervention were offered proactive feeding support, underpinned by behaviour change theory and an assets based approach. The intervention delivered person centred care, was inclusive of all feeding methods,^30^ and was based on best evidence relating to setting, frequency, duration, and type of support from infant feeding helpers. Details of intervention development are published elsewhere.^26^ ^31^

We asked local infant feeding leads to provide information on local assets (ie, antenatal groups, postnatal groups, breastfeeding drop-ins, breastfeeding counsellors, and baby groups) and included these plus details of national helplines and internet resources in a local assets leaflet for each site. The assets leaflet was handed to women during face-to-face contact with their infant feeding helper or sent electronically as well as being posted at 36 weeks’ gestation.

The intervention started after around 30 weeks’ gestation and continued until eight weeks after the birth. Around 30 weeks’ gestation, the infant feeding helper contacted the woman to arrange an antenatal meeting at a convenient location (eg, children’s centre, café, or, if the peer support service permitted this, at home). Alternatively, the meeting could be via video call or telephone if the woman preferred. Women could include partners or family members in this meeting, and during subsequent contacts. The aim was to talk about infant feeding and explore the woman’s assets for feeding, including jointly developing a diagram of friends and family^32^ to facilitate reflection on available sources of support. Infant feeding helpers introduced the assets leaflet, explained the support available for infant feeding, swapped contact details, and asked to be told of the birth to facilitate early postnatal support. After the meeting, infant feeding helpers kept in contact with the participant during pregnancy to encourage rapport and facilitate immediate support after the birth. When possible, infant feeding helpers were asked to offer an antenatal visit to a local breastfeeding group with the woman (if she planned to breastfeed), so women knew where and how to access additional support postnatally. Infant feeding helpers offered daily text or telephone contact with the woman until the baby was 2 weeks old, with less frequent contact up to eight weeks. Frequency of contact was negotiated between the infant feeding helpers and the women and depended on the women’s preferences and support needs.

### Outcomes

The primary outcome was any breastfeeding at eight weeks after giving birth collected by self-report questionnaire. If women did not respond to the relevant item in the questionnaire, we used the feeding data collected routinely by health visitors at 6-8 weeks after birth. Any breastfeeding was defined in accordance with the UK Infant Feeding Survey as “infant being breastfed (including being given expressed breast milk), within the past 24 hours, even if they were also receiving infant formula, solid food or other liquids.”^28^

Secondary outcomes were measured by questionnaire at eight and 16 weeks; a text message at three days and 24 weeks collected information about mode of infant feeding. Secondary outcomes were breastfeeding initiation; any breastfeeding at 16 and 24 weeks after birth; exclusive breastfeeding (defined in accordance with the WHO definition of infants who received only breast milk during the previous 24 hours) at eight, 16, and 24 weeks; time to cease any and exclusive breastfeeding; and diagnosis of tongue tie and whether treated. At eight and 16 weeks only we measured anxiety using the GAD-7 (generalised anxiety disorder 7 item) scale,^33^ health related quality of life using the EQ-5D-5L (EuroQol 5-dimension 5 level questionnaire),^34^ social support (Medical Outcomes Study),^35^ self-reported formula feeding practices (how formula is prepared),^28^ use of support for infant feeding, and maternal and infant healthcare utilisation.

### Adverse events

Given that the intervention was low risk, reporting of serious adverse events was not expedited. We captured any infant deaths with cause from local maternity teams, and collected self-reported data from participants on overnight admissions to hospital of infants and mothers. The data monitoring committee regularly reviewed these data. The local principal investigator checked any notifications of an infant death in which an infant feeding helper was the last healthcare professional or feeding supporter to have been in contact with the woman. The chief investigator then assessed the event for relatedness.

### Statistical analysis

The primary comparison groups were participants randomised to either the ABA-feed intervention plus usual care or usual care only. All analyses were based on the intention-to-treat principle.

For all outcome measures, we present appropriate summary statistics and differences between groups (eg, means, relative risks), with 95% confidence intervals (CIs) and P values from two sided tests. We did not adjust for multiple comparisons. Statistical analysis was undertaken using the statistical software packages SAS version 9.4 and Stata version 18.

The primary outcome (any breastfeeding at eight weeks after birth) was binary, and the planned analysis was to use a mixed effects log binomial regression model, adjusting for intervention group and minimisation variables (age group and site) and the interaction of intervention by infant feeding helper. In the first instance, we treated age (a continuous variable) as a fixed effect, site as a random effect, and infant feeding helper as a partial random effect. Owing to issues with convergence, we included age in the model as the categorised minimisation variable. Infant feeding helper was removed as a partial random effect because of problems with convergence or the estimate was close to zero. The treatment effect was expressed as an adjusted risk ratio and a risk difference with associated 95% CIs. Missing data were imputed as formula feeding for the primary outcome, excluding pregnancy losses, still births, infant deaths, or maternal deaths. Various prespecified analyses were undertaken: a per protocol analysis (the minimal level of contact determined as a participant in the intervention group receiving the initial main meeting and at least one postnatal contact, and a participant in the usual care group having no contact with an infant feeding helper) and missing data assumptions (eg, complete case analysis, imputing the missing responses using a tipping point analysis) on the primary analysis.^36^ Prespecified subgroup analyses were limited to the primary outcome only and undertaken using age, feeding intention, mother’s education, index of multiple deprivation fifth, and relationship status.

Binary secondary outcomes (eg, breastfeeding initiation) were analysed using the same methods as for the primary outcome, with missing data imputed as formula feeding for any breastfeeding at 16 weeks after birth and any breastfeeding at 24 weeks after birth. Time-to-cease outcomes were analysed using Cox proportional hazards models adjusting for the minimisation variables according to the primary outcome. Kaplan-Meier survival curves were constructed for visual presentation of time-to-event comparisons. For those continuous secondary outcomes (eg, anxiety measured by the GAD-7 at eight and 16 weeks) we used mixed effects linear regression methods to estimate treatment effects adjusting for minimisation variables (with age as a continuous variable) and baseline measures when relevant. To assess the impact of outliers or skewed data, we conducted sensitivity analyses either by excluding the outliers or by using bootstrapping methods to estimate unadjusted median differences in cases of skewed data. Further analysis details are in the statistical analysis plan (see supplementary file). Primary and secondary outcome data were kept separate from process evaluation data for analysis.

Summary statistics detailing how the intervention was delivered were produced using data from the infant feeding helper logs. Minimal contact was predefined as the antenatal contact plus at least one postnatal contact. Levels of postnatal contact were categorised as low (<4 contacts), medium (4-8 contacts), and high (>8 contacts).

### Sample size

We considered an increase of 7% in breastfeeding at eight weeks to be clinically meaningful.^37^ ^38^ Assuming 90% power and a two sided 5% significance level, with a control group rate of 44% for the primary outcome (95% CI 30.0% to 58.7%; from ABA feasibility data), a sample size of 2136 women (1068 in each group) was required to detect a risk ratio of 1.16 (ie, an increase of 7%). To allow for potential clustering of outcomes by infant feeding helper, we inflated the sample size for the intervention arm. We assumed an intracluster correlation coefficient of 0.039 (from the ABA feasibility data) and that each infant feeding helper would support about 12 women. The sample size required for the intervention arm was 1526, giving a total sample size of 2594. Allowing for a 5% loss to follow-up,^27^ 2730 (1606 intervention and 1124 control arm) women were required. Assuming 80% power, the sample size of 2730 allowed for detection of a risk ratio of 1.14, equivalent to a 6% absolute increase. On the basis that each infant feeding helper supported up to 12 women, we needed to train a minimum of 134 peer supporters.

### Process evaluation and trial oversight

The process evaluation data included in this paper are infant feeding helper intervention logs (recording number and timing of contacts with women) and document review and brief interviews with infant feeding leads to map usual care. We used these data to assess key components of the intervention logic model^27^ that describe intervention delivery: meeting in antenatal period, contact within 48 hours of birth, and daily contact during the first two weeks after the birth.

An independent trial steering committee provided trial oversight and included a parent who had breastfed and a stakeholder who had run a breastfeeding peer support service. An independent data monitoring committee assessed progress of the trial, data quality, and safety data, and could recommend to the trial steering committee whether to continue, modify, or stop the trial.

### Patient and public involvement

This study was in response to a commissioned call from the National Institute for Health and Care Research-Public Health Research, which has stakeholder involvement in topic selection. Members of the public were involved in the development of the intervention and in the design and monitoring of the feasibility study. A public involvement co-investigator was involved in the development of the ABA-feed protocol and attended co-investigator meetings with a second public contributor. A public advisory group (comprising six mothers with a range of infant feeding experiences from two areas of the UK) met regularly with researchers to provide input and feedback into trial processes and to ensure that the proposed intervention, methods of communication, and data collection were appropriate and that the study findings would be relevant and beneficial to our target population. Changes made after suggestions from the public advisory groups included production of a summary version of the public information leaflet and addition of research team information on the study website to enhance credibility.

## Results

### Characteristics of participants and sites

**Fig 1:**
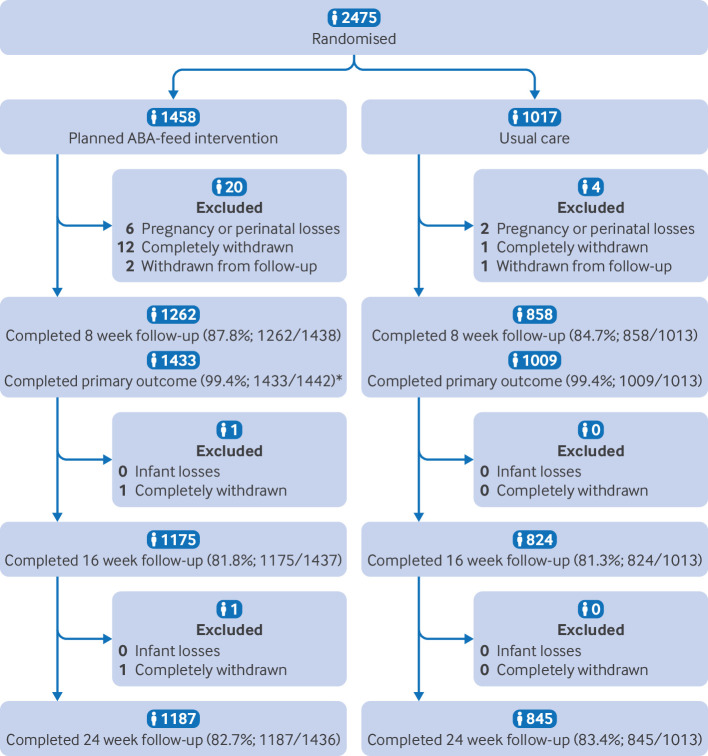
Participant flow through trial. *As the primary outcome was also obtained from health visitor records, the expected figure includes all randomised participants who had not fully withdrawn and had provided their baby’s date of birth

Overall, 2475 participants were recruited from 17 sites across the UK between January 2022 and January 2024 and randomised to usual care plus ABA-feed (n=1458) or to usual care only (n=1017). Supplementary table 1 provides details of usual care. Recruitment commenced during the covid-19 pandemic, with restrictions to in-person recruitment. At final recruitment, 90.7% of the 2730 target population was achieved (fig 1).

Baseline characteristics of the participants were similar between the two groups (table 1). Mean age of the participants was 30 years, and most described their ethnicity as white (2132; 86.5%), family status as married or cohabiting (2309; 94.7%), employment status as in work (2317; 93.9%), and education to degree level (1726; 70.2%). Overall, 111 (4.5%) planned to use only formula milk during the first six months after birth.

**Table 1:** Baseline characteristics of participants by study group. Values are number (percentage) unless stated otherwise

Characteristics	ABA-feed (n=1458)	Usual care (n=1017)
**Participant characteristics**		
Mean (SD) age (years)	30.6 (4.7)	30.6 (4.8)
Age group (years):		
16-24	137 (9.4)	96 (9.4)
≥25	1321 (90.6)	921 (90.6)
Ethnic group:		
White British	1180 (81.3)	808 (79.8)
White other	77 (5.3)	67 (6.6)
Mixed or multiple ethnic groups	53 (3.6)	35 (3.4)
Asian or Asian British	78 (5.4)	64 (6.3)
Black, African, Caribbean, or black British	55 (3.8)	29 (2.9)
Other	9 (0.6)	10 (1.0)
Prefer not to say or missing	6	4
Highest level of qualification:		
No formal qualification	5 (0.3)	4 (0.4)
GCSE, standard grade, national 5 or equivalent	144 (10.0)	101 (10.0)
A level, AS level, highers or equivalent	251 (17.4)	165 (16.3)
Degree level or above	1013 (70.2)	713 (70.3)
Other	31 (2.1)	31 (3.1)
Missing	14	3
Family status:		
Married or in a registered civil partnership	718 (50.0)	492 (49.0)
Cohabiting	648 (45.1)	451 (45.0)
Single	70 (4.9)	58 (5.8)
Widowed, divorced, or separated	0 (0)	2 (0.2)
Prefer not to say or missing	22	14
Median (IQR) No of additional adults in household	1.0 (1.0-1.0)	1.0 (1.0-1.0)
Current work situation*:		
Paid full time or part time (including self-employed)	1362 (93.8)	955 (94.9)
Unemployed or looking for work	40 (2.8)	27 (2.7)
Looking after family or home	19 (1.3)	19 (1.9)
Unable to work because of long term health problem	12 (0.8)	6 (0.6)
In full time education or training	34 (2.3)	24 (2.4)
Other	8 (0.6)	7 (0.7)
Missing	6	1
**Infant feeding information**		
What milk do you want to give baby during the first six months of his/her life?:		
Breast milk only	692 (47.6)	510 (50.1)
Mainly breast milk	468 (32.2)	301 (29.6)
Half and half breast and formula milk	187 (12.9)	133 (13.1)
Mainly formula milk	38 (2.6)	30 (2.9)
Formula milk only	68 (4.7)	43 (4.2)
Missing	5	0
How were you fed as a baby?:		
Breast fed entirely	567 (39.0)	409 (40.3)
Formula fed entirely	441 (30.3)	309 (30.4)
Both breast and formula fed	295 (20.3)	213 (21.0)
Do not know	152 (10.4)	85 (8.4)
Missing	3	1
**Area characteristic**		
Deprivation index fifth†:		
1 (most deprived)	285 (19.6)	205 (20.3)
2	299 (20.6)	209 (20.7)
3	305 (21.1)	195 (19.3)
4	339 (23.3)	232 (23.0)
5 (least deprived)	224 (15.4)	168 (16.7)
Missing	6	8

All percentages presented do not include missing data.

ABA-feed=Assets based feeding help Before and After birth-feed; AS=advanced subsidiary; GCSE=general certificate of secondary education; IQR=interquartile range, SD=standard deviation.

* Responses are not mutually exclusive; percentages may total >100%.

† Derived from participant’s postcode.

### Intervention training and delivery

We trained 30 peer support managers and infant feeding leads to deliver ABA-feed intervention training to peer supporters in their locality. Overall, 193 peer supporters were trained to become infant feeding helpers and 177 (91.7%) (160 volunteers, 17 paid) supported a median of 7 (IQR 3-12, range 1-33) participants in the intervention group.

Thirteen participants in the intervention group were not allocated an infant feeding helper owing to withdrawing from the trial (n=8) or site error (administrative mistake) (n=5); one participant in the usual care group was incorrectly allocated an infant feeding helper. Infant feeding helpers completed contact logs for 1257 (86.2%) of participants in the intervention group. They reported the antenatal meeting with 1033 women (82.2%), contacted 910 (72.4%) women within 48 hours of birth, and made contact on a median of 9 (IQR 4-12) days in the 14 days after birth. Table 2 provides more details on intervention delivery. Supplementary table 2 reports the intervention delivery by prespecified subgroups: age group, education, feeding intention, and socioeconomic deprivation. Younger women, those living in more disadvantaged localities and who planned to fully or mainly formula feed had lower contact with their infant feeding helper, including contact within 48 hours of birth, which, at most sites, largely relied on participants notifying their infant feeding helpers that they had given birth.

**Table 2:** Delivery of ABA-feed intervention. Values are number (percentage) unless stated otherwise

Intervention component	Estimate
Trained IFHs	193
IFH with record of supporting at least one participant	177 (91.7)
Median (IQR) No of participants supported per IFH	7.0 (3-12)
Participants with main or initial meeting	1033 (82.2)
Participants with a least one antenatal contact	1129 (89.8)
Participants with a least one postnatal contact	1135 (90.3)
Participants contacted within 48 hours of birth	910 (72.4)
Median (IQR) No of days contacted <14 days of birth	9 (4-12)
Median (IQR) No of contacts in postnatal period	15 (8-20)
Frequency of postnatal contacts:	
Low (<4)	186 (14.8)
Medium (4-8)	143 (11.4)
High (>8)	917 (73.0)
Median (IQR) No of contacts across antenatal and postnatal period	19 (12-24)

ABA-feed=Assets based feeding help Before and After birth-feed; IFH=infant feeding helper; IQR=interquartile range.

### Breastfeeding outcomes

Rates of any breastfeeding at eight weeks (primary outcome) were similar between the intervention group and usual care group (69.8% *v* 68.8%; risk difference 0.01, 95% CI −0.03 to 0.04, P=0.69; intracluster correlation coefficient 0.036) (table 3). Subgroup analyses were performed and absolute differences of 6-9% were seen in the primary outcome between the intervention and usual care groups in subgroups of women with GCSE or A level as highest education level, living in areas in the most deprived fifth of index of multiple deprivation, and who planned to mixed feed (half and half breast and formula milk) (see supplementary table 3). There were no statistically significant interactions between the intervention and subgroups. Contact with an infant feeding helper was reported for 1257 (86.2%) women in the intervention group. Of these, 960 (76.4%) were adherent (ie, had their main initial meeting and at least one postnatal contact with the infant feeding helper). One non-adherent participant in the usual care group had contact with an infant feeding helper. The per protocol analysis identified a larger, but non-significant difference (74.0% intervention *v* 68.7% usual care; risk difference 0.03, 95% CI −0.01 to 0.07; P=0.10). Sensitivity analyses (ie, complete case analysis, worst case scenario, best case scenario, and tipping point approach) supported the findings of the primary outcome analysis (see supplementary table 4 and supplementary figures 1 and 2).

**Table 3:** Infant feeding outcomes by study group. Values are number (percentage) unless stated otherwise

Outcomes	ABA-feed (n=1458)	Usual care (n=1017)	Risk ratio* (95% CI; P value)	Risk difference† (95% CI; P value)
**Primary outcome**				
Any breastfeeding at 8 weeks after birth	1013 (69.8)	698 (68.8)	1.01 (0.96 to 1.06; 0.77)	0.01 (−0.03 to 0.04; 0.69)
**Secondary outcomes**				
Breastfeeding initiation	1239 (94.2)	844 (92.5)	1.02 (1.00 to 1.04; 0.13)	0.02 (−0.004 to 0.04; 0.13)
Any breastfeeding (weeks):				
16	825 (56.8)	560 (55.2)	1.01 (0.95 to 1.08; 0.72)	0.02 (−0.02 to 0.06; 0.37)
24	717 (49.4)	515 (50.7)	0.96 (0.89 to 1.03 0.29)	−0.01 (−0.05 to 0.03; 0.56)
Exclusive breastfeeding (weeks):				
8	667 (53.5)	438 (51.8)	1.02 (0.95 to 1.11; 0.55)	0.02 (−0.03 to 0.06; 0.41)
16	564 (48.2)	395 (48.1)	0.99 (0.91 to 1.08; 0.83)	0.001 (−0.04 to 0.05; 0.95)
24	324 (27.4)	239 (28.4)	0.96 (0.84 to 1.11; 0.59)	−0.01 (−0.05 to 0.03; 0.55)
Median (IQR) time to cease any breastfeeding <16 weeks (days)	21 (2-49)	16.5 (1-42)	0.95‡ (0.82 to 1.10; 0.48)	
Median (IQR) time to cease exclusive breastfeeding <16 weeks (days)	3 (1-28)	2 (1-22)	1.01‡ (0.90 to 1.14; 0.83)	

Both risk ratios and risk differences were adjusted for minimisation variables (mother’s age as a categorical fixed effect and site as categorical random effect (due to convergence issues with the breastfeeding initiation outcome site was removed from this model); infant feeding helper was removed due to convergence issues).

ABA-feed=Assets based feeding help Before and After birth-feed; CI=confidence interval; IQR=interquartile range.

* Value >1 favours ABA-feed intervention.

† Value >0 favour favours planned ABA-feed intervention.

‡ Hazard ratio adjusted for all minimisation variables (age as a continuous variable and site as random effect; infant feeding helper partial random effect was excluded from the model because it was close to zero), a value <1 favours planned ABA-feed intervention.

Breastfeeding initiation rates were high (intervention 94.2%; usual care 92.5%) with no significant difference between the groups (risk difference 0.02, 95% CI −0.004 to 0.04; P=0.13). Any breastfeeding at 16 and 24 weeks and exclusive breastfeeding rates at eight, 16, and 24 weeks were similar between the groups (table 3).

Tongue tie was reported in 254 (20.3%) babies in the intervention group and 150 (17.7%) in the usual care group at eight weeks. Of these, 156 (61.9%) and 86 (57.3%), respectively, had a frenotomy procedure.

### Other secondary outcomes

Anxiety (GAD-7 score at eight weeks) was significantly lower in the intervention group (median 3.0 (IQR 1.0-6.0)) compared with the usual care group (4.0 (1.0-7.0)) using the bootstrapping method owing to the highly skewed data; median difference −1.0 (95% CI −1.96 to −0.04, P=0.04). Social support using the Medical Outcomes Study score was higher in the intervention group (90.6 (75.0-100) *v* 84.4 (71.9-100); also assessed using the bootstrapping method owing to the highly skewed data; median difference 6.25 (95% CI 1.99 to 10.51, P=0.004). These significant differences were not maintained at the 16 week postnatal time point. No significant differences were observed in the health related quality of life outcome (EQ-5D-5L) at eight or 16 weeks (table 4). Sensitivity analysis excluding the outliers showed no effect on the results (see supplementary table 8).

**Table 4:** Secondary outcomes in study groups at eight and 16 weeks

Outcomes	Mean (SD); No		Mean difference* (95% CI; P value)		Median (IQR)		Median difference† (95% CI; P value)
	ABA-feed (n=1458)	Usual care (n=1017)			ABA-feed (n=1458)	Usual care (n=1017)	
**GAD-7 score‡**							
Baseline	3.3 (3.5); 1447	3.5 (3.8); 1005	NA		2.0 (1.0-5.0)	2.0 (1.0-5.0)	NA
8 weeks	4.4 (4.2); 1232	4.7 (4.6); 835	−0.18 (−0.51 to 0.15; 0.29)		3.0 (1.0-6.0)	4.0 (1.0-7.0)	−1.0 (−1.96 to −0.04; 0.04)
16 weeks	4.3 (4.4); 1152	4.4 (4.5); 803	−0.12 (−0.48 to 0.23; 0.50)		3.0 (1.0-6.0)	3.0 (1.0-6.0)	0.0 (−0.83 to 0.83; 1.00)
**EQ-5D-5L index score§**							
Baseline	0.85 (0.14); 1449	0.85 (0.14); 1015	NA		0.84 (0.77-1.0)	0.84 (0.77-1.0)	NA
8 weeks	0.86 (0.14); 1249	0.86 (0.14); 846	0.004 (−0.01 to 0.02; 0.44)		0.88 (0.77-1.0)	0.85 (0.77-1.0)	0.03 (−0.003 to 0.06; 0.07)
16 weeks	0.87 (0.15); 1163	0.87 (0.14); 821	0.002 (−0.01 to 0.01; 0.79)		0.88 (0.77-1.0)	0.88 (0.80-1.0)	0 (−0.0001 to 0.0001; 1.00)
**Medical Outcomes Study score¶**							
Baseline	89.3 (16.2); 1457	89.3 (15.9); 1017	NA		96.9 (84.4-100.0)	96.9 (81.3-100.0)	NA
8 weeks	83.1 (19.6); 1257	80.4 (21.6); 855	2.53 (0.95 to 4.11; 0.002)		90.6 (75.0-100)	84.4 (71.9-100)	6.25 (1.99 to 10.51; 0.004)
16 weeks	81.5 (22.1); 1172	80.9 (22.5); 822	0.83 (−0.90 to 2.56; 0.35)		90.6 (71.9-100)	87.5 (71.9-100)	3.13 (−1.45 to 7.70; 0.18)

ABA-feed=Assets based feeding help Before and After birth-feed; CI=confidence interval, GAD-7=generalised anxiety disorder 7 item scale; EQ-5D-5L=EuroQol 5-dimension 5 level questionnaire; IQR=interquartile range; NA=not applicable; SD=standard deviation.

* Mean difference adjusted for mother’s age as a continuous, site as a categorical random effect (infant feeding helper was removed due to convergence issues), and the baseline score as a continuous variable, a value <0 favours intervention.

† Unadjusted differences in medians using bootstrapping methods (repetition=1000, seed=150 824), a value <0 favours intervention.

‡ Score ranges from 0 to 21; 0 indicates lack of anxiety and 21 indicates highest level of anxiety.

§ Score was calculated using the mapping function developed by Van Hout et al 2012 and the Crosswalk value sets for the UK^39^; value 1 indicates no problems on any of the five dimensions.

¶ Consists of only the emotional/informational dimension (score ranges from 0 to 100); 0 indicates lower level of support and 100 indicates higher level of support.

Supplementary table 5 provides details of the self-reported formula feeding practices. When formula preparation was relevant in the intervention and usual care groups, adherence to recommendations was low for correct water temperature (45.4% *v* 49.3%) and making up formula to use away from the home (34.6% *v* 32.8%). Keeping milk chilled when out of the home differed (33.7% *v* 50.0%) at eight weeks’ follow-up; however, at 16 weeks the difference between groups was similar (51.3% *v* 46.3%).

### Healthcare utilisation

The proportion of babies admitted to hospital for any cause was similar between the two groups (table 5). Data for visits to emergency departments and consultations with general practitioners, midwives, and health visitors were similar between the groups (see supplementary table 6 for details in infants and mothers).

**Table 5:** Hospital admissions of infants up to 16 weeks after birth by study group

Period after birth	No admitted/No in group (%)		Risk ratio* (95% CI; P value)	Risk difference† (95% CI; P value)
	ABA-feed (n=1458)	Usual care (n=1017)		
<8 weeks	143/1251 (11.4)	99/854 (11.6)	-	-
8-16 weeks	30/1165 (2.6)	21/821 (2.6)	-	-
<16 weeks	165/1149 (14.4)	116/801 (14.5)	0.99 (0.80 to 1.24); 0.94	−0.001 (−0.03 to 0.03); 0.94

Both risk ratios and risk differences were adjusted for mother’s age as a categorical fixed effect (site and infant feeding helper were removed due to convergence issues).

ABA-feed=Assets based feeding help Before and After birth-feed; CI=confidence interval.

* Value <1 favours planned ABA-feed intervention.

† Value <0 favours planned ABA-feed intervention.

### Maternal use of support for infant feeding

Participants in both groups reported that most of the discussions on feeding in the eight weeks after birth were with midwives and health visitors. The proportion of women obtaining support from any type of health professional was similar between the study groups. Overall, 157 (18.5%) participants in the usual care group and 193 (22.8%) in the intervention group reported feeding support from an infant feeding counsellor or breastfeeding supporter (not ABA-feed infant feeding helper). Only 106 (8.5%) respondents in the intervention group reported no support from their infant feeding helper. A higher proportion of those in the ABA-feed group reported drawing on support from family members (924 (73.9%) *v* 847 (66.9%)) and friends (915 (73.3%) *v* 556 (65.7%)) at eight weeks (supplementary table 7).

### Adverse events

During the study, no pregnancy losses occurred, four babies were stillborn, and four pregnancies resulted in early neonatal deaths. No serious adverse events occurred that were considered related to the intervention.

## Discussion

The ABA-feed randomised controlled trial implemented a universally offered, intensive, proactive infant feeding peer support intervention, commencing in pregnancy and continuing up to eight weeks after birth. Contrary to our hypothesis, we found no statistically significant between group differences in our primary outcome of any breastfeeding at eight weeks, or for other feeding outcomes. At eight weeks, social support was higher and anxiety scores lower in the intervention group, but differences were modest^40^ and not sustained at 16 weeks.

### Comparison with other studies

Our findings are not consistent with the 2022 Cochrane systematic review of support for breastfeeding mothers, which reported a statistically significant improvement in breastfeeding outcomes from support interventions at most time points across the global literature, although not for any breastfeeding at two months, the primary outcome of our study.^13^ Meta-regression showed no difference in outcomes by type of individual delivering the support (non-professional or healthcare professional).^13^ Previous trials of breastfeeding peer support have generally recruited women who have already commenced breastfeeding or intended to breastfeed^37^ ^41^; this contrasts with the current trial, which recruited women during pregnancy regardless of feeding intention and aimed to increase breastfeeding initiation as well as continuation. A previous UK trial showed no improvement in breastfeeding initiation,^19^ and a systematic review concluded that antenatal peer support delivered universally (not targeted to those planning to breastfeed) was ineffective.^42^ Our results support these findings, despite enhancing the peer support intervention to address limitations of previous studies.

Most of the postnatal ABA-feed support was delivered remotely. Evidence on delivery mode is mixed; a systematic review of remote provision of breastfeeding support and education found no difference in stopping any breastfeeding at 4-8 weeks (risk ratio 1.10, 95% CI 0.74 to 1.64; 15 trials), three months, or six months, in line with our findings, but reported a significantly reduced risk of women stopping exclusive breastfeeding at three months (risk ratio 0.75, 95% CI 0.63 to 0.90).^43^ The 2022 Cochrane review meta-regression, however, found no difference in any breastfeeding at 4-6 weeks and six months by type of support (face-to-face, telephone, or digital).^13^ An Australian trial of postnatal telephone based breastfeeding peer support (RUBY),^37^ reported a 6% absolute difference in infants receiving any breast milk at 6 months of age,^37^ but in contrast with ABA-feed, RUBY recruited women postnatally who had already commenced breastfeeding.

The ABA-feed intervention was intensive, with a median of 19 contacts reported which the qualitative data showed to be acceptable to women and infant feeding helpers.^44^ The 2022 Cochrane review reported that moderate intensity (4-8 planned contacts) was possibly more effective than less or more intensive interventions.^13^ ^37^ However, most trials of peer support interventions delivered globally, particularly those delivered in resource poor settings,^45^ ^46^ ^47^ ^48^ ^49^ have not reported adherence to the support protocol. Our trial found a modest but statistically significant difference in maternal anxiety at eight weeks favouring the intervention group, possibly mediated by increased perceived social support. Findings on whether anxiety is associated with breastfeeding are mixed.^13^ ^50^ ^51^ ^52^ Although perceived social support has been associated with breastfeeding self-efficacy and planned breastfeeding, a lack of association with breastfeeding outcomes has been reported.^52^ ^53^

The ABA-feed intervention differed from those of most other peer support feeding trials, particularly in resource poor settings, in that it was conducted in the UK setting with universal community based midwifery and health visiting services and offered support for women regardless of their feeding intention.^13^ Despite this support for formula feeding, however, we found self-reported formula preparation practices to be no better than in the usual care group, suggesting mothers may find implementing the recommendations challenging. Compared with the 2010 Infant Feeding Survey, our findings showed improvements in the proportion of women reporting that feeds were made up one at a time (87.7% in our usual care group compared with 71% in the survey), but a reduction in those reporting the correct water temperature (49.3% in our usual care group compared with 71% in the survey),^28^ which could be related to the increasing popularity of using machines to prepare formula milk. Qualitative research with UK women who formula feed highlights unmet support needs.^54^ ^55^ ^56^ Most trials of peer support in low and middle income countries report exclusive breastfeeding as the primary outcome,^45^ ^46^ ^47^ ^48^ because, unlike in the UK, safe use of any formula feeding in such settings can be challenging. This may be a simpler message, and a more acceptable one for a peer to deliver, as many are highly motivated to support continuation of breastfeeding. Lastly, the assets based approach of the intervention was novel within trials of infant feeding interventions.^13^ Although some other interventions, such as those offering access to breastfeeding support groups or family support, may have had elements of an assets based approach, no other trials have included this as an underpinning theoretical approach.

### Strengths and limitations of this study

Strengths of the ABA-feed study include its large size, pragmatic design, process evaluation, and implementation in 17 localities across England, Wales, and Scotland, giving strength to the validity of the findings and to generalisability. In contrast with other breastfeeding peer support trials, ABA-feed recruited women regardless of feeding intention and in line with recent NICE guidance,^17^ providing information about best formula feeding practices for women who decide to formula feed. We inflated the intervention group sample size to take account of potential clustering by infant feeding helper, and our intracluster correlation coefficient was 0.036, close to that used in the power calculation. The high proportion of participants with the primary outcome provides confidence in our findings. While women may have been more likely to report breastfeeding to their health visitors due to social desirability bias, this would have affected both trial arms similarly and related to only 13.7% of primary outcome observations. In contrast with some other trials,^18^ ^37^ ^57^ ^58^ ^59^ our train-the-trainer model enabled us to test implementation as it would occur in practice across a diversity of settings, providers, and geographical spread. The intervention was delivered with good fidelity and without evidence of contamination. In line with recommendations from the Cochrane systematic review,^13^ we have reported detailed information about the intervention and settings and reported the fidelity of intervention delivery.^60^ Additionally, we assessed participant and peer supporter experiences qualitatively^44^ and undertook a cost effectiveness analysis that will be reported elsewhere.

A limitation was that we did not fully achieve recruitment numbers to reach 90% power but did exceed the target for 80% power. In common with other trials of peer support,^37^ our sample showed evidence of recruitment bias, with educated women over-represented, likely due to restrictions to in-person recruitment during the covid-19 pandemic.^26^ The use of social media and other remote invitations meant that we could not calculate a recruitment rate. The pandemic also affected the availability of community based infant feeding support, which gradually restarted during the study period, although breastfeeding rates were largely unaffected.^61^ The breastfeeding initiation rate was high in both study groups, potentially explained by the population recruited, as more highly educated women are more likely to breastfeed.^62^ This high breastfeeding rate and educated population recruited suggest that participants were more motivated to breastfeed, limiting our ability to draw conclusions about the effectiveness of peer support on breastfeeding in a population with lower levels of formal education with lower breastfeeding rates. Consent and randomisation may be barriers to women from more disadvantaged communities joining trials, resulting in trial populations with a high motivation to breastfeed as reported in a previous UK trial.^18^ Our inability to include women regardless of their spoken language means that results cannot be generalised to non-English speaking women. We obtained data from infant feeding helpers on contacts with the women they supported for 86.2% of the participants in the intervention group. Previous trials have reported incomplete recording of activity by peer supporters,^18^ ^41^ ^63^ and it is possible that our contact data did not fully reflect the experience for all women. As we cannot fully rule out contamination, we put in place measures to reduce and identify it. A small number of women from the usual care group reported discussing infant feeding with an ABA-feed infant feeding helper, which may have occurred in a breastfeeding group setting with a paid peer supporter who continued to offer peer support to participants not receiving the intervention. It is possible that the content of the assets leaflets was shared more widely in social networks, but we did not identify any reports of women from the usual care group using these in our extensive process evaluation.

### Implications for clinicians and policymakers

No randomised controlled trial of breastfeeding peer support in the UK has shown peer support to be effective.^18^ ^19^ ^21^ ^58^ ^59^ ^64^ Peer support for breastfeeding is recommended in the UK,^15^ ^16^ ^17^ so usual care for ABA-feed sites included some availability of peer support, usually in breastfeeding groups, thus the trial did not address the question of the effectiveness of peer support that was only postnatal, not intensive, and not proactive; this has previously been answered. ABA-feed was designed to address the limitations of former UK trials through including an antenatal component, increasing the intensity, and providing proactive support, but also showed no effect on breastfeeding outcomes.^13^ ^65^ The implications are that universal intensive peer support for infant feeding would not be recommended in the UK context.

The intensity of the ABA-feed intervention has implications for the sustainability of ABA-feed as a predominantly volunteer delivered intervention. The median number of women supported for each infant feeding helper was seven, rather than the 12 as planned, and the qualitative process evaluation identified that many infant feeding helpers considered the intervention to be more suitable to a paid role. Research exploring reasons for volunteers ceasing their role cites high demands as well as personal commitments and circumstances,^66^ which matched reasons volunteers shared with their managers in our study. A UK focused health economic model conducted to inform NICE guidelines concluded that an antenatal and postnatal education and support intervention delivered by a mix of healthcare professionals and peer supporters was unlikely to be cost effective.^16^ ^67^ International evidence suggests that peer support is more effective in promoting exclusive breastfeeding in those who have already commenced breastfeeding or intend to breastfeed, rather than maximising any breast milk, suggesting this might be the focus of peer support in the UK and high income settings.^13^

The high rates of initiation and continuation of breastfeeding in our usual care group suggest that women who are motivated to breastfeed and have feeding support available from healthcare professionals and possibly also some postnatal peer supporters can achieve high rates of breastfeeding. Subgroup analyses were conducted to generate hypotheses of groups that may benefit from the intervention. Although interaction tests did not show statistically significant results, these were under-powered. The higher rates of any breastfeeding at eight weeks in women with lower educational achievements and those living in areas of high socioeconomic deprivation raise the possibility that the ABA-feed intervention might be effective in targeted groups, where background rates of breastfeeding are lower.

### Future research

The lack of effect on infant feeding outcomes from this multicentre randomised controlled trial highlights the need to undertake further research focused on underserved communities and other populations least likely to breastfeed. Research addressing structural and cultural barriers to breastfeeding^68^ is needed. Evaluations of feeding support interventions should include analyses by educational achievement and socioeconomic deprivation to ensure that such interventions do not widen inequalities.

### Conclusions

The ABA-feed model of enhanced peer support did not show any benefits to breastfeeding rates or formula feeding practices in the UK. In the context of previous UK trials of peer support interventions showing no improvement in breastfeeding rates, this additional evidence would not support the commissioning of universal high intensity one-to-one peer support interventions. Approaches targeted to populations with low breastfeeding rates should be explored, with robust evaluation.

**What is already known on this topic**

Systematic reviews report that breastfeeding peer support improves both any and exclusive breastfeeding rates
Remotely delivered breastfeeding peer support can be effective at improving exclusive breastfeeding but is less likely to be effective for improving any breastfeeding
No UK trial has shown a benefit of peer support on feeding outcomes, but previous interventions were low intensity, relied on new parents to ask for support, and contact after the birth was often delayed

**What this study adds**

This randomised trial provided evidence that enhanced peer support in addition to usual care was no more effective than usual care alone for improving breastfeeding and would not support health service commissioning of universal high intensity one-to-one peer support interventions
The trial delivered intensive, proactive, and timely peer support for women regardless of their antenatal feeding intention, with predominantly remote support postnatally
Countries considering infant feeding peer support programmes are recommended to establish effectiveness in their local context with possible targeting including robust evaluation in groups least likely to breastfeed

## Data Availability

The SAS version 9.4 and Stata version 18 statistical analysis code is included in the supplementary file. Deidentified individual participant data stored at https://edata.bham.ac.uk/1555/ and are fully accessible for ethically approved research after registration with UBIRA eData.

## Web extra

Extra material supplied by authors

Supplementary information: Supplementary tables 1-8, figures 1 and 2, statistical analysis plan, and statistical analysis codes

## Notes

Contributors: KJ drafted the paper, with critical input from all authors. KJ, PH, JI, GT, CM, AS, TR, SD, and JC contributed to the inception of the study. JC, SD, EG, PH, JI, CM, NM, LO, TR, GT, JS, AS, CS, BT, ST, RW, and KJ contributed to study design and development. ST, RF, LD, and AK contributed to study management. EG and RW undertook the statistical analyses. EW and TR undertook the health economics components. JC, NC, NS, JM, and DJ undertook the process evaluation with input from GT, KJ, BT, SD, PH, JI, JS, NM, and CM. AS and MM led the process evaluation quantitative analyses with input from KJ and JC. NM was the public involvement lead supported by JC. All authors approved the final manuscript. KJ is the guarantor. The corresponding author attests that all listed authors meet authorship criteria and that no others meeting the criteria have been omitted.

Transparency: The lead author (the manuscript’s guarantor) affirms that the manuscript is an honest, accurate, and transparent account of the study being reported; that no important aspects of the study have been omitted; and that any discrepancies from the study as planned (and, if relevant, registered) have been explained.

Dissemination to participants and related patient and public communities: Once the results are published, we plan to disseminate the results widely to participants, participating sites, related public communities, public health commissioners, and infant feeding providers through our university websites, social media, and with infant feeding organisations.

Provenance and peer review: Not commissioned; externally peer reviewed.
