# Supplementary material for: Peer support intervention (ABA-feed) to improve breastfeeding: UK based, multicentre, parallel group, randomised controlled trial
Source: BMJ. 2026 Mar 31;392:e086558. doi: 10.1136/bmj-2025-086558 (PMC13036591; doi:10.1136/bmj-2025-086558)
Supplement: Supplementary file 1 — Supplementary information: Supplementary tables 1-8, figures 1 and 2, statistical analysis plan, and statistical analysis codes [file jolk086558.ww.pdf]

**Peer support intervention (ABA-feed) for improving breastfeeding: UK based, multicentre, parallel group, randomised controlled trial**

**Supplementary files**


| | Page No. |
|---|---|
| Supplementary table 1: Details of usual care by site | 2 |
| Supplementary table 2 Intervention delivery reported by Infant Feeding Helpers, by subgroups | 12 |
| Supplementary table 3 Any breastfeeding at 8-weeks: pre-specified subgroup analyses | 13 |
| Supplementary table 4 Any breastfeeding at 8-weeks: Further sensitivity analyses | 15 |
| Supplementary table 5 Self-reported formula feeding practices at 8 and 16-weeks | 16 |
| Supplementary table 6 Self-reported Maternal and infant health care utilisation at 8 and 16 weeks post birth | 17 |
| Supplementary table 7 Self-reported maternal use of support for infant feeding at 8 and 16 weeks post birth | 18 |
| Supplementary table 8 Sensitivity analysis to assess the impact of outliers of the GAD-7 score, EQ-5D-5L and MOS scores at 8 and 16 weeks | 20 |
| Supplementary figure 1 Any breastfeeding at 8 weeks post birth - Tipping point analysis: Scenario A - Risk ratio and 95% CI by event rate | 22 |
| Supplementary figure 2 Any breastfeeding at 8 weeks post birth - Tipping point analysis: Scenario B - Risk ratio and 95% CI by event rate | 24 |
| Supplementary file: Statistical analysis plan | 26 |
| Supplementary file: Statistical analysis code | 75 |

**Supplementary table 1 Details of usual care by site**

| Site | Geography | | | Maternity service - trust/health board (N)[1]; births/year (N) | BFI accreditation | Breastfeed-ing initiation (baseline) | Infant feeding team (baseline) | Infant feeding support antenatal | Infant feeding support postnatal | Peer support service (baseline) (N volunteer/paid PSs; type of provider; coordinator info) | Key changes to usual care over course of trial |
|---|---|---|---|---|---|---|---|---|---|---|---|
| | Urban/rural classification | % areas in 10% most deprived[2] | % White British (2021 census)[3] | | | | | | | | |
| 1 | Predominantly urban | 36.3% LSOAs in most deprived 10% in England (IMD, 2019). | 57% | 1 trust; 1900 | Maternity trust: gold; Community: gold | 76% | Infant feeding team – 1 FT, 1PT | Infant feeding discussed at booking, during 25-34 weeks, and by 34 weeks' gestation. | Day 1 after discharge<br>Day 3<br>Day 5 - at clinic/ birth centre/ home;<br>Day 5 - 10 needs based;<br>Day 10 - discharge - or later (needs based) | PS on postnatal wards. No breastfeeding groups running at baseline. | Plans in 2024 to commission community peer support. |
| 2 | Predominantly rural | Around 6% of data zones in 20% most deprived (SIMD 2020) | 94% (White Scottish /Other White British - rounded) (Census 2022) | 1 health board; 853 (2021/22) | Maternity Gold award reaccreditation in Dec 21, reaccredited Dec 23.<br><br>HV and Family Nurse Partnership | 72% | Infant Feeding Lead (PT); Infant Feeding Advisor (PT); Health Improvement | Advice and information from CMWs from 28 weeks gestation. Feeding covered in antenatal classes | Midwife or Maternity Care Assistant visits 2 or 3 times in first 10 days.<br><br>Discharged to health visitor at 10 days | 18 PS, all volunteers, NHS, coordinated by Infant Feeding Advisor and Health Improvement Specialist Advice and support via | Breastfeeding groups started to return April 2022.<br><br>Antenatal classes from June 2022 |

| | | | | | | | | | | | |
|---|---|---|---|---|---|---|---|---|---|---|---|
| | | | | | reaccredited May 24 | | Specialist (PT) | 2 hour online feeding advice and information session with IFT<br><br>HV AN contact around 32/34 weeks | Weekly online breastfeeding group led by IFT | proactive phone-calls for breastfeeding women (opt-in) ward visits for all women and breastfeeding groups | |
| 3 | Predominantly urban | 15% of population lived in the most deprived 10% of areas in England in 2019 | 72% | 2 trusts; 11000 | Maternity trusts 1 and 2: full accreditation;<br><br>Children's centres full accreditation | Trust 1 areas 56.8% Trust 2 areas 78.7% | Trust 2: 1x FT midwife plus 1xPT midwife carry out in-service training, BFI, manages tongue tie service and breastmilk bank.<br><br>Trust 1: 2xPT infant feeding midwives | Trust 1: Women referred for info to pregnancy app and website. Start for Life and BFI leaflets. Trust 2: Feeding information from MWs provide at every visit. | PN support mixture of home and clinic-based visits with MW team day 1 (home), 3 (home or clinic with MSW/MW), 5 (clinic), 14. Discharged at 14 days. MSW 1 day IF training.<br><br>Page in notes for feeding assessment, updated day 3,5 and discharge. Action card pathway for feeding problems with | 5 PT paid peer supporters (council funded, mainly covering lower breastfeeding areas) and volunteers.<br><br>Usually run breast feeding clinics in many different areas but these were put on hold at the beginning of the study and gradually came back into use throughout the study. | Before the end of the study council requested no further ABA feed recruitment in 3 key target areas due to starting proactive peer support (including AN contact). |

| | | | | | | | | | | | |
|---|---|---|---|---|---|---|---|---|---|---|---|
| | | | | | | | | | lead midwife, specialist clinics for feeding and tongue tie division. | | |
| 4 | Urban with significant rural | 1% LSOA in most deprived 10% in England (IMD, 2019). | 90% | Hospital 1, 3180, Hospital 2 ND due to intrapartum care suspended | Hospital1 reaccredited 2022, hospital 2 lapsed. | Hospital 1 70%, hospital 2 ND in 2021 (intrapartum care suspended) | Hospital 1: Infant Feeding Lead plus 2 support workers; hospital 2: 1 infant Feeding Lead 0.4TFE | Hospital 1: feeding discussed at booking appointment (8-12 weeks gestation), 16 weeks 36 weeks during birth plan discussion. Infant Feeding Team run 90 min sessions on infant feeding. Hospital 2: Midwives discuss antenatally at booking / 16 weeks / 28 weeks. Antenatal information pack includes section on Infant Feeding | Hospital 1: Midwife home visit day following discharge, MSW day 5 visit. Hospital 2: midwife home visit day following discharge. Day 5 visit. Day 10 midwife home visit. Additional visits as required. | 10 volunteer peer supporters recruited for the trial | Hospital 2 re-opened for intrapartum care in 2023. No. Peer supporters increased and more support at breastfeeding groups at children's centres. |

| | | | | | | | | | | | |
|---|---|---|---|---|---|---|---|---|---|---|---|
| 5 | Urban/rural mix | | 93 to 95% | 1 health board; 5500 | Health Board: full accreditation | 52% | 2 x PT Infant Feeding Leads. | Feeding discussion not limited to specified time but preference between 30-32 weeks. Antenatal education suspended during pandemic.<br><br>HVs make AN contact. | Face to face contact on days 1, 3 and 5 and discharge to HVs at 10-12 days or longer depending on need | Numbers unclear but all volunteers. Voluntary service provider. Mainly support through social media. | July 2023: 7 new peer supporters trained by Association of Breastfeeding Mothers (ABM) join to provide support in hospital |
| 6 | Predominantly urban | 10.9% LSOAs in most deprived 10% in England | 82% | 1 trust; 3500 | Maternity trust: full accreditation | 62% | 2 x specialist midwives and 2 x community breastfeeding support workers | Breastfeeding workshops and visits/conversations.<br><br>Health visitor antenatal contact at 28w. | Midwife visits day after going home, day 5, day 10-12.<br><br>Health visitor contact at 10-14days and 6-8w. | 30 volunteers (cover larger area); third sector provider. | |
| 7 | Predominantly rural | Around 10% of data zones in 20% most deprived (SIMD 2020) | 95% (White Scottish /Other White British - rounded) (Census 2022) | 1 health board: 1081 (2021/22) | Maternity reaccreditation in August 23 (failed reaccreditation in Nov 22);<br>HV service and FNP achieved Gold Award in 2022 | 63.6% | Infant Feeding Coordinator and 2 x Breastfeeding Support Coordinators (PT) | | | | Paid peer supporter visiting wards (PT) for part of trial period. |

| | | | | | | | | | | | |
|---|---|---|---|---|---|---|---|---|---|---|---|
| | | | | | | | Band 6 Nurse (PT) | | | | |
| 8 | Predominantly urban | LA1: >30% LA2: >20% of data zones in 20% most deprived (SIMD 2020) | 92% to 93% (Census 2022) | 1 health board: 4214 (2021/22) | Maternity hospital: gold; Community: gold | 50% | | | | | |
| 9 | Urban with significant rural | 2.75% LSOS in most deprived 10% in England | 90% | 2 trusts; 11000 Approx 2000 from LA | Maternity trusts 1 and 2: full accreditation | Areas used 60% | See Site 3 Trust 1 | See Site 3 Trust 1 | See Site 3 Trust 1 | Paid peer support coordinator (also responsible for other children's services) and 11 volunteer peer supporters. Service funded and provided by council. Volunteers run BF groups in community centres. | |
| 10 | Predominantly urban | 17% LSOAs in most deprived 10% in England; in the 30% most-deprived LA nationally | 90% | 1 trust; 3500 | Maternity trust: stage 2; Children's centres Stage 2; HV: full accreditation 2016, reassessment due; | 74% | Infant Feeding Lead 0.8FTE, spends 1 day in community and the rest in hospital | IF leaflet at booking, 34 weeks talk and Bump to Baby leaflet. MW encouraged to talk about IF at routine visits from 34 weeks. | PN discharge around 14 days but can be 10 to 28 days - kept on especially if feeding problems. Seen at day 3 and 5 at home or clinic by MSW. Not seen | NHS funded, social enterprise provider. 3 volunteer peer supporters at baseline with plans to get more. Before COVID peer supporters went into | None. |

| | | | | | | | | | | | | |
|---|---|---|---|---|---|---|---|---|---|---|---|---|
| | | | | | | | | All those wanting to BF given AN expressing packs. ABM leaflet given out.<br><br>HVs do Antenatal visits and send a digital information pack. | by MW until discharge to HV between 14-28 days | maternity hospital, paused until late 2022. Also meet women at MSW PN visits (3&5 days) and have a Facebook page. | |
| 11 | Predominantly rural | 1.3% of LSOAs in most deprived 10% of LSOAs in Wales (WIMD, 2019). | 95% | 1 health board (community care only); | Health Board and HV services: reassessment overdue | 74% | Infant feeding coordinator. MW and HV IF champions - MWs/HVs will go to them initially and then IF Coordinator for advice | Expectation of 3 AN conversations to be documented in notes, including 16 weeks and 2 hr session at 36 weeks. Solihull AN prep classes offered to all women and families includes 2hr session on IF.<br><br>HV AN visits only for vulnerable | Postnatal visit day after discharge/home birth, 3 days and 5 days: feeding assessment including BF observation. MWs are on call 24/7 for BF support in woman's homes. 5 to 6 PN visits, primips definite 4 visits. Discharge to HV 10-14 days | Approx 30 peer support volunteers from independent voluntary organisations (becoming part of health board). PS mainly attend BF groups (x8) but do also support 1-1 and virtually. | Voluntary peer supporter services became part of health board. |

| | | | | | | | | | | | |
|---|---|---|---|---|---|---|---|---|---|---|---|
| | | | | | | | | women (10%). | | | |
| 12 | Predominantly urban | 20.1% LSOAs in most deprived 10% in England (IMD, 2019). | 92% | 2 trusts; 9800 | Maternity trust 1: Stage 2; Trust 2: reassessment overdue; community: reassessment overdue | Hospital1 61%; Hospital2 65% | Hospital 1: Infant feeding adviser + 2 support workers. Hospital 2: 1 FT infant feeding coordinator | Hospital 1: antenatal feeding discussions, feeding workshop from 28+ weeks, telephone helpline, colostrum harvesting packs. Hospital 2: antenatal feeding discussions | Hospital 1 Routine visits first day after discharge date, days 5 & 10. Hospital 2, Routine visits first day after discharge date, days 5 & 10. additional visit day 3 if feeding issues. | Approx 28 volunteer peer supporters and 2 x volunteer coordinators | New specialist infant feeding clinics opened 2022 |
| 13 | Predominantly urban | No LSOS in most deprived 10% in England | 86% | Service provided by Site 3-Trust 2 | Maternity trust 1: full accreditation; Maternity trust 2: full accreditation; Community: full accreditation; Children's Centres: intent registered | Areas used 78.7% | See Site 3-Trust 2 | See Site 3-Trust 2 | See Site 3-Trust 2 | 2 part time paid peer support coordinators and 7 volunteers. Council funded service run by charity. Peer supporters run BF groups. | |
| 14 | Predominantly urban | 13% LSOA in Health Board area are in the most deprived 10% of Wales | 88 to 95% | 1 health board; 3500 | Maternity hospital: stage 1; HV: reassessment overdue | 64% | Fragmented team. 3 MSWs FT in community to support breastfeeding, 3 | MWs and MSWs provide BF information which is documented in handheld notes. | Primips seen day after discharge and day 4 at home. Discharged to HV 10-14 days. Use Unicef feeding assessment | 6 volunteer peer supporters provided by Health Board. Work on wards and in BF groups. | From July 2023, IF coordinator time increased to 1xFTE<br><br>Antenatal breastfeedi |

| | | | | | | | | | | | |
|---|---|---|---|---|---|---|---|---|---|---|---|
| | | (WIMD 2019). | | | | | MSWs PT in the hospital but sometimes do care other than breastfeeding. 1 midwife 0.3FTE breastfeeding support and 1 0.5 WTE. Not a team - covered by different managers and can sometimes all be on AL at the same time. | Breastfeeding workshop previously provided by MSWs but suspended due to the pandemic, not restarted by Nov 2021. Jan 2024, MWs discuss IF at 31 weeks; all women to be invited to Virtual IF workshop. HVs - no AN contact apart from women with Flying Start. | forms and plan put in place. | | ng education via videoconference commenced by Infant Feeding coordinator in May 2023 |
| 15 | Predominantly urban | 20.9% LSOAs in most deprived 10% in England (IMD, 2019). | 55% | 1 trust; 5000 | Maternity trust and community: reassessment overdue | 69% | 2 PT midwives and 1.4 full time infant feeding support workers | Discussion at least 3 times antenatally. Maternity app and trust website.<br><br>HV AN contact. | Computerised feeding assessment tool used during each shift while mother and baby in hospital and each postnatal contact. Home visit by midwife day 1 | 30 volunteers; 3 paid; hospital trust; dedicated PS coordinator<br><br>Hospital trust provider. | Peer Support Coordinator left role following Training the Trainers, prior to the trial starting, and new Coordinator appointed |

| | | | | | | | | | | | |
|---|---|---|---|---|---|---|---|---|---|---|---|
| | | | | | | | | | after leaving hospital and home visit or clinic appointment at day 3, 5 and 10 (day of discharge to health visiting services) | | Nov 2023: 5 new paid roles added to Infant Feeding Team (Universal Service Practitioners offering 1 to 1 feeding support in breastfeeding groups, via home visits, telephone or video support) |
| 16 | Predominantly rural | 5.2% LSOS in most deprived 10% in England | 94% | 1 trust; 3800 | Maternity trust: full accreditation, due for reassessment; LA Gold award; | 77% | FTE band 7 and band 6 (both TT practitioners). Also 0.2 band 3 HCA.<br>LNU: WTE band 4, 15 hours band 6. | 2hr online sessions feeding specific and AN conversation.<br><br>No HV AN contact. | PN contacts at day 1, 5 and 10 (unknown whether clinic or home). Discharge to HV at 10 days generally. | 7 paid Infant Feeding Peer Support Workers (IFPSWs). 54 volunteers. Paid PS coordinators, 60 hours.<br><br>Funded by Start for Life, provided by council.<br><br>Peer supporters in hospitals and BF groups. | Number of volunteer peer supporters increased from 54 to 76, and paid peer supporters from 7 to 20 (including working in hospitals). Number of BF groups increased. |

| | | | | | | | | | | | |
|---|---|---|---|---|---|---|---|---|---|---|---|
| 17 | Predominantly urban | 15.7% of LSOAs ln 10% most deprived areas of England. 39th most income deprived LA in England. | 92% | 1 trust; 1100 | Maternity: full accreditation; Community: Gold award; Children's Centres: Stage 2 | 76% | IF leads for HV and MW. MSW working in IF role (30hrs). BFI Practice Team Lead employed by Action for Children manages baby friendly. | Conversation with MW antenatally. AN classes about breastfeeding<br><br>HVs AN universal visit | Standard minimum maternity postnatal care is first day home visit, day 5 visit, discharge to HV appointment day 10-14. Feeding assessments but no plan. | 18 active peer supporters. Charity provider, Family Hub funding.<br><br>Volunteering at feeding groups or groups at children's centres + community group, plus one volunteers at hospital. | 8 more active PSs in 2024. All volunteers. |

Abbreviations: AN=Antenatal, BFI=Baby Friendly Initiative, HV=Health Visitor, LA=Local Authority, LSOS=Lower Super Output Areas, MSW=Maternity Support Worker, MW=Midwife, PN=Postnatal.

[1]Number of births for the Trust/Board not necessarily the number of births at the site.

[2]Deprivation data from: https://www.gov.uk/government/statistics/english-indices-of-deprivation-2019.

[3]Census data: https://www.ons.gov.uk/census/maps/choropleth/identity/ethnic-group/ethnic-group-tb-20b/white-english-welsh-scottish-northern-irish-or-british?lad=E08000025

**Supplementary table 2 Intervention delivery reported by Infant Feeding Helpers, by subgroups**

| | **n** | **Main meeting** n (%) | **At least one antenatal** n (%) | **At least one postnatal** n (%) | **PN contact Low (<4)** n (%) | **PN contact Medium (4-8)** n (%) | **PN contact High (>8)** n (%) | **Contacted by IFH within 48 hours of birth** n (%) | **Number of total meetings** Median (IQR) | **Number of postnatal meetings** Median (IQR) |
|---|---|---|---|---|---|---|---|---|---|---|
| **Age group** | | | | | | | | | | |
| Age <25 | 118 | 80 (67.8) | 101 (85.6) | 104 (88.1) | 31 (26.3%) | 19 (16.1%) | 68 (57.6%) | 70 (59.3%) | 14.5 (5-23) | 11.0 (2-17) |
| Age 25+ | 1139 | 953 (83.7) | 1017 (89.3) | 1064 (93.4) | 158 (13.9%) | 110 (9.7%) | 871 (76.5%) | 840 (73.7%) | 21.0 (13-25) | 16.0 (9-21) |
| **Educational level** | | | | | | | | | | |
| Below degree | 379 | 289 (76.3) | 333 (87.9) | 347 (91.6) | 72 (19.0%) | 48 (12.7%) | 259 (68.3%) | 253 (66.8%) | 19.0 (10-24) | 15.0 (6-0) |
| Degree/equivalent | 868 | 737 (84.9) | 775 (89.3) | 815 (93.9) | 113 (13.0%) | 80 (9.2%0 | 675 (77.8%) | 652 (75.1%) | 21.0 (13-25) | 16.5 (10-21) |
| **Deprivation** | | | | | | | | | | |
| IMD 1 (most deprived) | 247 | 189 (76.5) | 206 (83.4) | 226 (91.5) | 49 (19.8%) | 31 (12.6%) | 167 (67.6%) | 160 (64.8%) | 18.0 (9-23) | 14.0 (6-19) |
| IMD 2 | 255 | 207 (81.2) | 230 (90.2) | 238 (93.3) | 42 (16.5%) | 34 (13.3%) | 179 (70.2%) | 189 (74.1%) | 20.0 (10-25) | 16.0 (6-19) |
| IMD 3 | 262 | 220 (84.0) | 241 (92.0) | 240 (91.6) | 37 (14.1%) | 18 (6.9%) | 207 (79.0%) | 192 (73.3%) | 20.0 (13-25) | 16.0 (10-20) |
| IMD 4 | 288 | 238 (81.6) | 254 (88.2) | 271 (94.1) | 37 (12.8%) | 32 (11.1%) | 219 (76.0%) | 215 (74.7%) | 20.0 (13-25) | 17.0 (9-21) |
| IMD 5 (least deprived) | 201 | 177 (88.1) | 183 (91.0) | 189 (94.0) | 22 (10.9%) | 14 (7.0%) | 165 (82.1%) | 151 (75.1%) | 23.0 (16-29) | 18.0 (12-22) |
| **Feeding intention** | | | | | | | | | | |
| Mainly/only formula | 91 | 61 (67.0%) | 71 (78.0) | 77 (84.6) | 26 (28.6%) | 16 (17.6%) | 49 (53.8%) | 55 (60.4%) | 14.0 (5-22) | 10.0 (2-18) |
| Only/mainly/half breastmilk | 1162 | 969 (83.4%) | 1045 (89.9) | 1088 (93.6) | 162 (13.9%) | 113 (9.7%) | 887 (76.3%) | 853 (73.4%) | 20.0 (13-25) | 16.0 (9-21) |

Abbreviations: PN=Postnatal, IMD=Index of Multiple Deprivation, IQR=Interquartile Range, n=Number of observations.

**Supplementary table 3 Any breastfeeding at 8-weeks: pre-specified subgroup analyses**

| | ABA-Feed intervention (N=1458) n/n (%) | Usual Care (N=1017) n/n (%) | Interaction p-value | Risk ratio[1] (95% CI) | Ratio[2] (95% CI) |
|---|---|---|---|---|---|
| **Woman's age** | | | P=0.71 | | |
| <25 years | 66/137 (48.2) | 44/96 (45.8) | | 1.06 (0.81 to 1.39) | 1.05 (0.80 to 1.39)[3] |
| ≥25 years | 947/1315 (72.0) | 654/919 (71.2) | | 1.01 (0.96 to 1.06) | REF |
| **Feeding intentions** | | | P=0.64 | | |
| Breast milk only | 570/690 (82.6) | 418/509 (82.1) | | 1.01 (0.96 to 1.07) | REF |
| Mainly breast milk | 333/465 (71.6) | 220/300 (73.3) | | 0.98 (0.89 to 1.07) | 0.96 (0.87 to 1.07)[5] |
| Half and half breast and formula milk | 96/186 (51.6) | 56/133 (42.1) | | 1.21 (0.94 to 1.56) | 1.20 (0.93 to 1.53)[6] |
| Mainly formula | 5/38 (13.2) | 4/30 (13.3) | | 0.97 (0.28 to 3.30) | 0.96 (0.28 to 3.26)[7] |
| Formula milk only | 4/68 (5.9) | 0/43 (0) | | Not estimable | Not estimable[8] |
| **Mother's education[4]** | | | P=0.26 | | |
| No formal qualification | 1/5 (20.0) | 3/4 (75.0) | | 0.25 (0.04 to 1.57) | 0.25 (0.04 to 1.58)[9] |
| GCSE, Standard Grade, National 5 or equivalent | 61/144 (42.4) | 36/101 (35.6) | | 1.17 (0.85 to 1.62) | 1.18 (0.85 to 1.63)[10] |
| A-level/AS level, Highers or equivalent | 158/251 (63.0) | 95/165 (57.6) | | 1.08 (0.92 to 1.27) | 1.09 (0.92 to 1.29)[11] |
| Degree level or above | 766/1007 (76.1) | 545/711 (76.7) | | 1.00 (0.95 to 1.05) | REF |
| **Index of Multiple Deprivation (IMD)** | | | P=0.64 | | |
| 1st quintile group (most deprived)) | 195/283 (68.9) | 124/204 (60.8) | | 1.07 (0.95 to 1.21) | 1.10 (0.93 to 1.30)[12] |
| 2nd quintile group | 199/297 (67.0) | 137/209 (65.6) | | 1.05 (0.93 to 1.17) | 1.07 (0.91 to 1.26)[13] |
| 3rd quintile group | 213/303 (70.3) | 140/195 (71.8) | | 0.96 (0.87 to 1.06) | 0.99 (0.85 to 1.15)[14] |
| 4th quintile group | 246/339 (72.6) | 168/231 (72.7) | | 1.01 (0.92 to 1.10) | 1.03 (0.89 to 1.20)[15] |
| 5th quintile group (least deprived)) | 158/224 (70.5) | 124/168 (73.8) | | 0.98 (0.87 to 1.10) | REF |
| **Relationship status** | | | P=0.86 | | |
| Married or in a registered civil partnership, or living together | 958/1362 (70.3) | 658/941 (69.9) | | 1.00 (0.95 to 1.05) | 0.97 (0.72 to 1.31)[16] |

| | | | | | |
|---|---|---|---|---|---|
| Single, or widowed, divorced or separated | 38/69 (55.1) | 32/60 (53.3) | | 1.03 (0.76 to 1.38) | REF |

Abbreviations: CI=Confidence Interval, IMD=Index of Multiple Deprivation, n=Number of observations, REF=Referenced.
[1]Adjusted risk ratio for minimisation variables (mother's age as a categorical fixed effect and site as categorical random effect; IFH was removed due to convergence issues) and the interaction of treatment by the subgroup variable: mother's age, a value>1 favours planned ABA-Feed intervention.
[2]Ratio of subgroup effects.
[3]Woman's age (<25 years) vs woman's age (≥25 years).
[4]In this subgroup analysis site was removed from the model because of convergence issues.
[5]Pre-specified as feeding intentions (Mainly breast milk) vs Pre-specified as feeding intentions (Breast milk only).
[6]Pre-specified as feeding intentions (Half and half breast and formula milk) vs Pre-specified as feeding intentions (Breast milk only).
[7]Pre-specified as feeding intentions (Mainly formula) vs Pre-specified as feeding intentions (Breast milk only).
[8]Pre-specified as feeding intentions (Formula milk only) vs Pre-specified as feeding intentions (Breast milk only).
[9]Mother's education (No formal qualification) vs Mother's education (Degree level or above).
[10]Mother's education (GCSE, Standard Grade, National 5 or equivalent) vs Mother's education (Degree level or above).
[11]Mother's education (A-level/AS level or equivalent) vs Mother's education (Degree level or above).
[12]IMD (1st quintile group [most deprived]) vs IMD (5th quintile group [least deprived]).
[13]IMD (2nd quintile group) vs IMD (5th quintile [least deprived]).
[14]IMD (3rd quintile group) vs IMD (5th quintile group [least deprived]).
[15]IMD (4th quintile group) vs IMD (5th quintile group [least deprived]).
[16]Relationship status (Married or in a registered civil partnership, or Living together) vs Relationship status (Single, or widowed, divorced or separated).

**Supplementary table 4 Any breastfeeding at 8-weeks: Further sensitivity analyses**

| | Planned ABA-Feed intervention (N=1458) n (%) | Usual Care (N=1017) n (%) | Risk ratio[1] (95% CI; p-value) | Risk difference[2] (95% CI; p-value) |
|---|---|---|---|---|
| Complete case analysis[3] | | | | |
| Yes | 1013 (70.7) | 698 (69.2) | 1.01 (0.97 to 1.06; 0.61) | 0.01 (-0.02 to 0.05; 0.53) |
| No | 420 (29.3) | 311 (30.8) | | |
| Missing due to pregnancy loss | 6 | 2 | | |
| Missing | 19 | 6 | | |
| Worst-case scenario analysis[4] | | | | |
| Yes | 1013 (69.8) | 704 (69.4) | 1.00 (0.95 to 1.05; 0.97) | 0.002 (-0.03 to 0.04; 0.92) |
| No | 439 (30.2) | 311 (30.6) | | |
| Missing due to pregnancy loss | 6 | 2 | | |
| Best-case scenario analysis[5] | | | | |
| Yes | 1032 (71.1) | 698 (68.8) | 1.02 (0.97 to 1.07; 0.38) | 0.02 (-0.02 to 0.05; 0.30) |
| No | 420 (28.9) | 317 (31.2) | | |
| Missing due to pregnancy loss | 6 | 2 | | |

Abbreviation: CI=Confidence Interval, n=Number of observations.

[1]Adjusted risk ratio for minimisation variables (mother's age as a categorical fixed effect and site as categorical random effect; IFH was removed due to convergence issues), a value>1 favours planned ABA-feed intervention.

[2]Adjusted risk difference for minimisation variables (mother's age as a categorical fixed effect and site as categorical random effect; IFH was removed due to convergence issues), a value>0 favours planned ABA-feed intervention.

[3]by excluding from the primary analysis participants with missing the primary outcome data.

[4]in which those with missing data in the planned ABA-feed intervention group are assumed to be formula feeding, and those with missing data in the usual care group are assumed to be breastfeeding.

[5]in which those with missing data in the planned ABA-feed intervention group are assumed to be breastfeeding, and those with missing data in the usual care group are assumed to be formula feeding.

**Supplementary table 5 Self-reported formula feeding practices at 8 and 16-weeks**

| **Recommended practices** | **8 weeks** | | **16 weeks** | |
|---|---|---|---|---|
| | **ABA-feed (N=1458)** | **Usual care (N=1017)** | **ABA-feed (N=1458)** | **Usual care (N=1017)** |
| | n/n (%) | n/n (%) | n/n (%) | n/n (%) |
| Making one feed at a time | 506/575 (88.0) | 355/405 (87.7) | 517/597 (86.6) | 370/414 (89.4) |
| Correct water temperature | 244/537 (45.4) | 184/373 (49.3) | 233/562 (41.5) | 186/387 (48.1) |
| Adding formula powder after water | 241/270 (89.3) | 181/206 (87.9) | 237/262 (90.5) | 197/216 (91.2) |
| Making up formula when needed when out of the home | 186/284 (65.5) | 122/184 (66.3) | 232/351 (66.1) | 155/236 (65.7) |
| Keeping pre-prepared milk chilled when out of the home[1] | 33/98 (33.7) | 31/62 (50.0) | 60/117 (51.3) | 37/80 (46.3) |
| Making formula with hot water when out of the home | 161/183 (88.0) | 103/120 (85.8) | 204/232 (87.9) | 139/151 (92.1) |
| Sterilising bottles using recommended methods | 344/383 (89.8) | 254/274 (92.7) | 519/583 (89.0) | 365/400 (91.3) |

Abbreviation: n=Number of observations.

[1]Denominator was respondents who reported that if they had to feed their baby outside the home they prepared the formula before leaving home

**Supplementary table 6 Self-reported Maternal and infant health care utilisation at 8 and 16 weeks post birth**

| | ABA-Feed intervention (N=1458) | Usual Care (N=1017) | ABA-Feed intervention (N=1458) | Usual Care (N=1017) |
|---|---|---|---|---|
| | 0-8 weeks | | 8-16 weeks | |
| **INFANT** | | | | |
| Number attended A&E for feeding related cause, n/n (%) | 82/1253 (6.5) | 46/854 (5.4) | 36/1171 (3.1) | 27/823 (3.3) |
| Number admitted to hospital for any reason, n/n (%) | 192/1251 (15.4) | 126/854 (14.8) | 77/1165 (6.6) | 54/821 (6.6) |
| Number admitted to hospital for feeding related problem, n/n (%) | 143/1251 (11.4) | 99/854 (11.6) | 30/1165 (2.6) | 21/821 (2.6) |
| GP/family doctor consultations, mean (n, sd) | 1.1 (1152,1.3) | 0.9 (795, 1.1) | 0.8 (1042, 1.1) | 0.8 (737, 1.2) |
| Midwife consultations, mean (n, sd) | 0.8 (1074, 1.5) | 0.9 (748, 1.6) | N/A | N/A |
| Health visitor consultations, mean (n, sd) | 0.9 (1100, 1.3) | 0.9 (758,1.4) | 0.5 (966, 0.9) | 0.6 (679, 1.0) |
| Practice nurse consultations, mean (n, sd) | N/A | N/A | 0.1 (917, 0.3) | 0.1 (638, 0.4) |
| **WOMEN** | | | | |
| Number attended A&E for problem related to breastfeeding, n/n (%) | 24/1254 (1.9) | 19/853 (2.2) | 12/1165 (1.0) | 8/820 (1.0) |
| Number admitted to hospital for any reason, n/n (%) | 100/1244 (8.0) | 65/843 (7.7) | 30/1161 (2.6) | 26/812 (3.2) |
| Hospital admissions for problem related to breastfeeding, n/n (%) | 12/100 (12.0) | 13/64 (20.3) | 3/29 (10.3) | 4/25 (16.0) |
| GP/family doctor consultations, mean (n, sd) | 0.3 (1071, 0.8) | 0.3 (735, 0.7) | 0.1 (968, 0.5) | 0.2 (679, 0.5) |
| Midwife consultations, mean (n, sd) | 0.4 (1045, 1.1) | 0.4 (726, 1.0) | N/A | N/A |
| Health visitor consultations, mean (n, sd) | 0.3 (1042, 0.8) | 0.3 (716, 0.9) | 0.1 (948, 0.4) | 0.1 (665, 0.5) |

Abbreviation: A&E=Accident & Emergency, CI=Confidence Interval, GP=General Practitioner, n=Number of observations, N/A=Not Applicable, sd=Standard Deviation.

**Supplementary table 7 Self-reported maternal use of support for infant feeding at 8 and 16 weeks post birth**

| | 0 to 8 weeks | | 9 to 16 weeks | |
|---|---|---|---|---|
| | **ABA-Feed (N=1458)** n (%) | **Usual Care (N=1017)** n (%) | **ABA-Feed (N=1458)** n (%) | **Usual Care (N=1017)** n (%) |
| **Midwives** | | | | |
| Not at all | 159 (12.7) | 95 (11.2) | 932 (80.1) | 631 (77.0) |
| Once | 203 (16.2) | 143 (16.8) | 77 (6.6) | 71 (8.7) |
| Twice | 284 (22.6) | 235 (27.7) | 59 (5.1) | 49 (6.0) |
| 3 – 5 times | 433 (34.5) | 259 (30.5) | 72 (6.2) | 54 (6.6) |
| > 5 times | 176 (14.0) | 117 (13.8) | 24 (2.0) | 14 (1.7) |
| Missing | 203 | 168 | 294 | 198 |
| **Health visitor** | | | | |
| Not at all | 125 (10.0) | 92 (10.9) | 728 (62.5) | 501 (61.6) |
| Once | 225 (18.0) | 137 (16.2) | 283 (24.3) | 194 (23.9) |
| Twice | 483 (38.5) | 345 (40.9) | 103 (8.8) | 73 (9.0) |
| 3 – 5 times | 365 (29.1) | 227 (26.9) | 45 (3.9) | 39 (4.8) |
| > 5 times | 55 (4.4) | 43 (5.1) | 6 (0.5) | 6 (0.7) |
| Missing | 205 | 173 | 293 | 204 |
| **General practitioner** | | | | |
| Not at all | 799 (63.7) | 569 (67.3) | 973 (83.8) | 666 (81.5) |
| Once | 308 (24.6) | 181 (21.4) | 119 (10.3) | 97 (11.9) |
| Twice | 97 (7.7) | 64 (7.6) | 47 (4.0) | 36 (4.4) |
| 3 – 5 times | 35 (2.8) | 29 (3.4) | 16 (1.4) | 18 (2.2) |
| > 5 times | 15 (1.2) | 2 (0.3) | 6 (0.5) | 0 (0) |
| Missing | 204 | 172 | 297 | 200 |
| **Practice nurse** | | | | |
| Not at all | 1118 (89.2) | 767 (90.8) | 1130 (97.0) | 778 (95.5) |
| Once | 93 (7.4) | 53 (6.3) | 22 (1.9) | 23 (2.8) |
| Twice | 21 (1.7) | 14 (1.7) | 7 (0.6) | 12 (1.5) |
| 3 – 5 times | 12 (1.0) | 8 (0.9) | 6 (0.5) | 1 (0.1) |
| > 5 times | 9 (0.7) | 3 (0.3) | 0 (0) | 1 (0.1) |
| Missing | 205 | 172 | 293 | 202 |
| **ABA-feed infant feeding helper** | | | | |
| Not at all | 106 (8.5) | 826 (97.5) | 834 (72.1) | 806 (98.7) |
| Once | 59 (4.7) | 13 (1.5) | 112 (9.7) | 9 (1.1) |
| Twice | 77 (6.2) | 3 (0.4) | 96 (8.3) | 1 (0.1) |
| 3 – 5 times | 211 (16.8) | 4 (0.5) | 77 (6.6) | 1 (0.1) |
| > 5 times | 800 (63.8) | 1 (0.1) | 38 (3.3) | 0 (0) |
| Missing | 205 | 170 | 301 | 200 |
| **Infant feeding counsellor or breastfeeding supporter[1]** | | | | |
| Not at all | 1059 (84.6) | 690 (81.5) | 938 (80.6) | 644 (78.8) |
| Once | 110 (8.8) | 95 (11.2) | 94 (8.1) | 99 (12.1) |
| Twice | 46 (3.7) | 35 (4.1) | 66 (5.7) | 39 (4.8) |
| 3 – 5 times | 24 (1.9) | 22 (2.6) | 51 (4.4) | 24 (2.9) |
| > 5 times | 13 (1.0) | 5 (0.6) | 14 (1.2) | 11 (1.4) |
| Missing | 206 | 170 | 295 | 200 |

| | | | | |
|---|---|---|---|---|
| **Breastfeeding telephone helpline** | | | | |
| Not at all | 1184 (94.3) | 775 (91.4) | 1,133 (97.3) | 788 (96.2) |
| Once | 52 (4.1) | 60 (7.1) | 27 (2.3) | 25 (3.1) |
| Twice | 16 (1.3) | 11 (1.3) | 2 (0.2) | 5 (0.6) |
| 3 – 5 times | 3 (0.3) | 2 (0.2) | 1 (0.1) | 1 (0.1) |
| > 5 times | 0 (0) | 0 (0) | 1 (0.1) | 0 (0) |
| Missing | 203 | 169 | 294 | 198 |
| **Friend(s)** | | | | |
| Not at all | 333 (26.7) | 290 (34.3) | 658 (56.5) | 477 (58.3) |
| Once | 98 (7.9) | 80 (9.5) | 149 (12.8) | 109 (13.3) |
| Twice | 160 (12.8) | 105 (12.4) | 134 (11.5) | 96 (11.7) |
| 3 – 5 times | 267 (21.4) | 150 (17.7) | 144 (12.3) | 70 (8.6) |
| > 5 times | 390 (31.2) | 221 (26.1) | 80 (6.9) | 66 (8.1) |
| Missing | 210 | 171 | 293 | 199 |
| **Family member(s)** | | | | |
| Not at all | 326 (26.1) | 280 (33.1) | 690 (59.3) | 498 (60.8) |
| Once | 91 (7.3) | 95 (11.2) | 132 (11.3) | 102 (12.4) |
| Twice | 127 (10.1) | 83 (9.8) | 119 (10.2) | 84 (10.3) |
| 3 – 5 times | 241 (19.3) | 149 (17.6) | 150 (12.9) | 82 (10.0) |
| > 5 times | 465 (37.2) | 240 (28.3) | 73 (6.3) | 53 (6.5) |
| Missing | 208 | 170 | 294 | 198 |
| **Internet support**[2] | | | | |
| Not at all | 845 (67.4) | 558 (65.8) | 870 (74.8) | 591 (72.2) |
| Once | 84 (6.7) | 76 (9.0) | 83 (7.1) | 75 (9.1) |
| Twice | 66 (5.2) | 50 (5.9) | 74 (6.4) | 50 (6.1) |
| 3 – 5 times | 90 (7.2) | 68 (8.0) | 71 (6.1) | 55 (6.7) |
| > 5 times | 169 (13.5) | 96 (11.3) | 65 (5.6) | 48 (5.9) |
| Missing | 204 | 169 | 295 | 198 |

Abbreviation: IFH=Infant Feeding Helper, n= Number of observations.

[1]Not ABA-feed infant feeding helper

[2]Posting to ask for support on internet/social media, NOT general browsing of web-based resources)

**Supplementary table 8 Sensitivity analysis to assess the impact of outliers of the GAD-7 score, EQ-5D-5L and MOS scores at 8 and 16 weeks**

Where extreme values were apparent and considered to be affecting the integrity of the analysis, the outlying responses were removed. The outliers were identified using the studentised residuals and the threshold of -3 / +3 values.

| | Planned ABA-Feed intervention (N=1458) | Usual Care (N=1017) | Mean difference[1] (95% CI; p-value) |
|---|---|---|---|
| **GAD-7 score[2]** | | | |
| Baseline | | | |
| Mean (n, sd) | 3.3 (1447, 3.5) | 3.5 (1005, 3.8) | N/A |
| Range: Min - Max | 0 - 21 | 0 - 21 | |
| Median | 2.0 | 2.0 | |
| IQR – [P25% , P75%] | [1.0, 5.0] | [1.0, 5.0] | |
| 8 weeks | | | |
| Mean (n, sd) | 4.2 (1218, 4.0) | 4.5 (825, 4.4) | -0.17 (-0.48 to 0.14; 0.28)[3] |
| Range: Min - Max | 0 – 21.0 | 0 – 21.0 | |
| Median | 3.0 | 4.0 | |
| IQR – [P25% , P75%] | [1.0, 6.0] | [1.0, 6.0] | |
| 16 weeks | | | |
| Mean (n, sd) | 4.0 (1133, 4.0) | 4.2 (789, 4.1) | -0.13 (-0.44 to 0.18; 0.42)[3] |
| Range: Min - Max | 0 – 21.0 | 0 – 21.0 | |
| Median | 3.0 | 3.0 | |
| IQR – [P25% , P75%] | [1.0, 6.0] | [1.0, 6.0] | |
| **EQ-5D-5L index score[4]** | | | |
| Baseline | | | |
| Mean (n, sd) | 0.85 (1449, 0.1) | 0.85 (1015, 0.1) | N/A |
| Range: Min - Max | -0.24 – 1.00 | 0.12 – 1.00 | |
| Median | 0.84 | 0.84 | |
| IQR – [P25% , P75%] | [0.77, 1.0] | [0.77, 1.0] | |
| 8 weeks | | | |
| Mean (n, sd) | 0.87 (1232, 0.1) | 0.87 (833, 0.1) | 0.004 (-0.01 to 0.01; 0.44)[5] |
| Range: Min - Max | 0.41 – 1.00 | 0.28 – 1.00 | |
| Median | 0.88 | 0.85 | |
| IQR – [P25% , P75%] | [0.77, 1.00] | [0.79, 1.00] | |
| 16 weeks | | | |
| Mean (n, sd) | 0.88 (1147, 0.1) | 0.88 (806, 0.1) | -0.001 (-0.01 to 0.01; 0.89)[5] |
| Range: Min - Max | 0.32 – 1.00 | 0.40 – 1.00 | |
| Median | 0.88 | 0.88 | |
| IQR – [P25% , P75%] | [0.77, 1.00] | [0.80, 1.00] | |

| **MOS score**[6] | | | |
|---|---|---|---|
| Baseline | | | |
| Mean (n, sd) | 89.3 (1457, 16.2) | 89.3 (1017, 15.9) | N/A |
| Range: Min - Max | 0 - 100 | 3.1 - 100 | |
| Median | 96.9 | 96.9 | |
| IQR – [P25% , P75%] | [84.4, 100.0] | [81.3, 100.0] | |
| 8 weeks | | | |
| Mean (n, sd) | 84.0 (1239, 18.2) | 81.8 (835, 19.7) | 2.03 (0.64 to 3.42; 0.004)[5] |
| Range: Min - Max | 6.3 – 100.0 | 9.4 – 100.0 | |
| Median | 90.6 | 87.5 | |
| IQR – [P25% , P75%] | [75.0 to 100.0] | [71.9, 100.0] | |
| 16 weeks | | | |
| Mean (n, sd) | 82.7 (1149, 20.3) | 82.4 (803, 20.3) | 0.66 (-1.85 to 2.16; 0.39)[5] |
| Range: Min - Max | 0 – 100.0 | 0 – 100.0 | |
| Median | 90.6 | 87.5 | |
| IQR – [P25% , P75%] | [75.0, 100.0] | [71.9, 100.0] | |

Abbreviations: CI=Confidence Interval, EQ-5D-5L=EuroQoL 5 dimensions 5-level, GAD-7= Generalised Anxiety Disorder Assessment, ITT=Intention-To-Treat, MOS= Medical Outcomes Study, n=Number of observations, $P_{25}$=25% Percentile, $P_{75}$=75% Percentile, sd=Standard Deviation.

[1]Mean difference adjusted for the minimisation variables (mother's age as a continuous and site as categorical random effect; IFH was removed due to convergence issues) and the baseline score,

[2]The total score GAD-7 ranges from 0 to 21 with 0 indicates lack of anxiety and 21 indicates the highest level of anxiety.

[3] A value<0 favours planned ABA-Feed intervention.

[4]The EQ-5D-5L index score was calculated using the mapping function developed by Van Hout et al. (2012) and the Crosswalk value sets for the UK; and it ranges from -0.594 to 1 with -0594 indicates unable to / extreme problems on all of the five dimensions and 1 indicates no problems on any of the five dimensions.

[5] A value>0 favours planned ABA-Feed intervention.

[6]The total mean score MOS consists of only the emotional / Informational dimension, which ranges from 0 to 100 with 0 indicates lower level of support and 100 indicates a higher level of support.

**Supplementary figure 1 Any breastfeeding at 8 weeks post birth - Tipping point analysis: Scenario A[1] - Risk ratio and 95% CI by event rate**

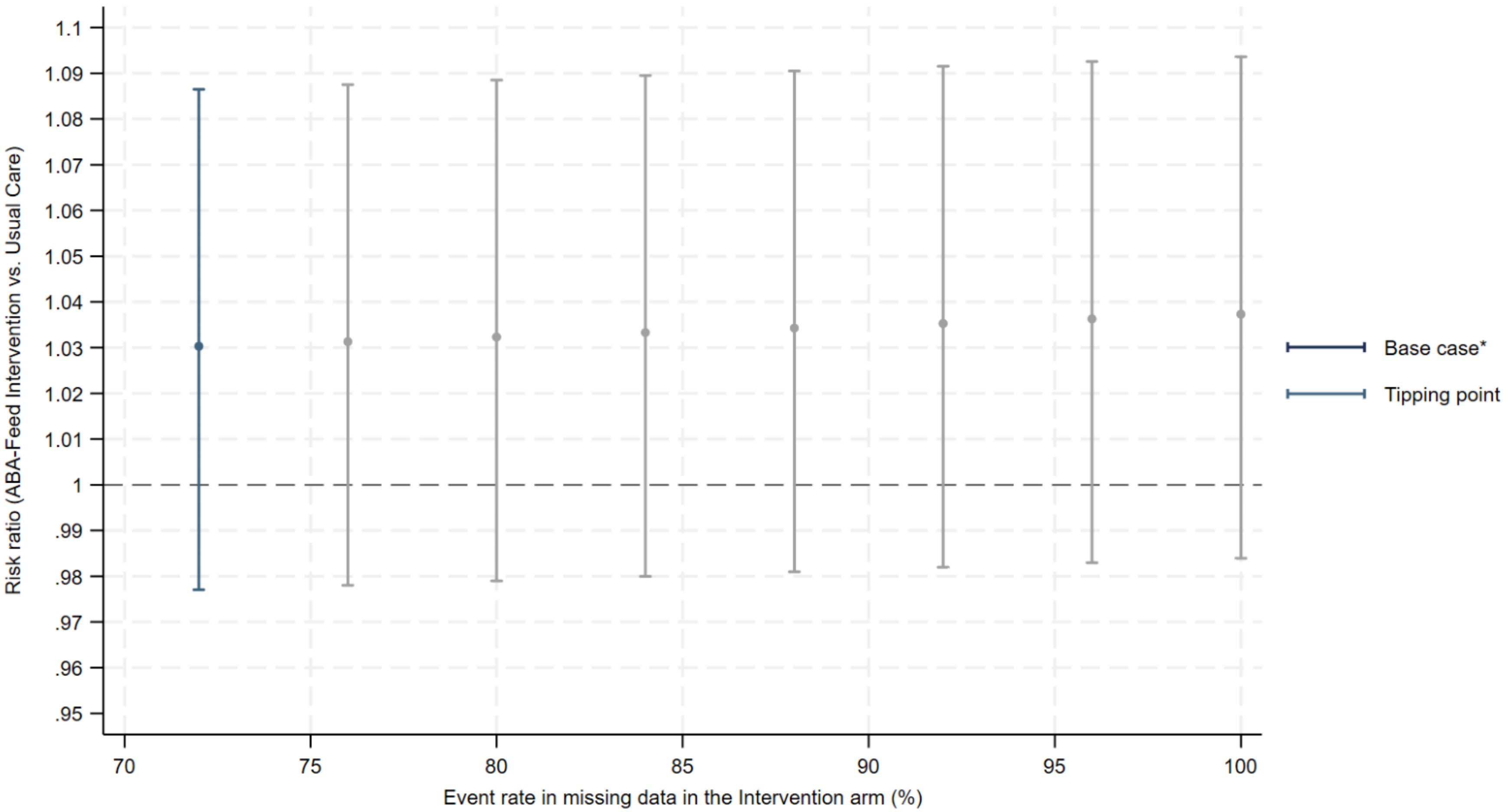


Note: Risk ratios >1 favour ABA-Feed Intervention.

*In the base case, the estimate is derived from the model where we assume the event rate in the missing data is equal to the event rate in the non-missing data in the intervention arm. All missing data in the control arm are assumed to be non-events.

[1]In scenario A, all missing responses in the usual care arm were imputed as non-events (No). In the ABA-Feed intervention arm, for the base case model, assumed that the event rate in the missing data was equal to that in the non-missing data of the ABA-Feed intervention arm. Consequently, 18 of the 25 missing primary outcome data in the ABA-Feed intervention arm were coded as events (Yes), while the remaining 7 were left as missing. Subsequently, the model was adjusted incrementally by imputing each of the remaining 7 missing primary outcome data as events (Yes), one at a time.

**Supplementary figure 2 Any breastfeeding at 8 weeks post birth - Tipping point analysis: Scenario B[1] - Risk ratio and 95% CI by event rate**

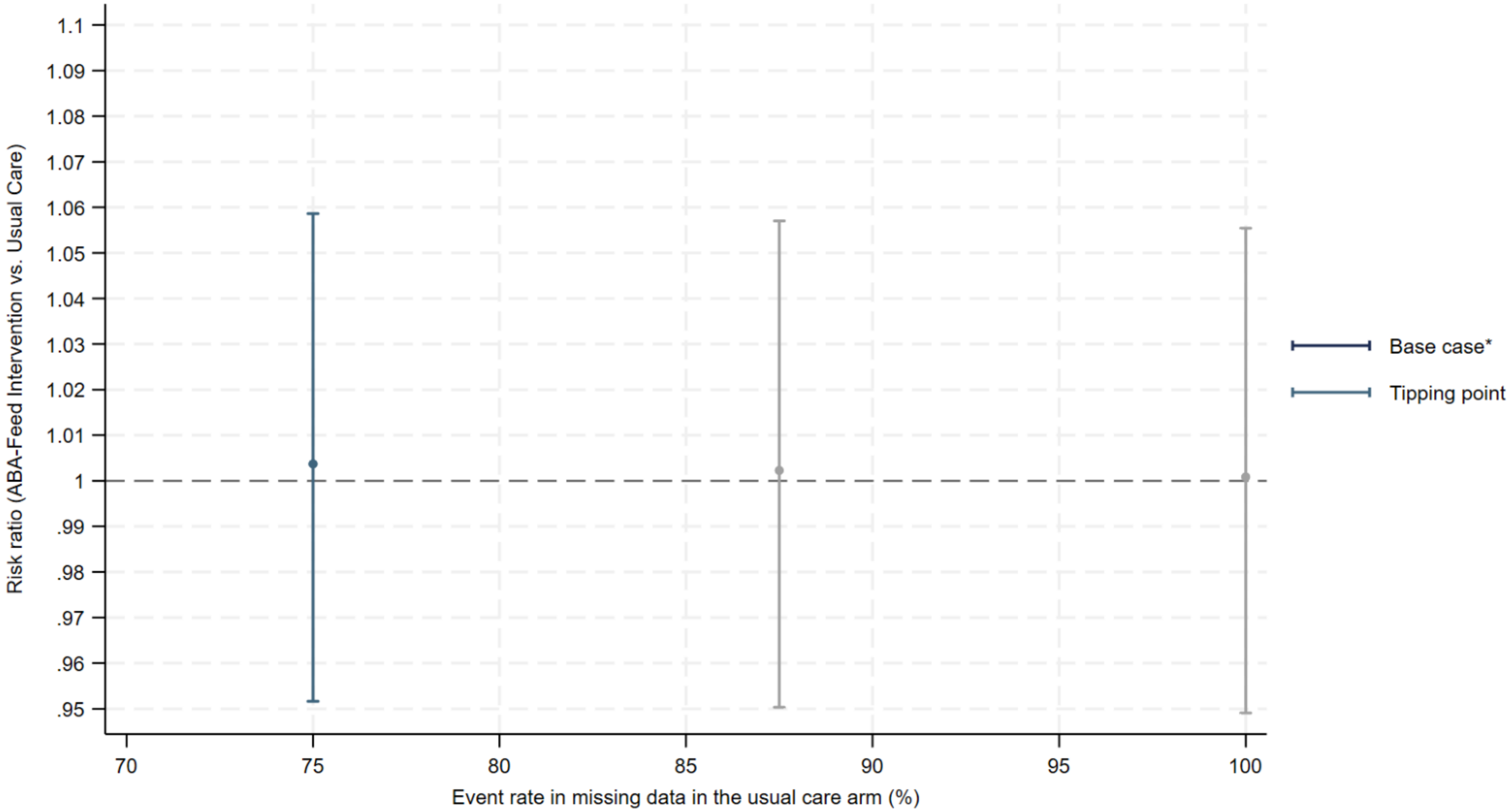


Note: Risk ratios >1 favour ABA-Feed Intervention.

*In the base case, the estimate is derived from the model where we assume the event rate in the missing data is equal to the event rate in the non-missing data in the control arm. All missing data in the intervention arm are assumed to be non-events.

[1]In scenario B, all missing responses in the ABA-Feed intervention arm were imputed as non-events (No). In the usual care arm, for the base case model, assumed that the event rate in the missing data was equal to that in the non-missing data of the usual care arm. Consequently, 6 of the 8 missing primary outcome data in the usual care arm were coded as events (Yes), while the remaining 2 were left as missing. Subsequently, the model was adjusted incrementally by imputing each of the remaining 2 missing primary outcome data as events (Yes), one at a time.

# The effectiveness and cost-effectiveness of Assets-based feeding help Before and After birth (ABA-feed) for improving breastfeeding initiation and continuation
# A multicentre randomised controlled trial with internal pilot

## The ABA-feed Trial

ABAfeed
Infant feeding help

Trial Registration: ISRCTN17395671

# Statistical Analysis Plan

| SAP Version Number | Protocol Version Number |
|---|---|
| 2.0 | 3.0 - 18 June 2021 |

<table>
<tr><td>Name of Author:</td><td>Eleni Gkini</td><td>Role:</td><td>Trial Statistician</td><td rowspan="2">Affiliation:</td><td rowspan="2">BCTU<br>University of Birmingham</td></tr>
<tr><td>Signature of Author:</td><td>Eleni Gkini<br>(e-signed)</td><td>Date:</td><td>17 Oct 2024</td></tr>
<tr><td>Name of Blind Reviewer:</td><td>Kelly Handley</td><td>Role:</td><td>Senior Statistician</td><td rowspan="2">Affiliation:</td><td rowspan="2">BCTU<br>University of Birmingham</td></tr>
<tr><td>Signature of Reviewer:</td><td>Kelly Handley<br>(e-signed)</td><td>Date:</td><td>21 Oct 2024</td></tr>
<tr><td>Name of Chief Investigator:</td><td>Prof Kate Jolly</td><td>Role:</td><td>Chief Investigator</td><td>Affiliation:</td><td></td></tr>
</table>

| | | | | | |
|---|---|---|---|---|---|
| Signature of Chief Investigator: | 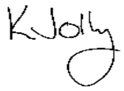 | Date: | 18 Oct 2014 | | Public Health and Primary Care Institute of Applied Health Research |
| **This Statistical Analysis Plan has been approved by:** | | | | | |
| Name of Approver: | Rebecca Woolley | Role: | Senior Statistician | Affiliation: | BCTU |
| Signature of Approver: | Becky Woolley (e-sign) | Date: | 17/10/2024 | | University of Birmingham |

| SAP version number | SAP section number | Description of and reason for change | Timing of change with respect to interim analysis/ final analysis/ database lock | Blind Reviewer | |
|---|---|---|---|---|---|
| 2.0 (changes from v1.0) | 5.4 | After the amendment of the Infant Feeding Helper Contact Log, a note was included in section 5.4 to clarify what the main source of data will be in cases where both summary and individual information have been reported. Furthermore, the text was amended by removing the clarification that the antenatal meeting is not restricted to being antenatal in the case of pre-term births, as all women allocated to the 'planned ABA-feed intervention' who received their initial meeting will be grouped as adherent. | Before the database lock for the final analysis. | Name: | Kelly Handley |
| | | | | Signature: | Kelly Handley (e-signed) |
| | | | | Date: | 21 Oct 2024 |
| | 9.2 | Transformation of responses in the case of skewed continuous data was replaced by reporting unadjusted differences in medians using bootstrapping methods as this is considered a better approach. | | Name: | Kelly Handley |
| | | | | Signature: | Kelly Handley (e-signed) |
| | | | | Date: | 21 Oct 2024 |
| | 9.3 | Amendment of the text to clarify those missing primary outcome data. | | Name: | Kelly Handley |
| | | | | Signature: | Kelly Handley |

| | | | | | |
|---|---|---|---|---|---|
| | | | | | (e-signed) |
| | | | | Date: | 21 Oct 2024 |
| | 9.4 | The manipulations section amended extra details of how to assess the outcomes in case these are not answered.<br><br>Additionally, the outcome ‘Adding formula powder before water’ was amended to ‘Adding formula powder after water’ so that 'YES' responses reflect a positive outcome. | | Name: | Kelly Handley |
| | | | | Signature: | Kelly Handley<br><br>(e-signed) |
| | | | | Date: | 21 Oct 2024 |
| | 9.5 | Addition of the interaction of treatment by IFH to account for the partial cluster design and addition of extra details of how a woman will be clustered in case she had not had her initial meeting with an IFH. | | Name: | Kelly Handley |
| | | | | Signature: | Kelly Handley<br><br>(e-signed) |
| | | | | Date: | 21 Oct 2024 |
| | 9.6 | Addition of the interaction of treatment by IFH to account for the partial cluster design.<br><br>Additionally, the outcome ‘Adding formula powder before water’ was amended to ‘Adding formula powder after water’ so that 'YES' responses reflect a positive outcome. | | Name: | Kelly Handley |
| | | | | Signature: | Kelly Handley<br><br>(e-signed) |
| | | | | Date: | 21 Oct 2024 |
| | 9.7 | Amendment of the Index of Multiple Deprivation subgroup analysis to incorporate up-to-date references and remove details from the quintiles. | | Name: | Kelly Handley |
| | | | | Signature: | Kelly Handley<br><br>(e-signed) |

| | | | | | |
|---|---|---|---|---|---|
| | | | | Date: | 21 Oct 2024 |
| | 9.10 | Addition of the two sensitivity analyses for the skewed continuous outcomes that had been missed. | | Name: | Kelly Handley |
| | | | | Signature: | Kelly Handley<br>(e-signed) |
| | | | | Date: | 21 Oct 2024 |
| | 13 | Amendment of reference 15 to include the most up-to-date IMD tool. | | Name: | Kelly Handley |
| | | | | Signature: | Kelly Handley<br>(e-signed) |
| | | | | Date: | 21 Oct 2024 |

## Statistical Analysis Plan (SAP) Amendments

| Abbreviations & Definitions | |
|---|---|
| **Abbreviation / Acronym** | **Meaning** |
| AE | Adverse Events |
| BCTs | Behaviour Change Techniques |
| BCTU | Birmingham Clinical Trials Unit |
| BFI | Baby Friendly Initiative |
| CI | Confidence Interval |
| CONSORT | Consolidated Standards of Reporting Trials |
| DMC | Data Monitoring Committee |
| EQ-5D-5L | EuroQol Five-Dimensional Five-Level |
| GAD-7 | Generalised Anxiety Disorder Assessment |
| HEAP | Health Economic Analysis Plan |
| IFH | Infant Feeding Helper |
| ISRCTN | International Standard Randomised Controlled Trial Number |
| ITT | Intention to Treat |
| MOS | Medical Outcomes Study |
| PEAP | Process Evaluation Analysis Plan |
| SAE | Serious Adverse Event |
| SAP | Statistical Analysis Plan |
| SUSAR | Suspected Unexpected Serious Adverse Reaction |
| TMG | Trial Management Group |
| TSC | Trial Steering Committee |
| UK | United Kingdom |
| WHO | World Health Organisation |

| Term | Definition |
|---|---|
| International Standard Randomised Controlled Trial Number | A clinical trial registry |
| Protocol | Document that details the rationale, objectives, design, methodology and statistical considerations of the study |
| Randomisation | The process of assigning trial participants to intervention or control groups using an element of chance to determine the assignments in order to reduce bias. |
| Statistical Analysis Plan | Pre-specified statistical methodology documented for the trial, either in the protocol or in a separate document. |

# TABLE OF CONTENTS


1. Introduction ........ 35
2. Background and rationale ........ 35
3. Trial objectives ........ 36
4. Trial methods ........ 37
4.1. Trial design ........ 37
4.2. Trial interventions ........ 37
4.3. Primary outcome measure ........ 37
4.4. Secondary outcome measures ........ 37
4.5. Timing of outcome assessments ........ 39
4.6. Randomisation ........ 39
4.7. Sample size ........ 40
4.8. Framework ........ 40
4.9. Interim analyses and stopping guidance ........ 40
4.10. Internal Pilot Progression Rules ........ 41
4.11. Timing of final analysis ........ 42
4.12. Timing of other analyses ........ 42
4.13. Trial comparisons ........ 42
5. Statistical Principles ........ 42
5.1. Confidence intervals and p-values ........ 42
5.2. Adjustments for multiplicity ........ 42
5.3. Analysis populations ........ 42
5.4. Definition of adherence ........ 43
5.5. Handing protocol deviations ........ 43
5.6. Unblinding ........ 44
6. Trial population ........ 44
6.1. Recruitment ........ 44
6.2. Baseline characteristics ........ 44
7. Intervention(s) ........ 45
7.1. Description of the intervention(s) ........ 45
7.2. Adherence to allocated intervention ........ 45
8. Protocol deviations ........ 45
9. Analysis methods ........ 45
9.1. Covariate adjustment ........ 45
9.2. Distributional assumptions and outlying responses ........ 46
9.3. Handling missing data ........ 46
9.4. Data manipulations ........ 47
9.5. Analysis methods – primary outcome(s) ........ 63
9.6. Analysis methods – secondary outcomes ........ 64
9.7. Analysis methods – exploratory outcomes and analyses ........ 65
9.8. Safety data ........ 66
9.9. Planned subgroup analyses ........ 66
9.10. Sensitivity analyses ........ 67
10. Analysis of sub-randomisations ........ 69
11. Health economic analysis ........ 69
12. Statistical software ........ 69
13. References ........ 69
Appendix A: Deviations from SAP ........ 71

**Appendix B: Trial schema** ........ 72
**Appendix C: Schedule of assessments** ........ 73
**Appendix D: Template report** ........ 74

# 1. Introduction

This document is the Statistical Analysis Plan (SAP) for the ABA-feed trial, and should be read in conjunction with the current trial protocol. This SAP details the proposed analyses and presentation of the data for the main papers reporting the results for the ABA-feed trial.

The results reported in these papers will follow the strategy set out here. Subsequent analyses of a more exploratory nature will not be bound by this strategy, though they are expected to follow the broad principles laid down here. The principles are not intended to curtail exploratory analysis (e.g. to decide cut-points for categorisation of continuous variables), nor to prohibit accepted practices (e.g. transformation of data prior to analysis), but they are intended to establish rules that will be followed, as closely as possible, when analysing and reporting data.

Any deviations from this SAP will be described and justified in the final report or publication of the trial (using a table as shown in Appendix A). The analysis will be carried out by an appropriately qualified statistician, who should ensure integrity of the data during their data cleaning processes.

# 2. Background and rationale

The background and rationale for the trial are outlined in detail in the protocol. In brief, breastfeeding duration in the United Kingdom (UK) is amongst the shortest worldwide; with a rapid drop-off in rates in the first 2-weeks after birth.[1] While the World Health Organisation (WHO) recommend six months exclusive breastfeeding, only 12% of babies in England are exclusively breastfed at 4 months.

A 2017 survey of women's experiences of maternity services identified baby feeding as the greatest area of unmet need for support.[2] Women who report lack of support for breastfeeding difficulties are more likely to discontinue within the first 2 weeks.[1] The current UK policy direction is to increase breastfeeding rates, supported by key policy planks such as the Baby Friendly Initiative (BFI), which recognises that not all mothers will exclusively breastfeed or breastfeed for long durations, and emphasises an approach to support that seeks to 'maximise' the amount of breastmilk infants receive.[3]

The ABA feasibility study was undertaken in two areas with low breastfeeding rates in England. It showed that it was feasible to recruit and train existing paid and volunteer peer supporters to the ABA infant feeding helper (IFH) role; to deliver the intervention with acceptable fidelity; that the ABA intervention was acceptable to women, IFHs and

maternity service staff. The trial processes were feasible with acceptable recruitment and follow-up rates. Intervention contamination in the control group was low and there was no evidence of any intervention related harms. Achievement of timely notification of births was challenging, with a median notification time of 3 days). This resulted in delays in collecting feeding status data at three days and in commencement of postnatal support. Timely birth notification was identified as an aspect that would need to be addressed in the main trial.

Elements of IFH training identified to be in need of improvement in the main trial included using the Friends and Family diagram (genogram) to stimulate conversation, explicit guidance on use of behaviour change techniques and greater focus on active listening skills.

The aim of this study is to assess the clinical and cost-effectiveness of the ABA-feed infant feeding intervention compared to usual care in first-time (nulliparous) mothers.

# 3. Trial objectives

The primary objective is to evaluate if the ABA-feed intervention compared with usual feeding care increases any breastfeeding at 8 weeks post birth, in first-time mothers regardless of their feeding intentions.

Secondary objectives are as follows:

1) To evaluate the effect of the ABA-feed intervention compared to usual feeding care on other feeding outcomes and anxiety.

2) To explore the feasibility of i) modelling longer-term clinical benefits, and ii) costs and outcomes for a lifetime horizon, using a within trial cost-consequence analysis over 16-weeks post birth.

3) To investigate how trial conduct and context varies across sites in order to understand any observed differences in outcomes and inform future implementation.

# 4. Trial methods

## 4.1. Trial design

ABA-feed is a multicentre randomised controlled trial with internal pilot, economic evaluation, and embedded process evaluation (see Appendix B: Trial schema for trial schema).

## 4.2. Trial interventions

The trial interventions are described in detail in the protocol.
Intervention group (planned ABA-feed intervention): the IFH service applying a proactive, assets-based, woman-centred approach, delivered antenatally and postnatally, tailored through texts, video-calls, telephone and face-to-face contacts. A contact at approximately 30 weeks of pregnancy will be followed by texts/brief calls.
Control group (usual feeding care): women will receive the usual care provided for infant feeding within their locality.

## 4.3. Primary outcome measure

The primary outcome is any breastfeeding at 8-weeks post birth, defined in accordance with the UK Infant Feeding Survey as 'infant being breastfed (including being given expressed breastmilk), within the past 24 hours, even if they are also receiving infant formula, solid food or other liquids'.[1]

See section 9.4 on data manipulations for how the primary outcome will be derived.

## 4.4. Secondary outcome measures

The secondary outcomes are clinical and economic. The clinical secondary outcomes are as follows:

- Breastfeeding initiation defined as baby put to the breast, even if this was on one occasion only and includes giving babies expressed breast milk.[1]
- Any breastfeeding at 16-weeks post birth.
- Any breastfeeding at 24-weeks post birth.

- Exclusive breastfeeding at 8-weeks post birth (defined in accordance with the WHO definition of infants who received only breast milk during the previous 24 hours[4]); "Exclusive breastfeeding is defined as the baby receiving no other food or drink, not even water, except breast milk (including milk expressed), but allows the infant to receive oral rehydration solution, drops and syrups (vitamins, minerals and medicines)."
- Exclusive breastfeeding at 16-weeks post birth.
- Exclusive breastfeeding at 24-weeks post birth.
- Time to cease exclusive feeding with breastmilk, up to 16-weeks.
- Time to cease feeding with any breastmilk, up to 16-weeks.
- Maternal anxiety at 8-weeks post birth (measured by the Generalised Anxiety Disorder Assessment (GAD-7)[5]).
- Maternal anxiety at 16-weeks post birth (measured by the GAD-7[5]).
- Maternal health related quality of life at 8-weeks (measured by the EuroQol (EQ-5D-5L)[6]).
- Maternal health related quality of life at 16-weeks (measured by the EuroQol (EQ-5D-5L)[6]).
- Maternal social support at 8-weeks post birth (measured by Medical Outcomes Study (MOS) Emotional / Informational Support domain;[7]).
- Maternal social support at 16-weeks post birth (measured by MOS Emotional / Informational Support domain;[7]).
- The following maternal self-reported formula feeding practices (how formula is prepared) (using questions from the UK Infant Feeding Survey;[1]) at 8-weeks post birth and 16-weeks post birth:
    - Making one feed at a time
    - Correct water temperature
    - Adding formula powder before water
    - Making up formula when needed when out of the home
    - Keeping milk chilled when out of the home
    - Making formula with hot water when out of the home

  - Sterilising bottles using recommended methods

- Maternal use of support for infant feeding (e.g. national breastfeeding helpline; peer support; breastfeeding groups) at 8-weeks post birth and 16-weeks post birth.

- Diagnosis of tongue tie in baby and whether treated, measured at 8-weeks post birth.

- Any infant hospital admission up to 16-weeks post birth associated with feeding mode in the postnatal period, e.g. feeding difficulties, failure to gain weight, jaundice, respiratory or gastrointestinal infection in infants.

See section 9.4 on data manipulations for how the secondary clinical outcomes will be derived. The economic secondary outcomes are listed in the section 8.2.2.2 of the protocol. A Health Economic Analysis Plan (HEAP) will be written separately detailing the economic secondary outcomes.

## 4.5. Timing of outcome assessments

The schedule of trial procedures and outcome assessments are given in Appendix C: **Schedule of assessments**.

## 4.6. Randomisation

The ABA-feed trial is individually randomised. We have inflated the sample size in the intervention group only to account for the effect of potential clustering by feeding helper in the analysis, so randomisation would allocate more women to the intervention arm than control (1606 vs 1124).

Women will be randomised by computer at the level of the individual participant in a 1.43:1 ratio to either ABA-Feed or the control group. A minimisation algorithm will be used within the online randomisation system to ensure balance in the treatment allocation over the following variables:

- site
- woman's age (<25, ≥25)

A 'random element' will be included in the minimisation algorithm, so that each woman has a probability (unspecified here), of being randomised to the opposite treatment that they would have otherwise received. Full details of the randomisation specification will be stored in a confidential document at Birmingham Clinical Trials Unit (BCTU).

Following randomisation, a confirmatory e-mail will be sent to the research team at the relevant Hub and the ABA-feed Trial Office.

## 4.7. Sample size

Assuming 90% power and a 2-sided 5% significance level, with a control group rate of 44% for the primary outcome (95% Confidence Interval (CI) 30.0% to 58.7%; from the ABA feasibility data), a sample size of 2,136 women (1,068 per group) would be required to detect a risk ratio of 1.16 (i.e. an increase of 7% from 44% to 51% in number of breastfeeding at 8-weeks post birth), considered to be a clinically meaningful increase. Since the intervention will be delivered by IFHs, there is a potential for clustering of outcomes by IFH. To allow for this potential clustering effect the sample size for the intervention arm requires inflation, assuming an intra-cluster correlation coefficient of 0.039 taken from ABA feasibility data and given that each IFH will support about 12 women. The sample size required for the intervention arm is thus 1,526, giving a total sample size of 2,594 (1,526 intervention + 1,068 control). Allowing for a 5% loss to follow-up (as in the ABA feasibility study), a total of 2,730 (1,606 intervention and 1,124 control arm) women would be required (2,594/0.95).

Assuming 80% power, the sample size of 2,730 would allow the detection of a risk ratio of 1.14 equivalent to a 6% absolute increase.

With an average 12 women/IFH we need to train 134 peer supporters (1,606/12).

## 4.8. Framework

The objective of the trial is to test the superiority of one intervention to another.

The null hypothesis is that there is no difference in any breastfeeding at 8 weeks between the intervention groups. The alternative hypothesis is that there is a difference between the groups.

## 4.9. Interim analyses and stopping guidance

Interim analyses of safety and efficacy for presentation to the independent Data Monitoring Committee (DMC) will take place during the study. There is a separate DMC reporting template that has been agreed by the DMC, and this includes agreement on which outcomes will be reported at interim analyses. The statistical methods stated in this SAP will be followed for the outcomes included in the DMC report, where possible. Criteria for stopping or modifying the study based on this

information will be ratified by the DMC. Details of the agreed plan will be written into a DMC Charter.

The DMC will be scheduled to meet annually. An emergency meeting may also be convened if a safety issue is identified. The DMC will report directly to the Trial Steering Committee who will convey the findings of the DMC to the Trial Management Group (TMG), Sponsors and funders.

The DMC may consider recommending the discontinuation of the trial if the recruitment rate or data quality are unacceptable or if any issues are identified which may compromise participant safety.

## 4.10. Internal Pilot Progression Rules

An embedded internal pilot will run in all units over a period of six months to assess site recruitment and rates of recruitment of women into the trial. Pre-specified progression criteria have been agreed as follows:

| | **Red** | **Amber** | **Green** |
|---|---|---|---|
| Number of sites open | ≤10 | 11-14 | 15 |
| Number of IFHs trained | <107<br>(<80% planned) | 107-133<br>(80-99% planned) | 134<br>(100% planned) |
| Cumulative recruitment target | <546<br>(<20% sample size) | 546-818<br>(20-29% sample size) | 819<br>(≥30% sample size) |
| Actions | Discuss with TSC and consider recommending stopping the trial | Discuss with TSC strategies for improvement and consider changes to processes including opening further recruiting centres/sites | Continue |

In the light of the ongoing uncertainties during the COVID-19 pandemic and ongoing disruption to maternity care and Research and Development Services, additional actions (e.g. increasing number of recruiting centres opened and/or recruiting additional peer supporters at each site) to support recruitment may be necessary to achieve the pilot targets in an appropriate timeframe.

## 4.11. Timing of final analysis

The final analysis of the ABA-feed trial will occur once all participants have completed the 24 week assessment and the corresponding outcome data has been entered onto the trial database and validated as being ready for analysis. This is provided that the trial has not been stopped early for any reason (e.g. Trial Steering Committee (TSC)/DMC advice or funding body request).

## 4.12. Timing of other analyses

Not applicable.

## 4.13. Trial comparisons

All references in this document to 'group' refer to 'planned ABA-feed intervention' or 'usual feeding care'.

# 5. Statistical Principles

## 5.1. Confidence intervals and p-values

All estimates of differences between groups will be presented with two-sided 95% confidence intervals, unless otherwise stated. P-values will be reported from two-sided tests at the 5% significance level.

## 5.2. Adjustments for multiplicity

No correction for multiple testing will be made.

## 5.3. Analysis populations

All primary analyses (primary and secondary outcomes including safety outcomes) will be by intention-to-treat (ITT). All trial participants will be analysed in the intervention group to which they were randomised irrespective of adherence or other protocol deviation apart from pregnancy loss, stillbirth, infant death or maternal death prior to an outcome assessment, where participants will be excluded from the analysis of outcomes after the date of loss/death. In such instances, the primary analysis population will be based on the modified ITT population. Additionally, every attempt

will be made to collect full follow-up data on all trial participants; it is thus anticipated that missing data will be minimal. In the ABA-Feed feasibility study, most women who did not respond to the follow-up questionnaire were formula feeding and so trial participants who missing primary outcome data will be deemed to not be breast feeding. This presents a risk of bias, and sensitivity analyses will be undertaken to assess the possible impact of the risk. In brief, this will include analyses of only those participants with primary outcome data available, and also worst-case/best case assumptions for those with missing data (i.e. worst-case assumptions: those are randomised in the intervention arm will be deemed to not be breastfeeding, and those who are randomised in the usual care arm will be deemed to be breastfeeding; best-case assumptions: those are randomised in the intervention arm will be deemed to be breastfeeding, and those who are randomised in the usual care arm will be deemed to not be breastfeeding). See section 9.10 for further details on the sensitivity analyses.

## 5.4. Definition of adherence

Adherence to allocated intervention delivery by IFHs will be monitored by the IFH Intervention log and will be evaluated by the process evaluation analysis. Details of this analysis will be provided in a separate Process Evaluation Analysis Plan (PEAP).

For the per-protocol population of women, adherence will be defined as follows:

- For women allocated to the 'planned ABA-feed intervention' – if they received their initial meeting and at least one other contact made postnatally with an IFH, they are considered adherent;
- For women allocated to the 'usual feeding care' – if they have not received any contact with an IFH, then they are considered adherent.

**Note:** In cases where both summary and individual information have been reported in Infant Feeding Helper Contact Log Form, the summary information will be used as the primary data.

## 5.5. Handing protocol deviations

A protocol deviation is defined as a failure to adhere to the protocol such as errors in applying the inclusion/exclusion criteria, the incorrect intervention being given, incorrect data being collected or measured, follow-up visits outside the visit window or missed follow-up visits. We will apply a strict definition of the ITT principle and will include all participants as per the ITT population described in section 5.3 in the analysis, in some form, regardless of deviation from the protocol.[8] This does not

include those participants who have specifically withdrawn consent for the use of their data in the first instance; however, these outcomes will be explored as per other missing responses.

Women randomised to the planned ABA-Feed intervention where there was no attempt to contact them by an ABA-Feed IFH, or women randomised to receive the usual feeding care and received an antenatal meeting with an ABA-Feed IFH will be treated as protocol deviations.

Women who do not adhere to the definition of adherence in section 5.4 will not be treated as a protocol deviation.

All data collected will be valid even if they are collected outside of schedule assessment windows as given in the table of schedule of assessments in Appendix C: **Schedule of assessments**.

## 5.6. Unblinding

Not applicable as ABA-FEED is an open-label trial. The DMC will review interim data without being masked to treatment allocation. Unblinding of the Trial Statistician to the allocated intervention code will take place when monitoring and cleaning the data, when producing each interim DMC report, and after the database is locked for final analysis.

# 6. Trial population

## 6.1. Recruitment

A flow diagram (as recommended by CONSORT[9]) will be produced to describe the participant flow through each stage of the trial. This will include information on the number (with reasons) of losses to follow-up (drop-outs and withdrawals) over the course of the trial. A template for reporting this is given in ABA-Feed Final Report template.

## 6.2. Baseline characteristics

The trial population will be tabulated as per ABA-Feed Final Report template. Categorical data will be summarised by number of participants, counts and percentages. Continuous data will be summarised by the number of participants, mean and standard deviation if deemed to be normally distributed or number of

participants, median and interquartile range if data are skewed, and ranges if appropriate. Tests of statistical significance will not be undertaken, nor confidence intervals presented.[10]

# 7. Intervention

## 7.1. Description of the intervention

A template for reporting information on the intervention is given in ABA-Feed Final Report template.

## 7.2. Adherence to allocated intervention

A cross-tabulation of allocated intervention by the adherence categories stated in section 5.4 will be produced (proportions and percentages). A template for reporting adherence is given in ABA-Feed Final Report template.

# 8. Protocol deviations

Frequencies and percentages by group will be tabulated for the protocol deviations as per ABA-Feed Final Report template.

# 9. Analysis methods

Intervention groups will be compared using regression modelling approaches (e.g. linear mixed effects models, log-binomial regression, Poisson regression) to adjust for all covariates as specified in section 9.1, where possible.

## 9.1. Covariate adjustment

In the first instance, intervention effects between groups for all outcomes will be adjusted for the minimisation parameters listed in section 4.6 and IFH in order to account for the partial cluster design. Age will be treated as a continuous variable in this adjustment.

If covariates adjustment is not possible (e.g. the model does not converge), alternative models will be considered. For example, in binary outcomes, if a log-binomial model fails to converge, a Poisson regression model with robust standard errors will be used to estimate the same parameters.[11] If this also fails to converge, in the initial log-

binomial model the site covariate will be removed in the first instance and then if there are still converging issues the site covariate will be replaced and the IFH covariate will be removed instead. Should the model still fail to converge unadjusted estimates will be produced from the log-binomial model. It will be made clear in the final report why this occurred (e.g. not possible due to low event rate/lack of model convergence).

Other covariate adjustment will use baseline values for parameters where available (e.g. an analysis of Maternal anxiety scores at 8 & 16 weeks will also include the baseline score as a covariate in the model and the same will be for the analysis of the Maternal health related quality of life scores at 8 & 16 weeks and the analysis of the Maternal social support scores at 8 & 16 weeks).

Site and IFH will be treated as a random effect in the models, and all other factors as fixed effects.

## 9.2. Distributional assumptions and outlying responses

Distributional assumptions (e.g. normality of regression residuals for continuous outcomes) will be assessed visually prior to reporting the results of the analysis; although in the first instance the proposed primary method of estimation in this analysis plan will be followed. If distributional assumptions are considered to be particularly skewed and/or distributional assumptions violated, the impact of this will be examined through sensitivity analysis; this may consist of the use of medians and Inter Quartile Ranges (IQRs) alongside unadjusted differences in medians using bootstrapping methods (repetition=1000, seed=150824). If extreme values are apparent and considered to be affecting the integrity of the analysis, a sensitivity analysis consisting of removing the outlying response(s) and repeating the analysis will be performed. Output from these analyses, if performed, will be described and presented alongside the original analysis (or included, e.g. in appendices) with the excluded values clearly labelled. See section 9.10 for further details regarding sensitivity analyses.

## 9.3. Handling missing data

Every effort will be made to follow-up participants to minimise any potential bias due to missing data. However, the primary analysis of the primary outcome will be conducted on data from all trial participants, including those with missing primary outcome data as formula feeding. This is excluding those with pregnancy loss, stillbirth, infant death or maternal death prior to the 8 week assessment. See section

5.3 for further details. To examine the possible impact of imputing missing data on the results, and to make sure we are complying with the intention-to-treat principle, sensitivity analyses will be performed on the primary outcome measure.[12] See section 9.10 for further details regarding sensitivity analyses.

Those missing the following secondary outcome data: any breastfeeding at 16-weeks post birth, and any breastfeeding at 24-weeks post birth will be analysed as per the primary analysis of the primary outcome. All the other secondary analyses will be conducted on received data only.

## 9.4. Data manipulations

The Trial Statistician will derive all responses from the raw data recorded in the database.

**Primary outcome:**

**Any breastfeeding at 8-weeks post birth, defined in accordance with the UK Infant Feeding Survey as infant being breastfed (including being given expressed breastmilk), within the past 24 hours, even if they are also receiving infant formula, solid food or other liquids.**

Reported on the Questionnaire Follow Up 1 by answering the question:

Q6. Thinking about the milk that your baby has received over the last 24 hours, has he/she had…

A binary outcome will be defined as:

- YES if the answer to Q6 is "*Only breast milk*" or "*Breast milk and infant formula*" and;
- NO if the answer to Q6 is "*Only infant formula*".

In the case that the Follow Up Form 1 is missing, or response to Q6 is missing then assess the primary outcome using the 8 week text message response to the question:

*We would like to know how your baby has been fed IN THE LAST 24 HOURS.*

If this question is still missing then assess the outcome from the Health Visitor Form and the responses on the question:

*Q2.3 - Please specify feeding status at time of assessment*

Furthermore, if the responses to the above are all missing and the Questionnaire Follow Up 2 has been completed then the binary primary outcome will be changed from missing to YES if the answer to question(s):

Q2. Thinking about the milk that your baby has received over the last 24 hours, has he/she had is**:** "*Only breast milk*" or "*Breast milk and infant formula*",

OR

Q2. Thinking about the milk that your baby has received over the last 24 hours, has he/she had is**:** "*Only infant formula" AND*

Q4 Has you baby EVER been given breast milk (via syringe, bottle or cup etc) or have you put your baby to the breast, even if this was only once? Is: "*Yes (even if only once)"* AND

Q5. How old was your baby when he/she was LAST given breast milk or you put them to your breast? Is greater or equal to 42 days.

Else, the primary outcome will remain as missing.

**Secondary outcomes:**

**Breastfeeding initiation defined as baby put to the breast, even if this was on one occasion only and includes giving babies expressed breast milk**

Reported on the Questionnaire Follow Up 1 by answering the question(s):

Q6. Thinking about the milk that your baby has received over the last 24 hours, has he/she had…

Q8. Has your baby EVER been given breast milk (via syringe, bottle or cup etc.) or have you put your baby to the breast, even if this was only once?

And on the Questionnaire Follow Up 2 by answering the question(s):

Q2. Thinking about the milk that your baby has received over the last 24 hours, has he/she had…

Q4. Has your baby EVER been given breast milk (via syringe, bottle or cup etc) or have you put your baby to the breast, even if this was only once?

A binary outcome will be defined as:

- YES if the answer to Q6 is "*Only breast milk*" or "*Breast milk and infant formula*" **OR** if the answer to Q6 is "*Only infant feed formula*" and Q8 is "*Yes (even if only once)*" **OR** if the answer to Q2 is "*Only breast milk*" or "*Breast milk and infant formula*" **OR** if the answer to Q2 is "*Only infant feed formula*" and Q4 is "*Yes (even if only once)*") and;

- NO if the answer to Q6 is "*Only infant feed formula*" and for Q8 is "*No*" **OR** if the answer to Q2 is "*Only infant feed formula*" and Q4 is "*No*".

**Note:** In the case that the secondary outcome 'Breastfeeding initiation' is missing then the outcome will be assessed by the responses on the question:

*We would like to know how your baby has been fed since birth.*

Reported on the 3-day questionnaire and will be defined as:

- YES if the answer to this question is "*Your baby has had only breast milk*" or "*Your baby has had both breast and formula milk*" and;
- NO if the answer to this question is "*Your baby has had only formula milk*".

**Any breastfeeding at 16-weeks post birth**

Reported on the Questionnaire Follow Up 2 by answering the question:

Q2. Thinking about the milk that your baby has received over the last 24 hours, has he/she had…

A binary outcome will be defined as:

- YES if the answer to Q2 is "*Only breast milk*" or "*Breast milk and infant formula*" and;
- NO if the answer to Q2 is "*Only infant formula*".

In the case that the Follow Up 2 Form is missing, or response to Q2 is missing then the outcome any breastfeeding at 16 weeks will be changed from missing to YES if the answer to question on the Follow Up 3 Form:

Q2. Thinking about the milk that your baby has received over the last 24 hours, has he/she had is**:** "*Only breast milk*" or "*Breast milk and infant formula*",

**Any breastfeeding at 24-weeks post birth**

Reported on the Questionnaire Follow Up 3 by answering the question:

Q2. Thinking about the milk that your baby has received over the last 24 hours, has he/she had…

A binary outcome will be defined as:

- YES if the answer to Q2 is "*Only breast milk*" or "*Breast milk and infant formula*" and;
- NO if the answer to Q2 is "*Only infant formula*".

**Exclusive breastfeeding at 8-weeks post birth (defined in accordance with the WHO definition of infants who received only breast milk during the previous 24 hours); "Exclusive breastfeeding is defined as the baby receiving no other food or drink, not even water, except breast milk (including milk expressed), but allows the infant to receive oral rehydration solution, drops and syrups (vitamins, minerals and medicines)"**

Reported on the Questionnaire Follow Up 1 by answering the questions:

Q6. Thinking about the milk that your baby has received over the last 24 hours, has he/she had…

Q13. Apart from milk, has your baby received any other drinks or solid food over the last 24 hours? (Do not include any medicines or vitamin or mineral supplements).

A binary outcome will be defined as:

- YES if the answer to Q6 is "*Only breast milk*" and the answer to Q13 is "*No*" and;
- NO if the answer to Q13 is "*Yes*" **OR** the answer to Q6 is "*Only infant formula*" or "*Breast milk and infant formula*".

**Exclusive breastfeeding at 16-weeks post birth**

Reported on the Questionnaire Follow Up 2 by answering the questions:

Q2. Thinking about the milk that your baby has received over the last 24 hours, has he/she had…

Q12. Apart from milk, has your baby received any other drinks or solid food over the last 24 hours? (Do not include any medicines or vitamin or mineral supplements)

A binary outcome will be defined as:

- YES if the answer to Q2 is "*Only breast milk*" and the answer to Q12 is "*No*" and;
- NO if the answer to Q12 is "*Yes*" **OR** the answer to Q2 is "*Only infant formula*" or "*Breast milk and infant formula*".

**Exclusive breastfeeding at 24-weeks post birth**

Reported on the Questionnaire Follow Up 3 by answering the questions:

Q2. Thinking about the milk that your baby has received over the last 24 hours, has he/she had…

Q3. Apart from milk, has your baby received any other drinks over the last 24 hours? (Do not include any medicines or vitamin or mineral supplements)

Q4. In the past 24 hours has your baby received any cereal, rusks, baby rice or any other solid food?

A binary outcome will be defined as:

- YES if the answer to Q2 is "*Only breast milk*", the answer to Q3 is "*No*" and the answer to Q4 is "*No*" and;
- NO if the answer to Q2 is "*Only infant formula*" or "*Breast milk and infant formula*" **OR** the answer to Q3 is "*Yes*" OR the answer to Q4 is "*Yes*".

**Time to cease exclusive feeding with breastmilk, up to 16-weeks**

If a women meets the exclusive breast feeding at 16 weeks post birth (see above definition) then they will be considered to have exclusively breastfed up to 16 weeks and they will be censored at the point of form completion. Their time to cease variable will be calculated from Follow Up 2 Questionnaire as 'today's date' – 'date of baby's birth'.

If a women does not meet the exclusive breast feeding at 16 weeks post birth (see above definition) then the time to cease variable will be calculated by looking in the first instance at the Follow Up 2 Questionnaire and the answers to the following questions:

Q2. Thinking about the milk that your baby has received over the last 24 hours, has he/she had…

Q6. How old was your baby when he/she FIRST received infant formula?

Q7. Has your baby EVER had anything else to drink apart from milk, such as water, fruit juice, squash or herbal drink?

Q8. How old was your baby when he or she was FIRST given something apart from milk to drink, such as water, fruit juice or herbal drink?

Q9. Has your baby ever had any foods such as cereal, rusks, baby rice, fruit, vegetables or any other kind of solid food?

Q10. How old was your baby when he/she first had any food apart from milk?

Q12. Apart from milk, has your baby received any other drinks or solid food over the last 24 hours? (Do not include any medicines or vitamin or mineral supplements)

If the answer to Q2 is:

- "*Only breast milk*"

  then time to cease exclusive breastfeeding will be calculated from Q8 or Q10 (since Q12 must have been answered "*Yes*" for them not to be exclusively breastfeeding at 16 weeks). If the answer to Q8 is provided and as weeks, then this will be converted to days by multiplying by 7 then adding any additional days. If the answer to Q10 is provided then this answer will be converted to days by multiplying by 7. The time to cease exclusive breastfeeding will be as follows:

  - Time in days for Q8 if only answer to Q8 is provided
  - Time in days for Q10 if only answer to Q10 is provided
  - If answers to both Q8 and Q10 are provided then the smallest time in days for Q8 and Q10 will be used

If the answer to Q2 is:

- "*Breast milk and infant formula*" OR "*Only infant formula*"

  the time to cease exclusive breastfeeding will be calculated from Q6, Q7, Q8, Q9 and Q10. If the answer to Q6 is provided as weeks, then this will be converted to days by multiplying by 7 then adding any additional days. If Q7 is answered as "*Yes*" and Q8 is provided in weeks then this will be converted to days in the same way as Q6. If Q9 is answered as "*Yes*" then Q10 will be converted to days by multiplying by 7. The time to cease exclusive breastfeeding will be:

  - Time in days for Q6 if Q7 is answered as "*No*" and Q9 is answered as "*No*"
  - If Q7 is answered as "*Yes*" then the smallest time in days for Q6 and Q8 will be used

- If Q9 is answered as "*Yes*" then the smallest time in days for Q6 and Q10 will be used
- If both Q7 and Q9 are answered as "*Yes*" then the smallest time in days for Q6,Q8 and Q10 will be used

In the event that we do not receive the Follow Up 2 Questionnaire (due to loss to follow up/withdrawal etc.), but have received the Follow Up 1 Questionnaire, a participant can still be included in the analysis up until 8 weeks using the following questions:

Q2. What date was your baby born?

Q6. Thinking about the milk that your baby has received over the last 24 hours, has he/she had…

Q 10. How old was your baby when he/she FIRST received infant formula?

Q11. Has your baby EVER had anything else to drink apart from milk, such as water, fruit juice, squash or herbal drink? (Do not include any medicines or vitamin or mineral supplements)

Q12. How old was your baby when he or she was FIRST given something apart from milk to drink, such as water, fruit juice or herbal drink?

Q13. Apart from milk, has your baby received any other drinks or solid food over the last 24 hours?

A woman will be considered to be exclusively breast feeding at 8 weeks if they answer "*Only breast milk*" for Q6 and "*No*" to Q13. Then time to cease exclusive breastfeeding will then be calculated as 'today's date' – 'date of baby's birth'. They will be censored at this time point.

Else if:

- If the answer to Q6 is "*Only breast milk*" and "*Yes*" to Q13:

  Time to cease exclusive breastfeeding will be calculated from Q12. If the answer to Q12 is provided and as weeks, then this will be converted to days by multiplying by 7 then adding any additional days. The time to cease exclusive breastfeeding will be as follows:

  - Time in days for Q12

- If the answer to Q6 is "*Breast milk and infant formula*" OR "*Only infant formula*":

the time to cease exclusive breastfeeding will be calculated from Q10, Q11 and Q12. If the answer to Q10 is provided as weeks, then this will be converted to days by multiplying by 7 then adding any additional days. If Q11 is answered as "*Yes*" and Q12 is provided in weeks then this will be converted to days in the same way as Q6. The time to cease exclusive breastfeeding will be:

- Time in days for Q10 if Q11 is answered as "*No*"
- If Q11 is answered as "*Yes*" then the smallest time in days for Q10 and Q12 will be used

**Time to cease feeding with any breastmilk, up to 16-weeks**

Reported on the Questionnaire Follow Up 2 by answering the questions:

Q1. What is today's date?

Q2. Thinking about the milk that your baby has received over the last 24 hours, has he/she had

Q4. Has your baby EVER been given breast milk (via syringe, bottle or cup etc) or have you put your baby to the breast, even if this was only once?

Q5. How old was your baby when he/she was LAST given breast milk or you put them to your breast?

A woman will be considered to be feeding with any breast milk at 16 weeks if they answer Q2 as "*Only breast milk*" or '' *Breast milk and infant formula*". Then time to cease breastfeeding will then be calculated as 'today's date' – 'date of baby's birth'. They will be censored at this time point.

Else if:

- Q2 is answered as "*Only infant formula*" and Q4 is answered as "*No*":

  time to cease feeding with any breastmilk will be set as 0.1 days

- Q2 is answered as "*Only infant formula*" and Q4 is answered as *"Yes":*

  time to cease feeding with any breast milk will be calculated using the answer to Q5. If the answer to Q5 is provided as weeks, then this will be converted to days by multiplying by 7 then adding any additional days. This will be the time to cease feeding with any breast milk.

In the event that we do not receive the Follow Up 2 Questionnaire (due to loss to follow up/withdrawal etc.), but have received the Follow Up 1 Questionnaire, a participant can still be included in the analysis up until 8 weeks using the following questions:

Q2. What date was your baby born?

Q6. Thinking about the milk that your baby has received over the last 24 hours, has he/she had…

Q8. Has your baby EVER been given breast milk (via syringe, bottle or cup etc.) or have you put your baby to the breast, even if this was only once?

Q9. How old was your baby when he/she was LAST given breast milk or you put them to your breast?

A woman will be considered to be feeding with any breast milk at 8 weeks if they answer Q6 as "*Only breast milk*" or " *Breast milk and infant formula*". Then time to cease feeding with any breast milk will then be calculated as 'today's date' – 'date of baby's birth'. They will be censored at this time point.

Else if:

- Q6 is answered as "*Only infant formula*" and Q8 is answered as "*No*":

  time to cease feeding with any breastmilk will be set as 0.1 days

- Q6 is answered as "*Only infant formula"* and Q8 is answered as *"Yes":*

  time to cease feeding with any breast milk will be calculated using the answer to Q9. If the answer to Q9 is provided as weeks, then this will be converted to days by multiplying by 7 then adding any additional days. This will be the time to cease feeding with any breast milk.

**Maternal anxiety at 8-weeks post birth (measured by the Generalised Anxiety Disorder Assessment (GAD-7)**

Reported on the Questionnaire Follow Up 1 by answering the questions:

Q26.1 - Feeling nervous, anxious or on edge

Q26.2 - Not being able to stop or control worrying

Q26.3 - Worrying too much about different things

Q26.4 - Trouble relaxing

Q26.5 - Being so restless that it is hard to sit still

Q26.6 - Becoming easily annoyed or irritable

Q26.7 - Feeling afraid as if something awful might happen

Each of the 7 is scored from 0 - Not at all to 3 - Nearly every day. The GAD-7 total score will be calculated by adding together the scores for the seven questions and ranges from 0 to 21 with 0 indicates lack of anxiety and 21 indicates the highest level of anxiety. No missing data items are permitted in order to compute a score.

**Maternal anxiety at 16-weeks post birth (measured by the GAD-7)**

Reported on the Questionnaire Follow Up 2 by answering the questions:

Q22.1 - Feeling nervous, anxious or on edge

Q22.2 - Not being able to stop or control worrying

Q22.3 - Worrying too much about different things

Q22.4 - Trouble relaxing

Q22.5 - Being so restless that it is hard to sit still

Q22.6 - Becoming easily annoyed or irritable

Q22.7 - Feeling afraid as if something awful might happen

The GAD-7 total score at 16 weeks will be calculated the same as the GAD-7 total score at 8 weeks.

**Maternal health related quality of life at 8-weeks (measured by the EuroQol (EQ-5D-5L)**

Reported on the Questionnaire Follow Up 1 by answering the questions:

Q27a – MOBILITY

Q27b - SELF-CARE

Q27c - USUAL ACTIVITIES (e.g. work, study, housework, family or leisure activities)

Q27d - PAIN / DISCOMFORT

Q27e - ANXIETY / DEPRESSION

The EQ-5D-5L questionnaire essentially consists of the EQ-5D descriptive system and the EQ visual analogue scale (EQ VAS). For the purpose of this trial, only the EQ-5D descriptive system will be collected. The descriptive system comprises five dimensions: mobility, self-care, usual activities, pain/discomfort and anxiety/depression. Each dimension has 5 levels: 1 = no problems, 2 = slight problems, 3 = moderate problems, 4 = severe problems and 5 = extreme problems.

A unique health state is defined by combining one level from each of the five dimensions. A total of 3125 possible health states is defined in this way. Each state is referred to by a 5-digit code. For example, following the order: Mobility, Self-care, Usual activities, Pain/Discomfort, and Anxiety/Depression, state 12345 indicates no problems with mobility, slight problems with washing or dressing, moderate problems with doing usual activities, severe pain or discomfort and extreme anxiety or depression, while state 11111 indicates no problems on any of the five dimensions. For deriving the EQ-5D-5L score, the position statement of the National Institute for Health and Care Excellence (NICE)[13] will be followed, which indicates to derive the 5L score by mapping the 5L descriptive system data onto the 3L value set. No missing data items are permitted in order to compute a score.

**Maternal health related quality of life at 16-weeks (measured by the EQ-5D-5L)**

Reported on the Questionnaire Follow Up 2 by answering the questions:

Q23a – MOBILITY

Q23b - SELF-CARE

Q23c - USUAL ACTIVITIES (e.g. work, study, housework, family or leisure activities)

Q23d - PAIN / DISCOMFORT

Q23e - ANXIETY / DEPRESSION

The EQ-5D-5L total score at 16 weeks will be calculated the same as the EQ-5D-5L total score at 8 weeks.

**Maternal social support at 8-weeks post birth (measured by Medical Outcomes Study (MOS) Emotional / Informational Support domain)**

Reported on the Questionnaire Follow Up 1 by answering the questions:

Q43a - Someone you can count on to listen to you when you need to talk

Q43b - Someone to give you information to help you understand a situation

Q43c - Someone to give you good advice about a crisis

Q43d - Someone to confide in or talk to about yourself or your problems

Q43e - Someone whose advice you really want

Q43f - Someone to share your most private worries and fears with

Q43g - Someone to turn to for suggestions about how to deal with a personal problem

Q43h - Someone who understands your problems

The MOS questionnaire represents four functional support scales: Emotional/Informational, Tangible, Affectionate, and Positive Social Interaction. For the purpose of this trial, only the Emotional/Informational dimension will be collected, which consists of 8 items. Each item has five possible answers: 1 = None of the time, 2 = A little of the time, 3 = Some of the time, 4 = Most of the time, 5 = All of the time. No items need to be reversed (all items are phrased so that a higher item score reflects a higher level of social support). Maternal social support at 16-weeks post birth (measured by MOS Emotional / Informational Support domain;[7])

The respondent-specific mean scores of the 8 items will be calculated ignoring items with missing values. This allows respondents with at least one valid response to receive a score. Next the mean subscale score will be transformed to have a possible range 0-100, with higher scores indicating more support. The scale score can be transformed to a 0-100 scale using the following formula:

$$100 \times \frac{(\text{mean subscale score} - 1)}{(5 - 1 = 4)}$$

So if for example, 5 out of the 8 items were answered in a combination of 1 2 3 4 5, the mean subscale score would be (1 + 2 + 3 + 4 + 5)/5=15/5=3. The mean subscale score would then be:

$$100 \times (3-1)/(5-1) = 50$$

A scale score of 0 indicates lower level of support and 100 indicates a higher level of support.

**Maternal social support at 16-weeks post birth (measured by MOS Emotional / Informational Support domain)**

Reported on the Questionnaire Follow Up 2 by answering the questions:

Q38a - Someone you can count on to listen to you when you need to talk

Q38b - Someone to give you information to help you understand a situation

Q38c - Someone to give you good advice about a crisis

Q38d - Someone to confide in or talk to about yourself or your problems

Q38e - Someone whose advice you really want

Q38f - Someone to share your most private worries and fears with

Q38g - Someone to turn to for suggestions about how to deal with a personal problem

Q38h - Someone who understands your problems

The MOS total score at 16 weeks will be calculated the same as the MOS total score at 8 weeks.

**Maternal use of support for infant feeding (e.g. national breastfeeding helpline; peer support; breastfeeding groups) at 8-weeks post birth and 16-weeks post birth**

Reported on the Questionnaire Follow Up 1 by answering the question:

Q22. How many times have you had contact with the following people to discuss feeding your baby since your baby was born?

Reported on the Questionnaire Follow Up 2 by answering the question:

Q21. How many times have you had contact with the following people to discuss feeding your baby IN THE LAST TWO MONTHS?

The numbers and percentages reported in each source of support in the above two questions will be presented separately at 8 weeks and after 8 weeks up to 16 weeks.

**The following maternal self-reported formula feeding practices (how formula is prepared) (using questions from the UK Infant Feeding Survey;) at 8-weeks and 16 weeks post birth:**

The following outcomes will be only be reported for those women who use infant formula at all at 8 weeks or 16 weeks.

- **Making one feed at a time**

  Reported on the Questionnaire Follow Up 1 & 2 by answering the question:

  Q14/Q13. When making infant formula feeds do you USUALLY…

It is defined as:

- YES if Q14/Q13 is answered as "*Only make one feed at a time as you need it*" or *"Only ever use ready to feed formula"* and;
- NO if Q14/Q13 is answered as "*Make several feeds at a time and store them"*.

- **Correct water temperature**

Reported on the Questionnaire Follow Up 1 & 2 by answering the question:

Q15/Q14. When making infant formula feeds for your baby do you USUALLY…

It is defined as:

- YES if Q15/Q14 is answered as "*Use water that has just boiled*" or "*Use water that has boiled and been left to cool for 30 minutes"* and;
- NO if Q15/Q14 is answered as "*Use water that has boiled and been left to cool between 30 and 45 minutes*" or "*Use water that has boiled and been left to cool for more than 45 minutes*" or "*Use an infant feeding machine"*

- **Adding formula powder after water**

Reported on the Questionnaire Follow Up 1 & 2 by answering the question:

Q16/Q15. When making infant formula feeds do you USUALLY…

It is defined as:

- YES if Q16/Q15 is answered as "*Put the water in the bottle first and then add the powder*"
- NO if Q16/Q15 is answered as "*Put the powder in the bottle first and then add the water*" and;

- **Making up formula when needed when out of the home**

Reported on the Questionnaire Follow Up 1 & 2 by answering the question:

Q17/Q16. If you need to feed your baby when you are out do you USUALLY…

It is defined as:

- YES if Q17/Q16 is answered as "*Make up an infant formula feed whilst you are out*" and;
- NO if Q17/Q16 is answered as "*Make up infant formula feed before leaving home*" and;

- Not Applicable if Q17/Q16 is answered as "*Take a ready to feed formula with you*" or "*Take expressed breast milk with you*" or "*Breastfeed*" or "*Never feed your baby away from home*"

- **Keeping milk chilled when out of the home**

The following is only applicable for those who are defined as NO for the above i.e. they make up formula before leaving home. Reported on the Questionnaire Follow Up 1 & 2 these participants will answer the question:

Q18/Q17. When you are out, do you USUALLY keep feeds you have made chilled?

It is defined as:

- YES if Q18/Q17 is answered as "*Yes*" and;
- NO if Q18/Q17 is answered as "*No*"

- **Making formula with hot water when out of the home**

The following is only applicable for those who are defined as YES for making up formula when out of the home. Reported on the Questionnaire Follow Up 1 & 2 these participants will answer question:

Q19/Q18. When you are out do you USUALLY…

It is defined as:

- YES if Q19/Q18 is answered as "*Make feeds with hot water (e.g. ask for hot water or use hot water from a flask)*" and;
- NO if Q19/Q18 is answered as "*Make feeds with cold or cooled water"*

- **Sterilising bottles using recommended methods**

The following is only applicable for those who answered "*Yes*" to the question:

Q20/Q19. Have you ever used a bottle to feed your baby?

Reported on the Questionnaire Follow Up 1 & 2 and by answering the question:

Q21/Q20. What methods do/did you USUALLY use to sterilise the bottle?

It is defined as:

- YES if Q21/Q20 is answered as "*Boiling water*" or "*Soaking in sterilising solution e.g. Milton*" or "*Steam steriliser*" or "*Microwave*";
- NO if Q21/Q20 is answered as "*Hot soapy water*" or "*Dishwasher*" and;

- if Q21/Q20 is answered as "*Other*" then this will be classed as YES or NO where possible after review of other method.

**Diagnosis of tongue tie in baby and whether treated, measured at 8-weeks post birth**

Reported on the Questionnaire Follow Up 1 by answering the questions:

Q23. Has your baby ever been diagnosed with a tongue-tie?

Q24. Has your baby had a frenotomy (tongue tie procedure)?

The numbers and percentages reported in these two questions will be presented descriptively.

**Any infant hospital admission up to 16-weeks post birth associated with feeding mode in the postnatal period, e.g. feeding difficulties, failure to gain weight, jaundice, respiratory or gastrointestinal infection in infants**

Reported on the Questionnaire Follow Up 1 by answering the questions:

Q30. Has your baby been admitted to hospital for any reason since birth?

Q31. How many times has your baby been admitted to hospital (for any duration) since birth?

Q32. Please give the reason for the admission/s

Then admission associated with feeding mode at 8 weeks is defined as:

- YES if Q30 is answered as "*Yes*" and at least one of the following items: Q32a(Not gaining weight or weight loss) or Q32b (Feeding difficulties) or Q32c (Jaundice) or Q32d (Breathing difficulties) or Q32e (Diarrhoea and/or vomiting) are answered as "*First hospital admission*", "*Second hospital admission*" or "*Third hospital admission*" and;

- NO if Q30 is answered as "*No*" **OR** if Q30 is answered as 'Yes' and neither Q32a or Q32b or Q32c or Q32d or Q32e are answered.
- If Q30 is missing then admission associated with feeding mode at 16 weeks will be missing.

Reported on the Questionnaire Follow Up 2 by answering the questions:

Q26. Has your baby been admitted to hospital for any reason in the LAST TWO MONTHS?

Q27. How many times has your baby been admitted to hospital (for any duration) in the LAST TWO MONTHS?

Q28. Please give the reason for the admission/s

Then admission associated with feeding mode at 16 weeks in the last two month is defined as:

- YES if Q26 is answered as "*Yes*" and at least one of the following items:
  Q28a(Not gaining weight or weight loss) or Q28b (Feeding difficulties) or Q28c (Jaundice) or Q28d (Breathing difficulties) or Q28e (Diarrhoea and/or vomiting) are answered as "*First hospital admission*", "*Second hospital admission*" or "*Third hospital admission*" and;

- NO if Q26 is answered as "*No*" **OR** if Q26 is answered as 'Yes' and neither Q28a or Q28b or Q28c or Q28d or Q28e are answered.
- If Q26 is missing then admission associated with feeding mode at 16 weeks will be missing.

Then the outcome any infant hospital admission up to 16-weeks post birth associated with feeding mode in the postnatal period, e.g. feeding difficulties, failure to gain weight, jaundice, respiratory or gastrointestinal infection in infants is defined as:

- YES if admission associated with feeding mode at 8 weeks is "*Yes*" **OR** admission associated with feeding mode at 16 weeks is "*Yes*" and;
- NO if both admissions associated with feeding mode at 8 and 16 weeks are "*No*".

**Note:** If the question: 'What date was your baby born?' is missing from the Follow-up 1 Questionnaire or the answer is different from the Infant Date of Birth reported in the Infantbirth dataset in REDCap then the date reported in the Infantbirth dataset will be assumed to be correct.

## 9.5. Analysis methods – primary outcome

A template for reporting the primary outcome is given in ABA-Feed Final Report template.

The primary outcome (i.e. baby receiving any breast milk' at 8-weeks post birth) is a binary outcome (i.e. yes/no) based on a partially nested design, will be summarised presenting number of responses with percentages and analysed using a mixed effects log binomial regression model, adjusting for the intervention group, the minimisation variables listed in section 4.6 (age and site) and the interaction of treatment by IFH.

Age (as a continuous variable) will be treated as a fixed effect and site and IFH will be treated as random effects. The treatment effect will be expressed as an adjusted risk ratio and a risk difference with associated 95% CIs. If the model does not converge, alternative models will be considered, e.g. log Poisson regression models with robust variance estimation.[11] The p-value from the associated model will be produced and used to determine statistical significance of the estimated treatment group parameter.

In the event that a woman has more than one IFH, she will be clustered to the IFH with whom she had her initial meeting. If a woman has not had her initial meeting then she will be assigned to the IFH with whom she has had the most contacts, however in the instance there are IFHs she has had an equal number of visits with the choice of IFH will be discussed with the CI. We will report the percentage of women who had multiple IFHs and how many different IFHs they had. However, if a woman has more than one IFH, we will conduct a sensitivity analysis removing her from the analysis. See section 9.1 for covariate adjustment and model convergence and section 9.10 for sensitivity analyses.

## 9.6. Analysis methods – secondary outcomes

A template for reporting the secondary outcomes is given in ABA-Feed Final Report template.

The binary secondary outcomes (i.e. breastfeeding initiation; any breastfeeding at 16-weeks and 24-weeks post birth; exclusive breastfeeding at 8-, 16- and 24 weeks; any infant hospital admission up to 16-weeks post birth associated with feeding mode in the postnatal period) will be summarised presenting number of responses with percentages and analysed using the same methods described for the primary outcome (section 9.5), and results presented as adjusted risk ratios, risk differences with corresponding 95% confidence intervals and p-values.

For time to cease exclusive feeding with breastmilk (days) and time to cease feeding with any breastmilk (days) intervention groups will be compared using survival analysis methods. Kaplan-Meier survival curves will be constructed for visual presentation of time-to-event comparisons. A Cox proportional hazards model with mixed effects will be fitted to obtain an adjusted hazard ratios and 95% confidence intervals and associated 2-sided p-values. The model will be adjusted for the continuous covariate age, which will be treated as fixed effect and site and IFH which will be treated as random effects. The interaction of treatment by IFH will be also included in the model.

For those secondary outcomes that are continuous (i.e. anxiety measured by the GAD-7 at 8-weeks and 16-weeks; health related quality of life measured by the EQ-5D-5L

at 8 and 16-weeks; social support measured at 8- and 16-weeks), means of responses with standard deviation will be presented and mixed effects linear regression methods will be used if the outcome is sufficiently normally distributed (or where data can be suitably transformed), adjusting for the minimisation variables listed in section 6.2 and baseline measures where relevant, including IFH and site as random effects, to calculate an adjusted mean difference with 95% confidence intervals and associated 2-sided p-values. The interaction of treatment by IFH will be also included in the model.

The following maternal self-reported formula feeding practices (how formula is prepared) (using questions from the UK Infant Feeding Survey;[1]) at 8-weeks post birth and 16-weeks post birth:

- Making one feed at a time,
- Correct water temperature,
- Adding formula powder after water,
- Making up formula when needed when out of the home,
- Keeping milk chilled when out of the home,
- Making formula with hot water when out of the home,
- Sterilising bottles using recommended methods;

The maternal use of support for infant feeding at 8- and 16-weeks post birth; the diagnosis of tongue tie in baby and whether treated, measured at 8-weeks post birth will be presented descriptively presenting number of responses with percentages.

## 9.7. Analysis methods – exploratory outcomes and analyses

Any data that does not form a pre-specified outcome will be presented using simple summary statistics by intervention group (i.e. numbers and percentages for binary data and means (or medians) and standard deviations (or inter-quartile ranges) for continuous normal (or non-normal) data.

## 9.8. Safety data

Given the low risk nature of the intervention, no formal evaluation of Serious Adverse Events (SAEs) will be required. However, during follow-up we will systematically collect self-reported data from participants regarding admissions to hospital by infant and mother requiring an overnight admission and the reasons for this. We will also capture whether there have been any infant deaths and cause of death. These will be reviewed by the DMC at regular intervals. These data will be presented with numbers and percentages or means, standard deviations and range accordingly.

A template for reporting this safety data is given in ABA-Feed Final Report template.

## 9.9. Planned subgroup analyses

Interpretation of subgroup analysis will be treated with caution (output will be treated as exploratory rather than definitive[14]). Analysis will be limited to the primary outcome only, and the following subgroups:

- Woman's age
    - <25 years; and
    - ≥25 years.
- Pre-specified as feeding intentions
    - Breast milk only;
    - Mainly breast milk;
    - Half and half breast and formula milk;
    - Mainly formula; and
    - Formula milk only.
- Mother's education
    - No formal qualification;
    - GCSE, Standard Grade, National 5 or equivalent;
    - A-level/AS level or equivalent; and
    - Degree level or above.

If 'other' has been recorded, it will be grouped into one of the four categories based on the provided specification. If 'other' has not been specified then it will be excluded from the analysis.

- Index of Multiple Deprivation (IMD), using the UK Index of Multiple Deprivation data file, which was created by the Bristol University[15] however, if we find a more complete up-to-date IMD tool[16] we will consider using it. The code_area (LSAO) will be linked to the postcodes using the file: PCD_OA21_LSOA21_MSOA21_LAD_NOV23_UK_LU form the Office for national statistics[17]
    - 1st quintile group (least deprived);
    - 2nd quintile group;
    - 3rd quintile group;
    - 4th quintile group; and
    - 5th quintile group (most deprived).
- Relationship status
    - Married or in a registered civil partnership, or Living together; and
    - Single, or widowed, divorced or separated.

If 'Prefer not say' has been selected then it will be excluded from the analysis.

The effects of these subgroups will be examined by log-binomial regression model analysis, using independent variables as the interaction between intervention arm and each of the sub-group covariates in turn. P-values from tests for statistical heterogeneity (e.g. by including the intervention group by subgroup interaction parameter in the final regression model) will be presented alongside the adjusted relative risk and 95% confidence intervals within subgroups. The results of subgroup analyses will be treated with caution and will be used for the purposes of hypothesis generation only.

A template for reporting the subgroup analyses for the primary outcome is given in ABA-Feed Final Report template.

## 9.10. Sensitivity analyses

The following sensitivity analyses will be limited to the primary outcome and will consist of:

- Per-protocol analysis (population described in sections 5.3 and 5.4);
- Two analyses to assess the effect of missing responses:
  - a complete case analysis by excluding from the primary analysis participants with missing the primary outcome data, and
  - analyses implementing worst-case/best-case assumptions: in the first instance those with missing data in the ABA-FEED group will be assumed to be formula feeding, and those with missing data in the usual care group will be assumed to be breastfeeding; and in the second instance those with missing data in the ABA-FEED group will be assumed to be breastfeeding, and those in the usual care group will be assumed to be formula feeding;
- An analysis to assess the effect of imputing the missing responses using a tipping point analysis approach. Unadjusted models will be used. Firstly, in the ABA-FEED group only, a participant who initially missed the primary outcome data and had been included in the primary analysis assuming to be formula feeding will be removed from the total number of participants in the ABA-FEED group and an unadjusted model will be fitted. Breastfeeding rates will be compared between the ABA-FEED and usual care groups. The CI from the treatment estimate (from the risk ratio of the log-binomial model) will be examined. If the upper/lower limit of this CI is different to that of the primary analysis finding in regards to whether the CI crosses the null value of one then proceed no further and conclude one less non-event as the tipping point for the ABA-FEED group. If the CI from the model is the same as the primary finding (i.e. the CI still does/does not cross the null value of one) then proceed to remove another participant from the ABA-FEED group, who initially missed the primary outcome data and had been included in the primary analysis assuming to be formula feeding and repeat this process. The tipping point analysis for the ABA-FEED group will finish when either enough participants have been removed such that the CI differs from that of the primary analysis finding or the number of participants added is equal to the number of participants with missing outcome data in the ABA-FEED group and there is no difference in the CI. Results will be produced visually. The event rate in the ABA-FEED group will be on the x-axis, and the RR (ABA-FEED vs usual care) from the model will be on the y-axis. A CI will be included around each RR. If the CI in the primary analysis contains one, then the point where the upper CI falls below one will be highlighted (tipping point). If the CI in the primary analysis does not contain one (i.e. one intervention is superior to the other), then the point where the CI crosses one will be highlighted (tipping point). This process will be then be repeated for the usual care group.
- In the case of women having more than one IFH, a sensitivity analysis removing women with more than one IFH and repeating the analysis will be performed.

The following sensitivity analyses, which are limited to the continuous secondary outcomes and will be conducted only if distributional assumptions are deemed particularly skewed, will consist of:

- An analysis to assess the effect of any distributional assumptions (as described in section 9.2);
- An analysis to assess the effect of any outliers' assumptions (as described in section 9.2).

# 10. Analysis of sub-randomisations

Not applicable.

# 11. Health economic analysis

As indicated in the protocol there will also be an economic analysis. The details of this analysis are documented separately.

# 12. Statistical software

Statistical analysis will be undertaken in the following statistical software packages:

- SAS software, version 9.4 (or higher)
- Stata version 16 (or higher)

# 13. References


1. McAndrew F, Thompson J, Fellows L, Large A, Speed M, Renfrew MJ. Infant feeding survey 2010. Leeds: Health and Social Care Information Centre 2012.
2. The National Federation of Women's Institutes and NCT. Support Overdue: Women's experiences of maternity services. NCT; 2017.
3. Unicef. Maximising Breastmilk. Unicef; 2019. URL: https://www.unicef.org.uk/babyfriendly/baby-friendly-resources/implementing-standards-resources/maximising-breastmilk/ (Accessed 11/02/2021).
4. World Health Organization. Indicators for assessing infant and young child feeding practices, Part 3: country profiles. 2010.

5. Spitzer RL, Kroenke K, Williams JB, Lowe B. A brief measure for assessing generalized anxiety disorder: the GAD-7. Arch Intern Med 2006;166:1092-7.

6. Herdman M, Gudex C, Lloyd A, Janssen M, Kind P, Parkin D, et al. Development and preliminary testing of the new five-level version of EQ-5D (EQ-5D-5L). Quality of Life Research 2011;20:1727-36.

7. Sherbourne CD, Stewart AL. The MOS social support survey. Social Science and Medicine 1991;32:705-14.

8. Gupta SK. Intention-to-treat concept: A review. Perspect Clin Res. 2011;2(3):109-112.

9. Schulz KF, Altman DG, Moher D, for the CONSORT Group. CONSORT 2010 Statement: updated guidelines for reporting parallel group randomised trials. BMJ. 2010;340:c332.

10. Altman DG, Dore CJ. Randomisation and baseline comparisons in clinical trials. Lancet. 1990;335:149–53.

11. Zou G. A modified Poisson regression approach to prospective studies with binary data. Am J Epidemiol. 2004;159(7):702-6.

12. White IR, Horton NJ, Carpenter J, Pocock SJ. Strategy for intention to treat analysis in randomised trials with missing outcome data. BMJ. 2011;342:d40.

13. NICE URL: https://www.nice.org.uk/about/what-we-do/our-programmes/nice-guidance/technology-appraisal-guidance/eq-5d-5l. This link was accessed in June 2021.

14. Wand R, Lagakos SW, Ware JH, Hunter DJ, Drazen JM. Reporting of subgroups analyses in clinical trials. NEJM. 2007;357:2189-94.

15. IMD tool: https://data.bris.ac.uk/data/dataset/1ef3q32gybk001v77c1ifmty7x. This link was accessed in Oct 2024.

16. IMD tool: https://data.cdrc.ac.uk/dataset/index-multiple-deprivation-imd#data-and-resources. This link was access in Oct 2024.

17. https://geoportal.statistics.gov.uk/datasets/18b6cc7d58bb413f814237b6dfa1f5c9/about. This link was accessed in Oct 2024.

# Appendix A: Deviations from SAP

This report below follows the statistical analysis plan dated <*insert effective date of latest SAP*> apart from following:

**Section of report not following SAP**

**Reason**

<insert section >

<insert, e.g. exploratory analyses request by TMG>

# Appendix B: Trial schema

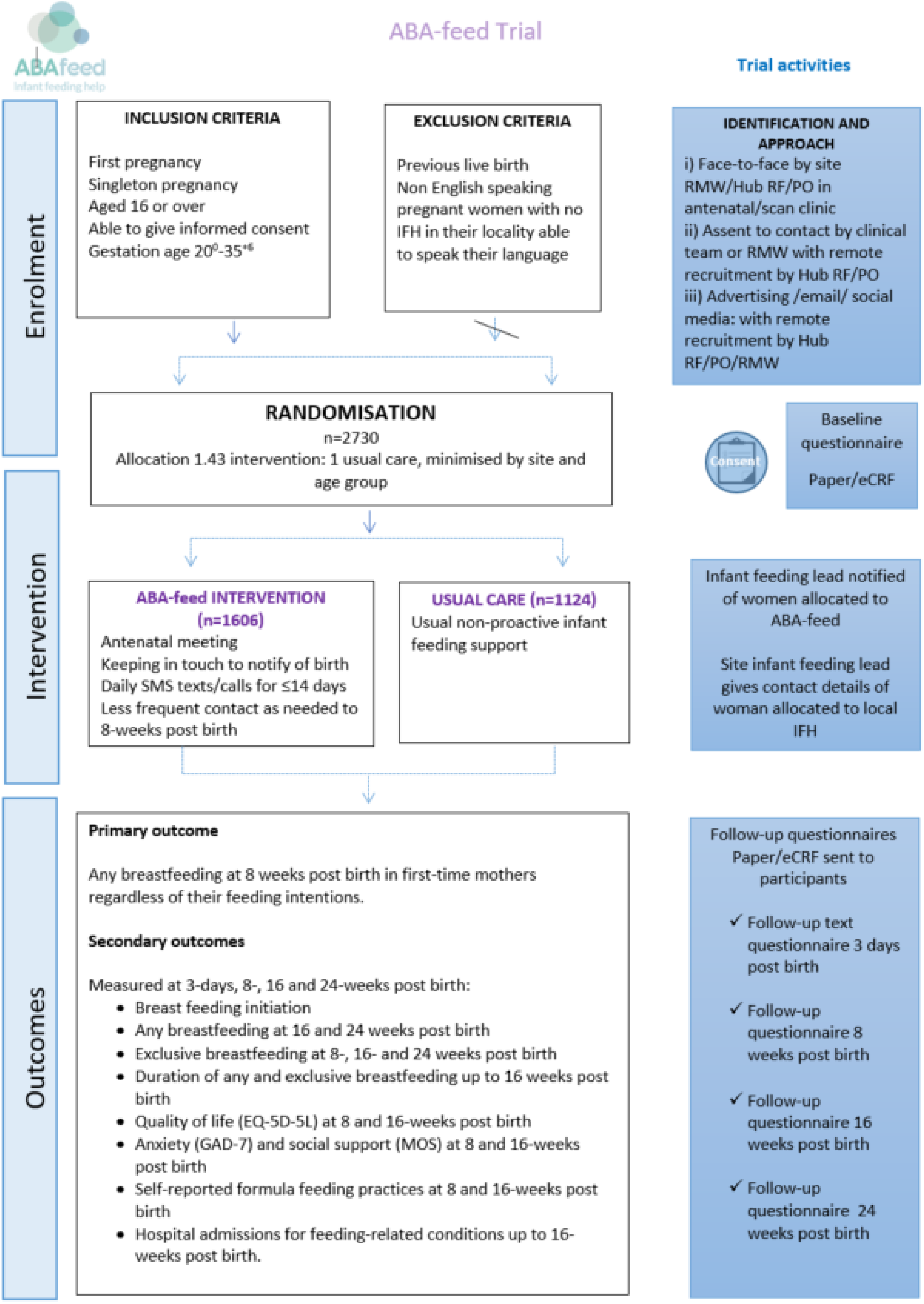

# Appendix C: Schedule of assessments

Trial participant schedule of events and summary of assessments:

| *Visit* | *Screening (before 36/40) weeks gestation* | *Baseline (before 36/40) gestation weeks* | *Text day 3 post birth (+ 10 days)* | *Week 8 post birth (+ 30 or – 14 days)* | *Week 16 post birth (+ 30 or – 14 days)* | *Week 24 post birth (-14 to +10 days)* |
|---|---|---|---|---|---|---|
| *Eligibility check* | *x* | | | | | |
| *Valid informed consent* | *x* | | | | | |
| *Relevant obstetric history taken* | *x* | | | | | |
| *Demographic data* | *x* | | | | | |
| *Infant feeding plans* | *x* | | | | | |
| *Randomisation* | | *x* | | | | |
| *Infant feeding status* | | | *x* | *x* | *x* | *x* |
| *Details of mode of birth* | | | | *x* | | |
| *Health & social resource use* | | | | *x* | *x* | |
| *Infant feeding difficulties* | | | | *x* | | |
| *Self-reported formula feeding practices* | | | | *x* | *x* | |
| *EQ-5D-5L* | | *x* | | *x* | *x* | |
| *GAD-7* | | *x* | | *x* | *x* | |
| *MOS social support* | | *x* | | *x* | *x* | |
| *Infant deaths* | | *x* | *x* | *x* | *x* | |

# Appendix D: Template report

A template report for the final analyses will be provided in a separate document.

**Supplementary file: Statistical analysis code**

**SAS code:**

```
/*Primary outcome ITT analysis-Any breastfeeding at 8 weeks post birth*/
/*Summary of the primary outcome before the imputation of the missing values as NO for non-pregnancy
losses*/
proc sort data=Prout_data;
by descending pr_outcome;
run;
proc freq data=Prout_data order=data;
table pr_outcome*treatment/nopercent norow;
run;

proc sort data=Prout_data;
by descending pr_outcome;
run;
proc freq data=Prout_data order=data;
table pr_outcome*treatment/missing nopercent norow;
run;

/*Bring the pregnancy loss, stillbirth, infant death data*/
data Preglossinfdeath (keep=record_id plid_stllbrth plid_infntdeathdte index_loss);
set clean.Preglossinfdeath;
index_loss=1;
run;

proc sort data=Preglossinfdeath out=Preglossinfdeath;
by record_id ;
run;
proc sort data=Prout_data out=Prout_data;
by record_id ;
run;

data Prout_data;
merge Prout_data (in=in1 )
        Preglossinfdeath (in=in2);
by record_id;
if in1 then output;
run;

/*Recode the missing data of the primary outcome to imput them as 'NO'*/
data Prout_data;
set Prout_data;
if pr_outcome=. and index_loss = . then pr_outcome=0;
else if pr_outcome=. and index_loss = 1 then pr_outcome=.;
run;

proc sort data=Prout_data;
by descending pr_outcome;
run;
proc freq data=Prout_data order=data;
table pr_outcome*treatment/nopercent norow;
run;

/*Summary of primary outcome data*/
proc sort data=Prout_data;
```

```
by descending pr_outcome;
run;
proc freq data=Prout_data order=data;
table pr_outcome*treatment/missing nopercent norow;
run;
/*Risk Ratio*/
proc glimmix data=Prout_data;
class treatment(ref='Usual Care') age_cat(ref='1 - 16 yrs<=AGE<25 yrs') site;
model pr_outcome = treatment age_cat / dist=binomial link=log solution cl;
random Intercept / subject=site;
lsmeans treatment / cl diff=all;
estimate "Risk Ratio" treatment 1 -1 /exp cl;
nloptions tech= nrridg;
run;

/*Risk Difference*/
proc glimmix data=Prout_data;
class treatment(ref='Usual Care') age_cat(ref='1 - 16 yrs<=AGE<25 yrs') site;
model pr_outcome = treatment age_cat/ dist=binomial link=identity solution cl;
random Intercept / subject=site;
lsmeans treatment / cl diff=all;
estimate "Risk difference" treatment 1 -1 /cl;
nloptions tech= none;
run;

/*Primary outcome Subgroup analysis*/
/*------------------------------------------------------------------------------------------------------------------------
            Subgroup 1 - Woman's age

                Proc GLIMMIX age: categorical & site: random effect
------------------------------------------------------------------------------------------------------------------------*/
/*Summary*/
proc sort data=subgroup_data;
by  age_cat;
run;
proc freq data=subgroup_data order=data;
run;

/*Risk Ratio*/
proc glimmix data=subgroup_data ;
class treatment (ref='Usual Care') age_cat (ref='2 - AGE>=25 yrs') site;
model pr_outcome = treatment age_cat treatment*age_cat / dist=binomial link=log solution cl;
random Intercept / subject=site;
slice Treatment*age_cat /sliceby=age_cat diff exp cl plots=none;/*Risk Ratio per subgroup */
lsmestimate  Treatment*age_cat 'Risk Ratio in the subgroup age<25' 1 0 -1 0 /exp CL; /* same as the above
line risk ratio per subgroup*/
lsmestimate  Treatment*age_cat 'Risk Ratio in the subgroup age>=25' 0 1 0 -1 /exp CL;
lsmestimate  Treatment*age_cat 'Ratio: age<25/age>=25' 1 -1 -1 1/exp CL; /* same as the above line risk ratio
per subgroup*/
nloptions tech= nrridg;
run;
/*------------------------------------------------------------------------------------------------------------------------
            Subgroup 2 - Pre-specified as feeding intentions

                Proc GLIMMIX age: categorical & site: random effect
------------------------------------------------------------------------------------------------------------------------*/
```

```
/*Summary*/
proc sort data=subgroup_data;
by  base_milk6mnths;
run;
proc freq data=subgroup_data order=data;
table base_milk6mnths*pr_outcome*treatment/ nopercent norow;
run;

/*Risk Ratio*/
/*Model was not converging thus site was removed*/
proc glimmix data=subgroup_data ;
class treatment (ref='Usual Care') age_cat (ref='2 - AGE>=25 yrs')  base_milk6mnths (ref='Breast milk only');
model pr_outcome = treatment age_cat base_milk6mnths treatment*base_milk6mnths / dist=binomial
link=log solution cl;
slice Treatment*base_milk6mnths /sliceby=base_milk6mnths diff exp cl plots=none;/*Risk Ratio per subgroup
*/
lsmestimate  Treatment*base_milk6mnths 'Risk Ratio in the subgroup Formula milk only' 1 0 0 0 0 -1 0 0 0
0/exp CL; /* same as the above line risk ratio per subgroup*/
lsmestimate  Treatment*base_milk6mnths 'Risk Ratio in the subgroup Half and half breast and formula milk'
0 1 0 0 0 0 -1 0 0 0 /exp CL;
lsmestimate  Treatment*base_milk6mnths 'Risk Ratio in the subgroup  Mainly breast milk' 0 0 1 0 0 0 0 -1 0 0
/exp CL;
lsmestimate  Treatment*base_milk6mnths 'Risk Ratio in the subgroup Mainly formula ' 0 0 0 1 0 0 0 0 -1 0 /exp
CL;
lsmestimate  Treatment*base_milk6mnths 'Risk Ratio in the subgroup Breast milk only' 0 0 0 0 1 0 0 0 0 -1 /exp
CL;
lsmestimate  Treatment*base_milk6mnths 'Ratio: Mainly breast milk/Breast milk only' 0 0 1 0 -1 0 0 -1 0 1/exp
CL;
  lsmestimate  Treatment*base_milk6mnths 'Ratio: Half and half breast and formula milk/Breast milk only' 0 1
0 0 -1 0 -1 0 0 1/exp CL;
  lsmestimate  Treatment*base_milk6mnths 'Ratio: Mainly formula/Breast milk only' 0 0 0 1 -1 0 0 0 -1 1/exp
CL;
lsmestimate  Treatment*base_milk6mnths 'Ratio: Formula milk only/Breast milk only' 1 0 0 0 -1 -1 0 0 0 1/exp
cl;
nloptions tech= nrridg;
run;
/*-----------------------------------------------------------------------------------------------------------------------------------
              Subgroup 3 - Mother's education

                   Proc GLIMMIX age: categorical & site: random effect
-----------------------------------------------------------------------------------------------------------------------------------*/
/*Summary*/
proc sort data=subgroup_data;
by  base_hghstlqlfctn;
run;
proc freq data=subgroup_data order=data;
table base_hghstlqlfctn*pr_outcome*treatment/ nopercent norow;
run;

/*Risk Ratio*/
/*Model was not converging thus site was removed*/
proc glimmix data=subgroup_data ;
class treatment (ref='Usual Care') age_cat (ref='2 - AGE>=25 yrs')  base_hghstlqlfctn (ref='Degree level or
above');
model pr_outcome = treatment age_cat base_hghstlqlfctn treatment*base_hghstlqlfctn / dist=binomial
link=log solution cl;
```

```
slice Treatment*base_hghstlqlfctn /sliceby=base_hghstlqlfctn diff exp cl plots=none;/*Risk Ratio per subgroup
*/
lsmestimate  Treatment*base_hghstlqlfctn 'Risk Ratio in the subgroup A-level/AS level or equivalent' 1 0 0 0   -
1 0 0 0 /exp CL; /* same as the above line risk ratio per subgroup*/
lsmestimate  Treatment*base_hghstlqlfctn 'Risk Ratio in the subgroup GCSE, Standard Grade, National 5 or
equivalent'   0 1 0 0   0 -1 0 0  /exp CL;
lsmestimate  Treatment*base_hghstlqlfctn 'Risk Ratio in the subgroup  No formal qualification' 0 0 1 0   0 0 -1
0  /exp CL;
lsmestimate  Treatment*base_hghstlqlfctn 'Risk Ratio in the subgroup Degree level or above' 0 0 0 1   0 0 0 -1
/exp CL;
lsmestimate  Treatment*base_hghstlqlfctn 'Ratio: No formal qualification/Degree level or above' 0 0 1 -1   0 0
-1 1 /exp CL;
lsmestimate  Treatment*base_hghstlqlfctn 'Ratio: GCSE, Standard Grade, National 5 or equivalent/Degree
level or above'  0 1 0 -1   0 -1 0 1 /exp CL;
lsmestimate  Treatment*base_hghstlqlfctn 'Ratio: A-level/AS level or equivalent/Degree level or above'  1 0 0 -
1   -1 0 0 1 /exp CL;
nloptions tech= nrridg;
run;
/*-------------------------------------------------------------------------------------------------------------------------------------
                Subgroup 4 - Index of Multiple Deprivation (IMD)

                     Proc GLIMMIX age: categorical & site: random effect
-------------------------------------------------------------------------------------------------------------------------------------*/
/*Summary*/
proc sort data=subgroup_data;
by  IMD_quintile descending pr_outcome treatment;
run;
proc freq data=subgroup_data order=data;
table IMD_quintile*pr_outcome*treatment/ nopercent norow;

run;

/*Risk Ratio*/
proc glimmix data=subgroup_data ;
class treatment (ref='Usual Care') age_cat (ref='2 - AGE>=25 yrs') site IMD_quintile (ref='1st quintile group
(most deprived)');
model pr_outcome = treatment age_cat IMD_quintile treatment*IMD_quintile / dist=binomial link=log
solution cl;
random Intercept / subject=site;
slice Treatment*IMD_quintile /sliceby=IMD_quintile diff exp cl plots=none;/*Risk Ratio per subgroup */
lsmestimate  Treatment*IMD_quintile 'Risk Ratio in the subgroup 2nd quintile group' 1 0 0 0 0   -1 0 0 0 0 /exp
CL; /* same as the above line risk ratio per subgroup*/
lsmestimate  Treatment*IMD_quintile 'Risk Ratio in the subgroup 3rd quintile group'   0 1 0 0 0   0 -1 0 0 0
/exp CL;
lsmestimate  Treatment*IMD_quintile 'Risk Ratio in the subgroup  4th quintile group' 0 0 1 0 0   0 0 -1 0 0
/exp CL;
lsmestimate  Treatment*IMD_quintile 'Risk Ratio in the subgroup 5th quintile group(least deprived))' 0 0 0 1 0
0 0 0 -1 0 /exp CL;
lsmestimate  Treatment*IMD_quintile 'Risk Ratio in the subgroup 1st quintile group(most deprived))' 0 0 0 0 1
0 0 0 0 -1  /exp CL;

lsmestimate  Treatment*IMD_quintile 'Ratio: 2nd quintile group/5th quintile group(least deprived)'  1 0 0 -1 0
-1 0 0 1 0 /exp CL;
lsmestimate  Treatment*IMD_quintile 'Ratio: 3rd quintile group/5th quintile group(least deprived)'  0 1 0 -1 0
0 -1 0 1 0 /exp CL;
lsmestimate  Treatment*IMD_quintile 'Ratio: 4th quintile group/5th quintile group(least deprived)' 0 0 1 -1 0
0 0 -1 1 0 /exp CL;
```

```
lsmestimate  Treatment*IMD_quintile 'Ratio: 1st quintile group(most deprived)/5th quintile group(least
deprived)'  0 0 0 -1 1    0 0 0 1 -1 /exp CL;
nloptions tech= nrridg;
run;
/*-----------------------------------------------------------------------------------------------------------------------------------
               Subgroup 5 - Relationship status

                    Proc GLIMMIX age: categorical & site: random effect
-----------------------------------------------------------------------------------------------------------------------------------*/
/*Summary*/
proc sort data=subgroup_data;
by  rel_status;
run;
proc freq data=subgroup_data order=data;
table rel_status*pr_outcome*treatment/ nopercent norow;
run;

/*Risk Ratio*/
proc glimmix data=subgroup_data ;
class treatment (ref='Usual Care') age_cat (ref='2 - AGE>=25 yrs') site rel_status (ref='Single, or widowed,
divorced or separated');
model pr_outcome = treatment age_cat rel_status treatment*rel_status / dist=binomial link=log solution cl;
random Intercept / subject=site;
slice Treatment*rel_status /sliceby=rel_status diff exp cl plots=none;/*Risk Ratio per subgroup */
lsmestimate  Treatment*rel_status 'Risk Ratio in the subgroup Married or in a registered civil partnership, or
Living together' 1 0 -1 0 /exp CL; /* same as the above line risk ratio per subgroup*/
lsmestimate  Treatment*rel_status 'Risk Ratio in the subgroup Single, or widowed, divorced or separated' 0 1 0
-1 /exp CL;
lsmestimate  Treatment*rel_status 'Ratio: Married or in a registered civil partnership, or Living
together/Single, or widowed, divorced or separated' 1 -1 -1 1/exp CL; /*same as the above line risk ratio per
subgroup*/
nloptions tech= nrridg;
run;

/*Secondary outcome ITT analysis-Breastfeeding initiation*/
/*Summary*/
proc sort data=BF_initiation;
by descending BF_initiation treatment;
run;
proc freq data=BF_initiation order=data;
table BF_initiation*treatment /  missing nopercent norow;
run;

proc sort data=BF_initiation;
by descending BF_initiation treatment;
run;
proc freq data=BF_initiation order=data;
table BF_initiation*treatment /   nopercent norow;
run;

/*Risk Ratio*/
proc glimmix data=BF_initiation;
class treatment (ref='Usual Care') age_cat (ref='1 - 16 yrs<=AGE<25 yrs');
model BF_initiation = treatment age_cat/ dist=binomial link=log solution cl;
lsmeans treatment / cl diff=all;
estimate "Risk Ratio" treatment 1 -1 / exp cl;
```

```
nloptions tech= nrridg;
run;

/*Risk Difference */
proc glimmix data=BF_initiation;
class treatment (ref='Usual Care') age_cat (ref='1 - 16 yrs<=AGE<25 yrs');
model BF_initiation = treatment age_cat/ dist=binomial link=identity solution cl;
lsmeans treatment / cl diff=all ;
estimate "Risk Difference" treatment 1 -1 / cl;
nloptions tech= none;
run;

/*Secondary outcome ITT analysis-Any breastfeeding 16wks*/
/*Recode the missing data of the primary outcome to impute them as 'NO'*/
data BF_16wks;
set BF_16wks;
if BF_16wks=. and index_loss = . then BF_16wks=0;
else if BF_16wks=. and index_loss = 1 then BF_16wks=.;
run;

/*Summary*/
proc sort data=BF_16wks;
by descending BF_16wks treatment;
run;
proc freq data=BF_16wks order=data;
table BF_16wks*treatment /  missing nopercent norow;
run;

proc sort data=BF_16wks;
by descending BF_16wks treatment;
run;
proc freq data=BF_16wks order=data;
table BF_16wks*treatment /   nopercent norow;
run;

/*Risk Ratio*/
proc glimmix data=BF_16wks ;
class treatment (ref='Usual Care') age_cat (ref='1 - 16 yrs<=AGE<25 yrs') site;
model BF_16wks = treatment age_cat / dist=binomial link=log solution cl;
random Intercept / subject=site;
lsmeans treatment / cl diff=all;
estimate "Risk Ratio" treatment 1 -1 / exp cl;
nloptions tech= nrridg;
run;
/*Risk Difference*/
proc glimmix data=BF_16wks ;
class treatment  (ref='Usual Care') age_cat (ref='1 - 16 yrs<=AGE<25 yrs') site;
model BF_16wks = treatment age_cat/ dist=binomial link=identity solution cl;
random Intercept / subject=site ;
lsmeans treatment / cl diff=all;
estimate "Risk difference" treatment 1 -1 / cl;
nloptions tech= none;
run;

/*Secondary outcome ITT analysis-Any breastfeeding 24wks*/
/*Recode the missing data of the primary outcome to impute them as 'NO'*/
```

```
data BF_24wks;
set BF_24wks;
if BF_24wks=. and index_loss = . then BF_24wks=0;
else if BF_24wks=. and index_loss = 1 then BF_24wks=.;
run;

/*Summary*/
proc sort data=BF_24wks;
by descending BF_24wks treatment;
run;
proc freq data=BF_24wks order=data;
table BF_24wks*treatment /  missing nopercent norow;
run;

proc sort data=BF_24wks;
by descending BF_24wks treatment;
run;
proc freq data=BF_24wks order=data;
table BF_24wks*treatment /   nopercent norow;
run;
title4; run;

/*Risk Ratio*/
proc glimmix data=BF_24wks ;
class treatment (ref='Usual Care') age_cat (ref='1 - 16 yrs<=AGE<25 yrs') site;
model BF_24wks = treatment age_cat / dist=binomial link=log solution cl;
random Intercept / subject=site;
lsmeans treatment / cl diff=all;
estimate "Risk Ratio" treatment 1 -1 / exp cl;
nloptions tech= nrridg;
run;

/*Risk Difference*/
proc glimmix data=BF_24wks ;
class treatment  (ref='Usual Care') age_cat (ref='1 - 16 yrs<=AGE<25 yrs') site;
model BF_24wks = treatment age_cat/ dist=binomial link=identity solution cl;
random Intercept / subject=site ;
lsmeans treatment / cl diff=all;
estimate "Risk difference" treatment 1 -1 / cl;
nloptions tech= none;
run;

/*Secondary outcome ITT analysis-Exclusive breastfeeding 8wks*/
/*Summary*/
proc sort data=EBF_8wks;
by descending EBF_8wks treatment;
run;
proc freq data=EBF_8wks order=data;
table EBF_8wks*treatment /  missing nopercent norow;
run;

proc sort data=EBF_8wks;
by descending EBF_8wks treatment;
run;
proc freq data=EBF_8wks order=data;
table EBF_8wks*treatment /   nopercent norow;
```

```
run;

/*Risk Ratio*/
proc glimmix data=EBF_8wks ;
class treatment (ref='Usual Care') age_cat(ref='1 - 16 yrs<=AGE<25 yrs') site;
model EBF_8wks = treatment age_cat / dist=binomial link=log solution cl;
random Intercept / subject=site;
lsmeans treatment / cl diff=all;
estimate "Risk Ratio" treatment 1 -1 / exp cl;
nloptions tech= nrridg;
run;

/*Risk Difference*/
proc glimmix data=EBF_8wks ;
class treatment (ref='Usual Care') age_cat(ref='1 - 16 yrs<=AGE<25 yrs') site;
model EBF_8wks = treatment age_cat / dist=binomial link=identity solution cl;
random Intercept / subject=site;
lsmeans treatment / cl diff=all;
estimate "Risk Ratio" treatment 1 -1 /  cl;
nloptions tech= nrridg;
run;

/*Secondary outcome ITT analysis-Exclusive breastfeeding 16wks*/
/*Summary*/
proc sort data=EBF_16wks;
by descending EBF_16wks treatment;
run;
proc freq data=EBF_16wks order=data;
table EBF_16wks*treatment /  missing nopercent norow;
run;

proc sort data=EBF_16wks;
by descending EBF_16wks treatment;
run;
proc freq data=EBF_16wks order=data;
table EBF_16wks*treatment /   nopercent norow;
run;

/*Risk Ratio*/
proc glimmix data=EBF_16wks ;
class treatment (ref='Usual Care') age_cat(ref='1 - 16 yrs<=AGE<25 yrs') site;
model EBF_16wks = treatment age_cat / dist=binomial link=log solution cl;
random Intercept / subject=site;
smeans treatment / cl diff=all;
estimate "Risk Ratio" treatment 1 -1 / exp cl;
nloptions tech= nrridg;
run;

/*Risk Difference*/
proc glimmix data=EBF_16wks ;
class treatment (ref='Usual Care') age_cat(ref='1 - 16 yrs<=AGE<25 yrs') site;
model EBF_16wks = treatment age_cat / dist=binomial link=identity solution cl;
random Intercept / subject=site;
lsmeans treatment / cl diff=all;
estimate "Risk Ratio" treatment 1 -1 / cl;
nloptions tech= nrridg;
```

```
run;

/*Secondary outcome ITT analysis-Exclusive breastfeeding 24wks*/
/*Summary*/
proc sort data=EBF_24wks;
by descending EBF_24wks treatment;
run;
proc freq data=EBF_24wks order=data;
table EBF_24wks*treatment /  missing nopercent norow;
run;

proc sort data=EBF_24wks;
by descending EBF_24wks treatment;
run;
proc freq data=EBF_24wks order=data;
table EBF_24wks*treatment /   nopercent norow;
run;

/*Risk Ratio*/
proc glimmix data=EBF_24wks ;
class treatment (ref='Usual Care') age_cat(ref='1 - 16 yrs<=AGE<25 yrs') site;
model EBF_24wks = treatment age_cat / dist=binomial link=log solution cl;
random Intercept / subject=site;
lsmeans treatment / cl diff=all;
estimate "Risk Ratio" treatment 1 -1 / exp cl;
nloptions tech= nrridg;
run;

/*Risk Difference*/
proc glimmix data=EBF_24wks ;
class treatment (ref='Usual Care') age_cat(ref='1 - 16 yrs<=AGE<25 yrs') site;
model EBF_24wks = treatment age_cat / dist=binomial link=identity solution cl;
random Intercept / subject=site;
lsmeans treatment / cl diff=all;
estimate "Risk Ratio" treatment 1 -1 /  cl;
nloptions tech= nrridg;
run;

/*Secondary outcome ITT analysis-Maternal use of support for infant feeding 8wks*/
/*Midwife*/
proc sort data=support_8wks;
by  flwq1_midwife treatment;
run;
proc freq data=support_8wks order=data;
table flwq1_midwife*treatment /  missing nopercent norow;
run;

proc sort data=support_8wks;
by  flwq1_midwife treatment;
run;
proc freq data=support_8wks order=data;
table flwq1_midwife*treatment /   nopercent norow;
run;

/*Health visitor*/
proc sort data=support_8wks;
```

```
by  flwq1_hlthvstr treatment;
run;
proc freq data=support_8wks order=data;
table flwq1_hlthvstr*treatment /  missing nopercent norow;
run;

proc sort data=support_8wks;
by  flwq1_hlthvstr treatment;
run;
proc freq data=support_8wks order=data;
table flwq1_hlthvstr*treatment /   nopercent norow;
run;

/*GP*/
proc sort data=support_8wks;
by  flwq1_gp treatment;
run;
proc freq data=support_8wks order=data;
table flwq1_gp*treatment /  missing nopercent norow;
run;

proc sort data=support_8wks;
by  flwq1_gp treatment;
run;
proc freq data=support_8wks order=data;
table flwq1_gp*treatment /   nopercent norow;
run;

/*Practice nurse*/
proc sort data=support_8wks;
by  flwq1_prctcnurse treatment;
run;
proc freq data=support_8wks order=data;
table flwq1_prctcnurse*treatment /  missing nopercent norow;
run;

proc sort data=support_8wks;
by  flwq1_prctcnurse treatment;
run;
proc freq data=support_8wks order=data;
table flwq1_prctcnurse*treatment /   nopercent norow;
run;

/*ABA-IFH*/
proc sort data=support_8wks;
by  flwq1_abainfntfdnghlpr treatment;
run;
proc freq data=support_8wks order=data;
table flwq1_abainfntfdnghlpr*treatment /  missing nopercent norow;
run;

proc sort data=support_8wks;
by  flwq1_abainfntfdnghlpr treatment;
run;
proc freq data=support_8wks order=data;
table flwq1_abainfntfdnghlpr*treatment /   nopercent norow;
run;
```

```
/*Infant feeding counsellor or breastfeeding supporter – either 1-2-1 support or at a breastfeeding group (NOT
ABA Infant Feeding Helper)*/
proc sort data=support_8wks;
by  flwq1_lcttncnsltnt treatment;
run;
proc freq data=support_8wks order=data;
table flwq1_lcttncnsltnt*treatment /  missing nopercent norow;
run;

proc sort data=support_8wks;
by  flwq1_lcttncnsltnt treatment;
run;
proc freq data=support_8wks order=data;
table flwq1_lcttncnsltnt*treatment /   nopercent norow;
run;

/*National or local breastfeeding telephone helpline*/
proc sort data=support_8wks;
by  flwq1_lclbrsthelpline treatment;
run;
proc freq data=support_8wks order=data;
table flwq1_lclbrsthelpline*treatment /  missing nopercent norow;
run;

proc sort data=support_8wks;
by  flwq1_lclbrsthelpline treatment;
run;
proc freq data=support_8wks order=data;
table flwq1_lclbrsthelpline*treatment /   nopercent norow;
run;

/*Friend(s)*/
proc sort data=support_8wks;
by  flwq1_friends treatment;
run;
proc freq data=support_8wks order=data;
table flwq1_friends*treatment /  missing nopercent norow;
run;

proc sort data=support_8wks;
by  flwq1_friends treatment;
run;
proc freq data=support_8wks order=data;
table flwq1_friends*treatment / nopercent norow;
run;

/*Family member(s)*/
proc sort data=support_8wks;
by  flwq1_fmlymmbrs treatment;
run;
proc freq data=support_8wks order=data;
table flwq1_fmlymmbrs*treatment / missing nopercent norow;
run;

proc sort data=support_8wks;
by  flwq1_fmlymmbrs treatment;
```

```
run;
proc freq data=support_8wks order=data;
table flwq1_fmlymmbrs*treatment /nopercent norow;
run;

/*Internet support (e.g. posting to ask for support on internet/social media, NOT general browsing of web-
based resources)*/
proc sort data=support_8wks;
by  flwq1_intrntspprt treatment;
run;
proc freq data=support_8wks order=data;
table flwq1_intrntspprt*treatment /missing nopercent norow;
run;

proc sort data=support_8wks;
by  flwq1_intrntspprt treatment;
run;
proc freq data=support_8wks order=data;
table flwq1_intrntspprt*treatment / nopercent norow;
run;

 /*Other maternal use of support for infant feeding (1st option)*/
proc sort data=support_8wks;
by  flwq1_lsmnelse1 treatment;
run;
proc freq data=support_8wks order=data;
table flwq1_lsmnelse1*treatment /missing nopercent norow;
run;

proc sort data=support_8wks;
by  flwq1_lsmnelse1 treatment;
run;
proc freq data=support_8wks order=data;
table flwq1_lsmnelse1*treatment / nopercent norow;
 run;

proc freq data=support_8wks order=data;
table flwq1_lsmnelse1txt*treatment / nopercent norow nocol;
run;

 /*Other maternal use of support for infant feeding (2nd option) */
proc sort data=support_8wks;
by  flwq1_smnelse2 treatment;
run;
proc freq data=support_8wks order=data;
table flwq1_smnelse2*treatment /missing nopercent norow;
run;

proc sort data=support_8wks;
by  flwq1_smnelse2 treatment;
run;
proc freq data=support_8wks order=data;
table flwq1_smnelse2*treatment / nopercent norow;
run;

proc freq data=support_8wks order=data;
table flwq1_smnelse2txt*treatment / nopercent norow nocol;
```

```
run;

/*Other maternal use of support for infant feeding (3rd option) */
proc sort data=support_8wks;
by  flwq1_smnelse3 treatment;
run;
proc freq data=support_8wks order=data;
table flwq1_smnelse3*treatment /missing nopercent norow;
run;

proc sort data=support_8wks;
by  flwq1_smnelse3 treatment;
run;
proc freq data=support_8wks order=data;
table flwq1_smnelse3*treatment /   nopercent norow;
run;

proc freq data=support_8wks order=data;
table flwq1_smnelse3txt*treatment /   nopercent norow nocol;
run;

/*Secondary outcome ITT analysis-Maternal use of support for infant feeding 16wks*/
/*Midwife*/
proc sort data=support_16wks;
by  flwq2_midwife treatment;
run;
proc freq data=support_16wks order=data;
table flwq2_midwife*treatment /  missing nopercent norow;
run;

proc sort data=support_16wks;
by  flwq2_midwife treatment;
run;
proc freq data=support_16wks order=data;
table flwq2_midwife*treatment /   nopercent norow;
run;

/*Health visitor*/
proc sort data=support_16wks;
by  flwq2_hlthvstr treatment;
run;
proc freq data=support_16wks order=data;
table flwq2_hlthvstr*treatment /  missing nopercent norow;
run;

proc sort data=support_16wks;
by  flwq2_hlthvstr treatment;
run;
proc freq data=support_16wks order=data;
table flwq2_hlthvstr*treatment /   nopercent norow;
run;

/*GP*/
proc sort data=support_16wks;
by  flwq2_gp treatment;
run;
proc freq data=support_16wks order=data;
```

```
table flwq2_gp*treatment /  missing nopercent norow;
run;

proc sort data=support_16wks;
by  flwq2_gp treatment;
run;
proc freq data=support_16wks order=data;
table flwq2_gp*treatment /   nopercent norow;
run;

/*Practice nurse*/
proc sort data=support_16wks;
by  flwq2_prctcnurse treatment;
run;
proc freq data=support_16wks order=data;
table flwq2_prctcnurse*treatment /  missing nopercent norow;
run;

proc sort data=support_16wks;
by  flwq2_prctcnurse treatment;
run;
proc freq data=support_16wks order=data;
table flwq2_prctcnurse*treatment /   nopercent norow;
 run;

/*ABA-IFH*/
proc sort data=support_16wks;
by  flwq2_abainfntfdnghlpr treatment;
run;
proc freq data=support_16wks order=data;
table flwq2_abainfntfdnghlpr*treatment /  missing nopercent norow;
run;

proc sort data=support_16wks;
by  flwq2_abainfntfdnghlpr treatment;
run;
proc freq data=support_16wks order=data;
table flwq2_abainfntfdnghlpr*treatment /   nopercent norow;
run;

 /*Infant feeding counsellor or breastfeeding supporter – either 1-2-1 support or at a breastfeeding group
(NOT ABA Infant Feeding Helper)*/
proc sort data=support_16wks;
by  flwq2_infntfdcnlr treatment;
run;
proc freq data=support_16wks order=data;
table flwq2_infntfdcnlr*treatment /  missing nopercent norow;
run;

proc sort data=support_16wks;
by  flwq2_infntfdcnlr treatment;
run;
proc freq data=support_16wks order=data;
table flwq2_infntfdcnlr*treatment /   nopercent norow;
run;

/*National or local breastfeeding telephone helpline*/
```

```
proc sort data=support_16wks;
by  flwq2_lclbrsthelpline treatment;
run;
proc freq data=support_16wks order=data;
table flwq2_lclbrsthelpline*treatment /  missing nopercent norow;
 run;

proc sort data=support_16wks;
by  flwq2_lclbrsthelpline treatment;
run;
proc freq data=support_16wks order=data;
table flwq2_lclbrsthelpline*treatment /   nopercent norow;
run;

/*Friend(s)*/
proc sort data=support_16wks;
by  flwq2_friends treatment;
run;
proc freq data=support_16wks order=data;
table flwq2_friends*treatment /  missing nopercent norow;
run;

proc sort data=support_16wks;
by  flwq2_friends treatment;
run;
proc freq data=support_16wks order=data;
table flwq2_friends*treatment /   nopercent norow;
run;

/*Family member(s)*/
proc sort data=support_16wks;
by  flwq2_fmlymmbrs treatment;
run;
proc freq data=support_16wks order=data;
table flwq2_fmlymmbrs*treatment /  missing nopercent norow;
run;

proc sort data=support_16wks;
by  flwq2_fmlymmbrs treatment;
run;
proc freq data=support_16wks order=data;
table flwq2_fmlymmbrs*treatment /   nopercent norow;
run;

 /*Internet support (e.g. posting to ask for support on internet/social media, NOT general browsing of web-
based resources)*/
proc sort data=support_16wks;
by  flwq2_intrntspprt treatment;
run;
proc freq data=support_16wks order=data;
table flwq2_intrntspprt*treatment /  missing nopercent norow;
run;

proc sort data=support_16wks;
by  flwq2_intrntspprt treatment;
run;
proc freq data=support_16wks order=data;
```

```
table flwq2_intrntspprt*treatment /   nopercent norow;
run;

/*1st option of other support*/
proc sort data=support_16wks;
by  flwq2_lsmnelse1 treatment;
run;
proc freq data=support_16wks order=data;
table flwq2_lsmnelse1*treatment /  missing nopercent norow;
run;

proc sort data=support_16wks;
by  flwq2_lsmnelse1 treatment;
run;
proc freq data=support_16wks order=data;
table flwq2_lsmnelse1*treatment /   nopercent norow;
run;

proc freq data=support_16wks order=data;
table flwq2_lsmnelse1txt*treatment /   nopercent norow nocol;
 run;

/*2nd option of other support*/
proc sort data=support_16wks;
by  flwq2_smnelse2 treatment;
run;
proc freq data=support_16wks order=data;
table flwq2_smnelse2*treatment /  missing nopercent norow;
run;

proc sort data=support_16wks;
by  flwq2_smnelse2 treatment;
run;
proc freq data=support_16wks order=data;
table flwq2_smnelse2*treatment /   nopercent norow;
 run;
proc freq data=support_16wks order=data;
 table flwq2_smnelse2txt*treatment /   nopercent norow nocol;
run;

/*3rd option of other support*/
proc sort data=support_16wks;
by  flwq2_smnelse3 treatment;
run;
proc freq data=support_16wks order=data;
table flwq2_smnelse3*treatment /  missing nopercent norow;
run;

proc sort data=support_16wks;
by  flwq2_smnelse3 treatment;
run;
proc freq data=support_16wks order=data;
table flwq2_smnelse3*treatment /   nopercent norow;
run;

proc freq data=support_16wks order=data;
table flwq2_smnelse3txt*treatment /   nopercent norow nocol;
```

```
run;

/*Secondary outcome ITT analysis-Formula feeding practices 8wks*/
data Formula_feed_8wks;
set clean.Formula_feed_8wks;
if flwq1_milklst24hrs in (2,3) ;
run;

/*Making one feed at a time*/
proc sort data=Formula_feed_8wks;
by descending one_feed treatment;
run;
proc freq data=Formula_feed_8wks order=data;
table one_feed*treatment /  missing nopercent norow;
run;

proc sort data=Formula_feed_8wks;
by descending one_feed treatment;
run;
proc freq data=Formula_feed_8wks order=data;
table one_feed*treatment /   nopercent norow;
run;

/*Correct water temperature  */
proc sort data=Formula_feed_8wks;
by descending correct_water treatment;
run;
proc freq data=Formula_feed_8wks order=data;
table correct_water*treatment /  missing nopercent norow;
run;

proc sort data=Formula_feed_8wks;
by descending correct_water treatment;
run;
proc freq data=Formula_feed_8wks order=data;
table correct_water*treatment /   nopercent norow;
run;

/*Adding formula powder after water*/
proc sort data=Formula_feed_8wks;
by descending formula_aft_water treatment;
run;
proc freq data=Formula_feed_8wks order=data;
table formula_aft_water*treatment /  missing nopercent norow;
run;

proc sort data=Formula_feed_8wks;
by descending formula_aft_water treatment;
run;
proc freq data=Formula_feed_8wks order=data;
table formula_aft_water*treatment /   nopercent norow;
run;

/*Making up formula when needed when out of the home*/
proc sort data=Formula_feed_8wks;
by descending formula_needed treatment;
run;
```

```
proc freq data=Formula_feed_8wks order=data;
table formula_needed*treatment /  missing nopercent norow;
run;

proc sort data=Formula_feed_8wks;
by descending formula_needed treatment;
run;
proc freq data=Formula_feed_8wks order=data;
table formula_needed*treatment /   nopercent norow;
 run;

/*Keeping milk chilled when out of the home*/
proc sort data=Formula_feed_8wks;
by descending milk_chilled treatment;
run;
proc freq data=Formula_feed_8wks order=data;
table milk_chilled*treatment /  missing nopercent norow;
run;

proc sort data=Formula_feed_8wks;
by descending milk_chilled treatment;
run;
proc freq data=Formula_feed_8wks order=data;
table milk_chilled*treatment /   nopercent norow;
run;

/*Making formula with hot water when out of the home*/
proc sort data=Formula_feed_8wks;
by descending hot_water treatment;
run;
proc freq data=Formula_feed_8wks order=data;
table hot_water*treatment /  missing nopercent norow;
run;

proc sort data=Formula_feed_8wks;
by descending hot_water treatment;
run;
proc freq data=Formula_feed_8wks order=data;
table hot_water*treatment /  nopercent norow;
run;

/*Sterilising bottles using recommended methods*/
proc sort data=Formula_feed_8wks;
by descending Sterilising_bottles treatment;
run;
proc freq data=Formula_feed_8wks order=data;
table Sterilising_bottles*treatment /  missing nopercent norow;
run;

proc sort data=Formula_feed_8wks;
by descending Sterilising_bottles treatment;
run;
proc freq data=Formula_feed_8wks order=data;
table Sterilising_bottles*treatment /  nopercent norow;
run;

/*Secondary outcome ITT analysis-Formula feeding practices 16wks*/
```

```
data Formula_feed_16wks;
set clean.Formula_feed_16wks;
if flwq2_milklst24hrs in (2,3);
run;

/*Making one feed at a time*/
proc sort data=Formula_feed_16wks;
by descending one_feed treatment;
run;
proc freq data=Formula_feed_16wks order=data;
table one_feed*treatment /  missing nopercent norow;
run;

proc sort data=Formula_feed_16wks;
by descending one_feed treatment;
run;
proc freq data=Formula_feed_16wks order=data;
table one_feed*treatment /   nopercent norow;
run;

/*Correct water temperature  */
proc sort data=Formula_feed_16wks;
by descending correct_water treatment;
run;
proc freq data=Formula_feed_16wks order=data;
table correct_water*treatment /  missing nopercent norow;
run;

proc sort data=Formula_feed_16wks;
by descending correct_water treatment;
run;
proc freq data=Formula_feed_16wks order=data;
table correct_water*treatment /   nopercent norow;
run;

/*Adding formula powder after water  */
proc sort data=Formula_feed_16wks;
by descending formula_aft_water treatment;
run;
proc freq data=Formula_feed_16wks order=data;
table formula_aft_water*treatment /  missing nopercent norow;
run;

proc sort data=Formula_feed_16wks;
by descending formula_aft_water treatment;
run;
proc freq data=Formula_feed_16wks order=data;
table formula_aft_water*treatment /   nopercent norow;
run;

/*Making up formula when needed when out of the home*/
proc sort data=Formula_feed_16wks;
by descending formula_needed treatment;
run;
proc freq data=Formula_feed_16wks order=data;
table formula_needed*treatment /  missing nopercent norow;
run;
```

```
proc sort data=Formula_feed_16wks;
by descending formula_needed treatment;
run;
proc freq data=Formula_feed_16wks order=data;
table formula_needed*treatment /   nopercent norow;
run;

/*Keeping milk chilled when out of the home*/
proc sort data=Formula_feed_16wks;
by descending milk_chilled treatment;
run;
proc freq data=Formula_feed_16wks order=data;
table milk_chilled*treatment /  missing nopercent norow;
run;

proc sort data=Formula_feed_16wks;
by descending milk_chilled treatment;
run;
proc freq data=Formula_feed_16wks order=data;
table milk_chilled*treatment /   nopercent norow;
run;

/*Making formula with hot water when out of the home*/
proc sort data=Formula_feed_16wks;
by descending hot_water treatment;
run;
proc freq data=Formula_feed_16wks order=data;
table hot_water*treatment /  missing nopercent norow;
run;

proc sort data=Formula_feed_16wks;
by descending hot_water treatment;
run;
proc freq data=Formula_feed_16wks order=data;
table hot_water*treatment /   nopercent norow;
run;

/*Sterilising bottles using recommended methods*/
proc sort data=Formula_feed_16wks;
by descending Sterilising_bottles treatment;
run;
proc freq data=Formula_feed_16wks order=data;
table Sterilising_bottles*treatment /  missing nopercent norow;
run;

proc sort data=Formula_feed_16wks;
by descending Sterilising_bottles treatment;
run;
proc freq data=Formula_feed_16wks order=data;
table Sterilising_bottles*treatment /   nopercent norow;
run;

/*Secondary outcome ITT analysis-Tongue tie 8wks*/
/*Number of babies diagnosed with tongue-tie*/
proc sort data=tongue_tie_8wks;
by descending flwq1_toungetied treatment;
```

```
run;
proc freq data=tongue_tie_8wks order=data;
table flwq1_toungetied*treatment /  missing nopercent norow;
run;

proc sort data=tongue_tie_8wks;
by descending flwq1_toungetied treatment;
run;
proc freq data=tongue_tie_8wks order=data;
table flwq1_toungetied*treatment /   nopercent norow;
run;

/*Among the babies diagnosed with tongue-tie, how many had a frenotomy*/
proc sort data=tongue_tie_8wks;
by descending flwq1_frenotomy treatment;
run;
proc freq data=tongue_tie_8wks order=data;
table flwq1_frenotomy*treatment /  missing nopercent norow;
where flwq1_toungetied=1 ;
run;

proc sort data=tongue_tie_8wks;
by descending flwq1_frenotomy treatment;
run;
proc freq data=tongue_tie_8wks order=data;
table flwq1_frenotomy*treatment /   nopercent norow;
where flwq1_toungetied=1 ;
run;

/*Secondary outcome ITT analysis-Any infant hospital admission up to 16wks*/
/*Any infant hospital admission Up to 8 weeks*/
proc sort data=Inf_admiss_16wks;
by descending Inf_admiss_8wks treatment;
run;
proc freq data=Inf_admiss_16wks order=data;
table Inf_admiss_8wks*treatment /  missing nopercent norow;
run;

proc sort data=Inf_admiss_16wks;
by descending Inf_admiss_8wks treatment;
run;
proc freq data=Inf_admiss_16wks order=data;
table Inf_admiss_8wks*treatment /   nopercent norow;
run;

/*Any infant hospital admission 8 - 16 weeks*/
proc sort data=Inf_admiss_16wks;
by descending Inf_admiss_8_16wks treatment;
run;
proc freq data=Inf_admiss_16wks order=data;
table Inf_admiss_8_16wks*treatment /  missing nopercent norow;
run;

proc sort data=Inf_admiss_16wks;
by descending Inf_admiss_8_16wks treatment;
run;
proc freq data=Inf_admiss_16wks order=data;
```

```
table Inf_admiss_8_16wks*treatment /   nopercent norow;
run;

/*Any infant hospital admission Up to 16weeks*/
proc sort data=Inf_admiss_16wks;
by descending Inf_admiss_16wks treatment;
run;
proc freq data=Inf_admiss_16wks order=data;
table Inf_admiss_16wks*treatment /  missing nopercent norow;
run;

proc sort data=Inf_admiss_16wks;
by descending Inf_admiss_16wks treatment;
run;
proc freq data=Inf_admiss_16wks order=data;
table Inf_admiss_16wks*treatment /   nopercent norow;
run;

/*Risk Ratio*/
proc glimmix data=Inf_admiss_16wks ;
class treatment (ref='Usual Care') age_cat (ref='1 - 16 yrs<=AGE<25 yrs') ;
model Inf_admiss_16wks = treatment age_cat/ dist=binomial link=log solution cl;
lsmeans treatment / cl diff=all ;
estimate "Risk Ratio" treatment 1 -1 / exp cl;
nloptions tech= nrridg;
run;

/*Risk Difference*/
proc glimmix data=Inf_admiss_16wks ;
class treatment (ref='Usual Care') age_cat(ref='1 - 16 yrs<=AGE<25 yrs') ;
model Inf_admiss_16wks = treatment age_cat / dist=binomial link=identity solution cl;
lsmeans treatment / cl diff=all;
estimate "risk ratio" treatment 1 -1 /  cl;
nloptions tech= nrridg;
run;

/*Secondary outcome ITT analysis-GAD_7 8wks*/
/*GAD-7 baseline score */
proc means data=Gad7_8wks n mean std min max median Q1 Q3;
class treatment;
var Gad7_base ;
run;

Proc sort data=Gad7_8wks;
by treatment;
run;
proc sgpanel data=Gad7_8wks;
panelby treatment;
histogram Gad7_base;
density Gad7_base;
run;
/*GAD-7 score*/
proc means data=Gad7_8wks n mean std min max median Q1 Q3;
class treatment;
var Gad7_8wks_ts ;
run;
```

```
Proc sort data=Gad7_8wks;
by treatment;
run;
proc sgpanel data=Gad7_8wks;
panelby treatment;
histogram Gad7_8wks_ts;
density Gad7_8wks_ts;
run;

/*Mean difference*/
ods graphics on;
proc glimmix data=Gad7_8wks plots=studentpanel(conditional marginal);
class treatment site;
model Gad7_8wks_ts = treatment age Gad7_base / dist=gaussian link=identity solution cl;
random Intercept / subject=site ;
estimate 'Mean Difference Intervention vs. Control' treatment 1 -1/cl ;
lsmeans treatment/PDIFF cl ;
output out=residuals predicted=pred resid=resid student=student;
run;
ods graphics off;

/*Secondary outcome ITT analysis-GAD_7 16wks*/
/*GAD-7 baseline score*/
proc means data=Gad7_16wks n mean std min max median Q1 Q3;
class treatment;
var Gad7_base ;
run;

Proc sort data=Gad7_16wks;
by treatment;
run;
proc sgpanel data=Gad7_16wks;
panelby treatment;
histogram Gad7_base;
density Gad7_base;
run;

/*GAD-7 score*/
proc means data=Gad7_16wks n mean std min max median Q1 Q3;
class treatment;
var Gad7_16wks_ts ;
run;

Proc sort data=Gad7_16wks;
by treatment;
run;
proc sgpanel data=Gad7_16wks;
panelby treatment;
histogram Gad7_16wks_ts;
density Gad7_16wks_ts;
run;

/*Mean difference*/
ods graphics on;
proc glimmix data=Gad7_16wks plots=studentpanel(conditional marginal);
class treatment site;
model Gad7_16wks_ts = treatment age Gad7_base / dist=gaussian link=identity solution cl  ;
```

```
random Intercept / subject=site ;
lsmeans treatment/PDIFF  cl ;
output out=residuals predicted=pred resid=resid student=student ;
run;
ods graphics off;

/*Secondary outcome ITT analysis-EQ5D5L_8wks*/
/*EQ5D baseline score summary*/
Proc sort data=EQ5D5L_8wks;
by treatment;
run;
proc sgpanel data=EQ5D5L_8wks;
panelby treatment;
histogram EQ5D_base;
density EQ5D_base;
run;

proc means data=EQ5D5L_8wks n mean std min max median Q1 Q3;
class treatment;
var EQ5D_base ;
run;

/*EQ5D score at 8 wks summary*/
Proc sort data=EQ5D5L_8wks;
by treatment;
run;
proc sgpanel data=EQ5D5L_8wks;
panelby treatment;
histogram EQ5D5L_8wks_is;
density EQ5D5L_8wks_is;
run;

proc means data=EQ5D5L_8wks n mean std min max median Q1 Q3;
class treatment;
var EQ5D5L_8wks_is ;
run;

/*Mean difference*/
ods graphics on;
proc glimmix data=EQ5D5L_8wks plots=studentpanel(conditional marginal);
class treatment site;
model EQ5D5L_8wks_is = treatment age EQ5D_base  / dist=gaussian link=identity solution cl;
random Intercept / subject=site;
estimate 'Mean Difference Intervention vs. Control' treatment 1 -1/cl;
lsmeans treatment/PDIFF cl ;
output out=residuals predicted=pred resid=resid student=student;
run;
ods graphics off;

/*Secondary outcome ITT analysis-EQ5D5L_16wks*/
/*EQ5D baseline score */
Proc sort data=EQ5D5L_16wks;
by treatment;
run;
proc sgpanel data=EQ5D5L_16wks;
panelby treatment;
histogram EQ5D_base;
```

```
density EQ5D_base;
run;

proc means data=EQ5D5L_16wks n mean std min max median Q1 Q3;
class treatment;
var EQ5D_base ;
run;

/*EQ5D score at 16 wks*/
Proc sort data=EQ5D5L_16wks;
by treatment;
run;
proc sgpanel data=EQ5D5L_16wks;
panelby treatment;
histogram EQ5D5L_16wks_is;
density EQ5D5L_16wks_is;
run;

proc means data=EQ5D5L_16wks n mean std min max median Q1 Q3;
class treatment;
var EQ5D5L_16wks_is ;
run;

/*Mean difference*/
ods graphics on;
proc glimmix data=EQ5D5L_16wks plots=studentpanel(conditional marginal);
class treatment site;
model EQ5D5L_16wks_is = treatment age EQ5D_base / dist=gaussian link=identity solution cl;
random Intercept / subject=site;
estimate 'Mean Difference Intervention vs. Control' treatment 1 -1/cl;
lsmeans treatment/PDIFF cl ;
output out=residuals predicted=pred resid=resid student=student;
run;
ods graphics off;

/*Secondary outcome ITT analysis-MOS_8wks*/
/*MOS baseline score summary*/
Proc sort data=MOS_8wks;
by treatment;
run;
proc sgpanel data=MOS_8wks;
panelby treatment;
histogram MOS_base;
density MOS_base;
run;

proc means data=MOS_8wks n mean std min max median Q1 Q3;
class treatment;
var MOS_base ;
run;

/*MOS score at 8 wks summary*/
Proc sort data=MOS_8wks;
by treatment;
run;
proc sgpanel data=MOS_8wks;
panelby treatment;
```

```
histogram MOS_8wks_ts;
density MOS_8wks_ts;
run;

proc means data=MOS_8wks n mean std min max median Q1 Q3;
class treatment;
var MOS_8wks_ts ;
run;

/*Mean difference*/
ods graphics on;
proc glimmix data=MOS_8wks plots=studentpanel(conditional marginal);
class treatment site;
model MOS_8wks_ts = treatment age MOS_base / dist=gaussian link=identity solution cl;
random Intercept / subject=site ;
estimate 'Mean Difference Intervention vs. Control' treatment 1 -1/cl ;
lsmeans treatment/PDIFF  cl ;
output out=residuals predicted=pred resid=resid student=student;
run;
ods graphics off;

/*Secondary outcome ITT analysis-MOS_16wks*/
/*MOS baseline score summary*/
Proc sort data=MOS_16wks;
by treatment;
run;
proc sgpanel data=MOS_16wks;
panelby treatment;
histogram MOS_base;
density MOS_base;
run;

proc means data=MOS_16wks n mean std min max median Q1 Q3;
class treatment;
var MOS_base ;
run;

/*MOS score at 16 wks summary*/
Proc sort data=MOS_16wks;
by treatment;
run;
proc sgpanel data=MOS_16wks;
panelby treatment;
histogram MOS_16wks_ts;
density MOS_16wks_ts;
run;

proc means data=MOS_16wks n mean std min max median Q1 Q3;
class treatment;
var MOS_16wks_ts ;
run;

/*Mean difference*/
ods graphics on;
proc glimmix data=MOS_16wks plots=studentpanel(conditional marginal);
class treatment site;
model MOS_16wks_ts = treatment age MOS_base / dist=gaussian link=identity solution cl  ;
```

```
random Intercept / subject=site ;
estimate 'Mean Difference Intervention vs. Control' treatment 1 -1/  cl ;
lsmeans treatment/PDIFF  cl ;
output out=residuals predicted=pred resid=resid student=student ;
run;
ods graphics off;

/*Secondary outcome ITT analysis-TTcease_16wks*/
/*Data exploration with proc univariate and proc corr*/
proc corr data = TTCease_16wks plots(maxpoints=none)=matrix(histogram);
var TTCease_16wks treatment age site cluster_IFH;
run;

/*logrank - Kaplan Meier plot */
ods output homstats=rankstatistics(keep=treatment logrank) logrankhomcov=logrankvariance
(keep=treatment);
proc lifetest data=TTCease_16wks notable plots=survival(atrisk cb) method=km;
time TTCease_16wks*status(0);
strata treatment;
run;

proc lifetest data=TTCease_16wks notable plots=(s, lls);
time TTCease_16wks*status(0);
strata treatment;
run;

/*Including Time Dependent Covariates in the Cox Model*/
proc phreg data=TTCease_16wks;
model TTCease_16wks*status(0) = treatment age site treatmentt aget sitet;
treatmentt=treatment*log(TTCease_16wks);
aget=age*log(TTCease_16wks);
sitet=site*log(TTCease_16wks);
proportionality_test: test treatment, age, site;
run;

/*Checking the proportional hazards assumption*/
proc phreg data=TTCease_16wks;
class treatment;
model TTCease_16wks*status(0) = treatment age /Risklimits alpha=0.05;
assess var=(age) ph / RESAMPLE;
run;

/*Summary*/
proc sort  data=TTCease_16wks  ;
by   status;
run;
proc freq data=TTCease_16wks order=data;
table status*treatment/norow nopercent;
run;

proc sort  data=TTCease_16wks  ;
by   status;
run;
proc freq data=TTCease_16wks order=data;
table status*treatment/norow missing nopercent;
run;
```

```
proc means data=TTCease_16wks n mean min max median Q1 Q3 ;
class treatment;
var TTCease_16wks;
where status=1;
run;

/*Unadjusted cox model*/
proc phreg data=TTCease_16wks;
class treatment;
model TTCease_16wks*status(0) = treatment/Risklimits alpha=0.05;
run;

/*Adjusted cox model*/
proc phreg data=TTCease_16wks;
class treatment site ;
model TTCease_16wks*status(0) = treatment age /Risklimits alpha=0.05;
random site ;
run;

/*Adjusted cox model (using option: ties=efrom)*/
proc phreg data=TTCease_16wks;
class treatment site ;
model TTCease_16wks*status(0) = treatment age /Risklimits alpha=0.05  ties=efron;
random site ;
run;

/*Other analyses to address the non-proportional issue of age*/
/* Adjusted cox model -- excluding age*/
proc phreg data=TTCease_16wks;
class treatment site;
model TTCease_16wks*status(0) = treatment /Risklimits alpha=0.05;
random site ;
run;

/*Adjusted cox model -- stratifying by age*/
 proc means data=TTCease_16wks N mean std  min max  Q1 median Q3;
var age;
run;

proc phreg data=TTCease_16wks;
class treatment site;
model TTCease_16wks*status(0) = treatment age /Risklimits alpha=0.05;
strata age_cat;
random site ;
run;

/*Secondary outcome ITT analysis-TTcease_any_16wks*/
/*Data exploration with proc univariate and proc corr*/
proc corr data = TTcease_any_16wks plots(maxpoints=none)=matrix(histogram);
var TTcease_any_16wks treatment age site cluster_IFH;
run;

/* logrank - Kaplan Meier plot */
ods output homstats=rankstatistics(keep=treatment logrank) logrankhomcov=logrankvariance
(keep=treatment);
proc lifetest data=TTcease_any_16wks notable plots=survival(atrisk cb) method=km;
```

```
time TTcease_any_16wks*status(0);
strata treatment;
run;

proc lifetest data=TTcease_any_16wks notable plots=(s, lls);
time TTcease_any_16wks*status(0);
strata treatment;
run;

/*Including Time Dependent Covariates in the Cox Model*/
proc phreg data=TTcease_any_16wks;
model TTCease_any_16wks*status(0) = treatment age site treatmentt aget sitet;
treatmentt=treatment*log(TTCease_any_16wks);
aget=age*log(TTCease_any_16wks);
sitet=site*log(TTCease_any_16wks);
proportionality_test: test treatment, age, site;
run;

/*Checking the proportional hazards assumption*/
proc phreg data=TTCease_any_16wks;
class treatment;
model TTCease_any_16wks*status(0) = treatment age /Risklimits alpha=0.05;
assess var=(age) ph / RESAMPLE;
run;

/*Summary*/
proc sort  data=TTCease_any_16wks  ;
by   status;
run;
proc freq data=TTCease_any_16wks order=data;
table status*treatment/norow nopercent;
run;

proc means data=TTCease_any_16wks n mean min max median Q1 Q3 ;
class treatment;
var TTCease_any_16wks;
where status=1;
run;

/*Unadjusted cox model*/
proc phreg data=TTCease_any_16wks;
class treatment;
model TTCease_any_16wks*status(0) = treatment/Risklimits alpha=0.05;
run;

/*Adjusted cox model*/
proc phreg data=TTCease_any_16wks;
class treatment site ;
model TTCease_any_16wks*status(0) = treatment age /Risklimits alpha=0.05;
random site ;
run;

/*Other analyses to address the non-proportional issue of age*/
/* Adjusted cox model -- excluding age*/
proc phreg data=TTCease_any_16wks;
class treatment site;
model TTCease_any_16wks*status(0) = treatment /Risklimits alpha=0.05;
```

```
random site ;
run;

/*Adjusted cox model -- stratifying by age*/
 proc means data=TTCease_any_16wks N mean std  min max  Q1 median Q3;
var age;
run;

proc phreg data=TTCease_any_16wks;
class treatment site;
model TTCease_any_16wks*status(0) = treatment age /Risklimits alpha=0.05;
strata age_cat;
random site ;
run;
```
